# Supplementary material for: Effect of isolation on coat colour polymorphism of Polynesian rats in Island Southeast Asia and the Pacific
Source: PeerJ. 2019 May 8;7:e6894. doi: 10.7717/peerj.6894 (PMC6511229; doi:10.7717/peerj.6894)
Supplement: Data S1 — This file contains specimen numbers of skins of Rattus exulans examined at the American Museum of Natural History (New York; AMNH), the Smithsonian Institute, Natural History Museum (Washington; USNM) and Te Papa Tongarewa Museum of New Zealand (Wellington; NMNZ). [file peerj-07-6894-s001.pdf]

Effect of isolation on coat colour polymorphism of wild populations of Polynesian rats (*Rattus exulans*) in Island Southeast Asia and the Pacific

Alexandra A.E. van der Geer

Naturalis Biodiversity Center
Leiden
the Netherlands

alexandra.vandergeer@naturalis.nl

DATA FILE S1

This file contains specimen numbers of skins of *Rattus exulans* examined at the American Museum of Natural History (New York; AMNH), the Smithsonian Institute, Natural History Museum (Washington; USNM) and Te Papa Tongarewa Museum of New Zealand (Wellington; NMNZ).

Institute	Specimen	Island	Sex	Biogeographical region
AMNH	M-267705	Ambon	Female	Wallacea
AMNH	M-102154	Bali	Female	Lesser Sunda Islands
AMNH	M-107976	Bali	Female	Lesser Sunda Islands
AMNH	M-103508	Borneo	Female	Greater Sunda Islands
AMNH	M-103509	Borneo	Female	Greater Sunda Islands
AMNH	M-103510	Borneo	Female	Greater Sunda Islands
AMNH	M-103511	Borneo	Female	Greater Sunda Islands
AMNH	M-103512	Borneo	Female	Greater Sunda Islands
AMNH	M-103513	Borneo	Female	Greater Sunda Islands
AMNH	M-103514	Borneo	Female	Greater Sunda Islands
AMNH	M-103515	Borneo	Female	Greater Sunda Islands
AMNH	M-103517	Borneo	Female	Greater Sunda Islands
AMNH	M-103827	Borneo	Female	Greater Sunda Islands
AMNH	M-103828	Borneo	Female	Greater Sunda Islands
AMNH	M-103831	Borneo	Female	Greater Sunda Islands
AMNH	M-103832	Borneo	Female	Greater Sunda Islands
AMNH	M-103834	Borneo	Female	Greater Sunda Islands
AMNH	M-103835	Borneo	Female	Greater Sunda Islands
AMNH	M-103836	Borneo	Female	Greater Sunda Islands
AMNH	M-103837	Borneo	Female	Greater Sunda Islands
AMNH	M-103838	Borneo	Female	Greater Sunda Islands
AMNH	M-103839	Borneo	Female	Greater Sunda Islands
AMNH	M-103840	Borneo	Female	Greater Sunda Islands
AMNH	M-103926	Borneo	Female	Greater Sunda Islands
AMNH	M-103927	Borneo	Female	Greater Sunda Islands
AMNH	M-103930	Borneo	Female	Greater Sunda Islands
AMNH	M-106126	Borneo	Female	Greater Sunda Islands
AMNH	M-106128	Borneo	Female	Greater Sunda Islands
AMNH	M-106192	Borneo	Female	Greater Sunda Islands
AMNH	M-106193	Borneo	Female	Greater Sunda Islands
AMNH	M-106195	Borneo	Female	Greater Sunda Islands
AMNH	M-106196	Borneo	Female	Greater Sunda Islands
AMNH	M-106197	Borneo	Female	Greater Sunda Islands
AMNH	M-106199	Borneo	Female	Greater Sunda Islands
AMNH	M-106200	Borneo	Female	Greater Sunda Islands
AMNH	M-106206	Borneo	Female	Greater Sunda Islands
AMNH	M-107025	Borneo	Female	Greater Sunda Islands
AMNH	M-107026	Borneo	Female	Greater Sunda Islands
AMNH	M-107027	Borneo	Female	Greater Sunda Islands
AMNH	M-107031	Borneo	Female	Greater Sunda Islands
AMNH	M-107034	Borneo	Female	Greater Sunda Islands
AMNH	M-107035	Borneo	Female	Greater Sunda Islands
AMNH	M-107037	Borneo	Female	Greater Sunda Islands
AMNH	M-107045	Borneo	Female	Greater Sunda Islands
AMNH	M-107047	Borneo	Female	Greater Sunda Islands
AMNH	M-107048	Borneo	Female	Greater Sunda Islands
AMNH	M-107049	Borneo	Female	Greater Sunda Islands
AMNH	M-163811	Burma	Female	Mainland
AMNH	M-163812	Burma	Female	Mainland
AMNH	M-163814	Burma	Female	Mainland
AMNH	M-159741	Fergusson Island	Female	Melanesia
AMNH	M-159742	Fergusson Island	Female	Melanesia
AMNH	M-159743	Fergusson Island	Female	Melanesia
AMNH	M-157935	Goodenough Island	Female	Melanesia
AMNH	M-157936	Goodenough Island	Female	Melanesia
AMNH	M-157937	Goodenough Island	Female	Melanesia
AMNH	M-157938	Goodenough Island	Female	Melanesia
AMNH	M-157939	Goodenough Island	Female	Melanesia
AMNH	M-157940	Goodenough Island	Female	Melanesia
AMNH	M-157941	Goodenough Island	Female	Melanesia
AMNH	M-157944	Goodenough Island	Female	Melanesia
AMNH	M-157945	Goodenough Island	Female	Melanesia
AMNH	M-157946	Goodenough Island	Female	Melanesia
AMNH	M-157947	Goodenough Island	Female	Melanesia
AMNH	M-101297	Halmahera	Female	Wallacea
AMNH	M-101301	Halmahera	Female	Wallacea

Institute	Specimen	Island	Sex	Biogeographical region
AMNH	M-101306	Halmahera	Female	Wallacea
AMNH	M-267656	Halmahera	Female	Wallacea
AMNH	M-267658	Halmahera	Female	Wallacea
AMNH	M-267682	Halmahera	Female	Wallacea
AMNH	M-267685	Halmahera	Female	Wallacea
AMNH	M-267690	Halmahera	Female	Wallacea
AMNH	M-267691	Halmahera	Female	Wallacea
AMNH	M-267696	Halmahera	Female	Wallacea
AMNH	M-143882	Hawaii	Female	Polynesia
AMNH	M-143883	Hawaii	Female	Polynesia
AMNH	M-102004	Java	Female	Greater Sunda Islands
AMNH	M-106690	Java	Female	Greater Sunda Islands
AMNH	M-68741	Kanton Island	Female	Micronesia
AMNH	M-159730	Kiriwina Island	Female	Melanesia
AMNH	M-159731	Kiriwina Island	Female	Melanesia
AMNH	M-184590	Luzon	Female	Wallacea
AMNH	M-187185	Luzon	Female	Wallacea
AMNH	M-187189	Luzon	Female	Wallacea
AMNH	M-187195	Luzon	Female	Wallacea
AMNH	M-187196	Luzon	Female	Wallacea
AMNH	M-153292	Malenge Island	Female	Wallacea
AMNH	M-153293	Malenge Island	Female	Wallacea
AMNH	M-153294	Malenge Island	Female	Wallacea
AMNH	M-153295	Malenge Island	Female	Wallacea
AMNH	M-153301	Malenge Island	Female	Wallacea
AMNH	M-153303	Malenge Island	Female	Wallacea
AMNH	M-153305	Malenge Island	Female	Wallacea
AMNH	M-153306	Malenge Island	Female	Wallacea
AMNH	M-153310	Malenge Island	Female	Wallacea
AMNH	M-153311	Malenge Island	Female	Wallacea
AMNH	M-153314	Malenge Island	Female	Wallacea
AMNH	M-159733	Misima Island	Female	Melanesia
AMNH	M-159734	Misima Island	Female	Melanesia
AMNH	M-159735	Misima Island	Female	Melanesia
AMNH	M-207544	Negros	Female	Wallacea
AMNH	M-207547	Negros	Female	Wallacea
AMNH	M-207551	Negros	Female	Wallacea
AMNH	M-207552	Negros	Female	Wallacea
AMNH	M-207553	Negros	Female	Wallacea
AMNH	M-194383	New Britain Island	Female	Melanesia
AMNH	M-194384	New Britain Island	Female	Melanesia
AMNH	M-194385	New Britain Island	Female	Melanesia
AMNH	M-194388	New Britain Island	Female	Melanesia
AMNH	M-194390	New Britain Island	Female	Melanesia
AMNH	M-194392	New Britain Island	Female	Melanesia
AMNH	M-194393	New Britain Island	Female	Melanesia
AMNH	M-194394	New Britain Island	Female	Melanesia
AMNH	M-222325	New Britain Island	Female	Melanesia
AMNH	M-222326	New Britain Island	Female	Melanesia
AMNH	M-222327	New Britain Island	Female	Melanesia
AMNH	M-222330	New Britain Island	Female	Melanesia
AMNH	M-222332	New Britain Island	Female	Melanesia
AMNH	M-222333	New Britain Island	Female	Melanesia
AMNH	M-222402	New Britain Island	Female	Melanesia
AMNH	M-104237	New Guinea	Female	Melanesia
AMNH	M-104247	New Guinea	Female	Melanesia
AMNH	M-108216	New Guinea	Female	Melanesia
AMNH	M-108222	New Guinea	Female	Melanesia
AMNH	M-108223	New Guinea	Female	Melanesia
AMNH	M-108224	New Guinea	Female	Melanesia
AMNH	M-108225	New Guinea	Female	Melanesia
AMNH	M-110089	New Guinea	Female	Melanesia
AMNH	M-110092	New Guinea	Female	Melanesia
AMNH	M-110098	New Guinea	Female	Melanesia
AMNH	M-110099	New Guinea	Female	Melanesia
AMNH	M-110102	New Guinea	Female	Melanesia

Institute	Specimen	Island	Sex	Biogeographical region
AMNH	M-110108	New Guinea	Female	Melanesia
AMNH	M-110109	New Guinea	Female	Melanesia
AMNH	M-110117	New Guinea	Female	Melanesia
AMNH	M-157873	New Guinea	Female	Melanesia
AMNH	M-157874	New Guinea	Female	Melanesia
AMNH	M-157875	New Guinea	Female	Melanesia
AMNH	M-157876	New Guinea	Female	Melanesia
AMNH	M-157877	New Guinea	Female	Melanesia
AMNH	M-157878	New Guinea	Female	Melanesia
AMNH	M-157879	New Guinea	Female	Melanesia
AMNH	M-157880	New Guinea	Female	Melanesia
AMNH	M-157881	New Guinea	Female	Melanesia
AMNH	M-157917	New Guinea	Female	Melanesia
AMNH	M-157918	New Guinea	Female	Melanesia
AMNH	M-157919	New Guinea	Female	Melanesia
AMNH	M-157921	New Guinea	Female	Melanesia
AMNH	M-157922	New Guinea	Female	Melanesia
AMNH	M-157923	New Guinea	Female	Melanesia
AMNH	M-157924	New Guinea	Female	Melanesia
AMNH	M-191547	New Guinea	Female	Melanesia
AMNH	M-191548	New Guinea	Female	Melanesia
AMNH	M-191549	New Guinea	Female	Melanesia
AMNH	M-191550	New Guinea	Female	Melanesia
AMNH	M-191551	New Guinea	Female	Melanesia
AMNH	M-191552	New Guinea	Female	Melanesia
AMNH	M-191553	New Guinea	Female	Melanesia
AMNH	M-191554	New Guinea	Female	Melanesia
AMNH	M-191555	New Guinea	Female	Melanesia
AMNH	M-191556	New Guinea	Female	Melanesia
AMNH	M-191565	New Guinea	Female	Melanesia
AMNH	M-191566	New Guinea	Female	Melanesia
AMNH	M-191567	New Guinea	Female	Melanesia
AMNH	M-191568	New Guinea	Female	Melanesia
AMNH	M-191573	New Guinea	Female	Melanesia
AMNH	M-191574	New Guinea	Female	Melanesia
AMNH	M-191575	New Guinea	Female	Melanesia
AMNH	M-191576	New Guinea	Female	Melanesia
AMNH	M-191577	New Guinea	Female	Melanesia
AMNH	M-191589	New Guinea	Female	Melanesia
AMNH	M-191590	New Guinea	Female	Melanesia
AMNH	M-191591	New Guinea	Female	Melanesia
AMNH	M-191592	New Guinea	Female	Melanesia
AMNH	M-191593	New Guinea	Female	Melanesia
AMNH	M-191594	New Guinea	Female	Melanesia
AMNH	M-191598	New Guinea	Female	Melanesia
AMNH	M-191602	New Guinea	Female	Melanesia
AMNH	M-191603	New Guinea	Female	Melanesia
AMNH	M-191604	New Guinea	Female	Melanesia
AMNH	M-191605	New Guinea	Female	Melanesia
AMNH	M-191606	New Guinea	Female	Melanesia
AMNH	M-191607	New Guinea	Female	Melanesia
AMNH	M-191615	New Guinea	Female	Melanesia
AMNH	M-191616	New Guinea	Female	Melanesia
AMNH	M-191617	New Guinea	Female	Melanesia
AMNH	M-191618	New Guinea	Female	Melanesia
AMNH	M-195044	New Guinea	Female	Melanesia
AMNH	M-195047	New Guinea	Female	Melanesia
AMNH	M-195048	New Guinea	Female	Melanesia
AMNH	M-195049	New Guinea	Female	Melanesia
AMNH	M-195050	New Guinea	Female	Melanesia
AMNH	M-195051	New Guinea	Female	Melanesia
AMNH	M-195053	New Guinea	Female	Melanesia
AMNH	M-195054	New Guinea	Female	Melanesia
AMNH	M-195059	New Guinea	Female	Melanesia
AMNH	M-195060	New Guinea	Female	Melanesia
AMNH	M-195062	New Guinea	Female	Melanesia

Institute	Specimen	Island	Sex	Biogeographical region
AMNH	M-195064	New Guinea	Female	Melanesia
AMNH	M-195067	New Guinea	Female	Melanesia
AMNH	M-195068	New Guinea	Female	Melanesia
AMNH	M-195069	New Guinea	Female	Melanesia
AMNH	M-195070	New Guinea	Female	Melanesia
AMNH	M-195074	New Guinea	Female	Melanesia
AMNH	M-195075	New Guinea	Female	Melanesia
AMNH	M-195079	New Guinea	Female	Melanesia
AMNH	M-195081	New Guinea	Female	Melanesia
AMNH	M-195082	New Guinea	Female	Melanesia
AMNH	M-195088	New Guinea	Female	Melanesia
AMNH	M-195089	New Guinea	Female	Melanesia
AMNH	M-198798	New Guinea	Female	Melanesia
AMNH	M-198799	New Guinea	Female	Melanesia
AMNH	M-198800	New Guinea	Female	Melanesia
AMNH	M-198801	New Guinea	Female	Melanesia
AMNH	M-198807	New Guinea	Female	Melanesia
AMNH	M-198808	New Guinea	Female	Melanesia
AMNH	M-198809	New Guinea	Female	Melanesia
AMNH	M-198810	New Guinea	Female	Melanesia
AMNH	M-198811	New Guinea	Female	Melanesia
AMNH	M-198812	New Guinea	Female	Melanesia
AMNH	M-198813	New Guinea	Female	Melanesia
AMNH	M-222287	New Guinea	Female	Melanesia
AMNH	M-222288	New Guinea	Female	Melanesia
AMNH	M-222289	New Guinea	Female	Melanesia
AMNH	M-222291	New Guinea	Female	Melanesia
AMNH	M-222292	New Guinea	Female	Melanesia
AMNH	M-222296	New Guinea	Female	Melanesia
AMNH	M-222298	New Guinea	Female	Melanesia
AMNH	M-222300	New Guinea	Female	Melanesia
AMNH	M-222301	New Guinea	Female	Melanesia
AMNH	M-222305	New Guinea	Female	Melanesia
AMNH	M-222307	New Guinea	Female	Melanesia
AMNH	M-222310	New Guinea	Female	Melanesia
AMNH	M-222315	New Guinea	Female	Melanesia
AMNH	M-222317	New Guinea	Female	Melanesia
AMNH	M-222319	New Guinea	Female	Melanesia
AMNH	M-222322	New Guinea	Female	Melanesia
AMNH	M-222338	New Guinea	Female	Melanesia
AMNH	M-222342	New Guinea	Female	Melanesia
AMNH	M-222343	New Guinea	Female	Melanesia
AMNH	M-222345	New Guinea	Female	Melanesia
AMNH	M-222347	New Guinea	Female	Melanesia
AMNH	M-222348	New Guinea	Female	Melanesia
AMNH	M-222349	New Guinea	Female	Melanesia
AMNH	M-222351	New Guinea	Female	Melanesia
AMNH	M-222352	New Guinea	Female	Melanesia
AMNH	M-222355	New Guinea	Female	Melanesia
AMNH	M-222357	New Guinea	Female	Melanesia
AMNH	M-222358	New Guinea	Female	Melanesia
AMNH	M-222359	New Guinea	Female	Melanesia
AMNH	M-222360	New Guinea	Female	Melanesia
AMNH	M-222362	New Guinea	Female	Melanesia
AMNH	M-222366	New Guinea	Female	Melanesia
AMNH	M-222390	New Guinea	Female	Melanesia
AMNH	M-159758	Normanby Island	Female	Melanesia
AMNH	M-159759	Normanby Island	Female	Melanesia
AMNH	M-159760	Normanby Island	Female	Melanesia
AMNH	M-159761	Normanby Island	Female	Melanesia
AMNH	M-159762	Normanby Island	Female	Melanesia
AMNH	M-159763	Normanby Island	Female	Melanesia
AMNH	M-159764	Normanby Island	Female	Melanesia
AMNH	M-159765	Normanby Island	Female	Melanesia
AMNH	M-159766	Normanby Island	Female	Melanesia
AMNH	M-159767	Normanby Island	Female	Melanesia

Institute	Specimen	Island	Sex	Biogeographical region
AMNH	M-159768	Normanby Island	Female	Melanesia
AMNH	M-68748	Ofu Island	Female	Polynesia
AMNH	M-68740	Orona Island	Female	Micronesia
AMNH	M-207606	Palawan	Female	Sunda
AMNH	M-109171	Peleng Island	Female	Wallacea
AMNH	M-109172	Peleng Island	Female	Wallacea
AMNH	M-109173	Peleng Island	Female	Wallacea
AMNH	M-109174	Peleng Island	Female	Wallacea
AMNH	M-109175	Peleng Island	Female	Wallacea
AMNH	M-109181	Peleng Island	Female	Wallacea
AMNH	M-109182	Peleng Island	Female	Wallacea
AMNH	M-109183	Peleng Island	Female	Wallacea
AMNH	M-109184	Peleng Island	Female	Wallacea
AMNH	M-68747	Rose Island	Female	Polynesia
AMNH	M-159700	Rossel Island	Female	Melanesia
AMNH	M-159701	Rossel Island	Female	Melanesia
AMNH	M-159702	Rossel Island	Female	Melanesia
AMNH	M-159703	Rossel Island	Female	Melanesia
AMNH	M-159704	Rossel Island	Female	Melanesia
AMNH	M-159705	Rossel Island	Female	Melanesia
AMNH	M-159714	Rossel Island	Female	Melanesia
AMNH	M-159715	Rossel Island	Female	Melanesia
AMNH	M-159716	Rossel Island	Female	Melanesia
AMNH	M-159725	Vanatinai	Female	Melanesia
AMNH	M-159726	Vanatinai	Female	Melanesia
AMNH	M-159727	Vanatinai	Female	Melanesia
AMNH	M-159728	Vanatinai	Female	Melanesia
AMNH	M-100984	Sulawesi	Female	Wallacea
AMNH	M-100986	Sulawesi	Female	Wallacea
AMNH	M-100988	Sulawesi	Female	Wallacea
AMNH	M-100991	Sulawesi	Female	Wallacea
AMNH	M-101046	Sulawesi	Female	Wallacea
AMNH	M-101260	Sulawesi	Female	Wallacea
AMNH	M-101263	Sulawesi	Female	Wallacea
AMNH	M-101274	Sulawesi	Female	Wallacea
AMNH	M-153015	Sulawesi	Female	Wallacea
AMNH	M-153016	Sulawesi	Female	Wallacea
AMNH	M-153019	Sulawesi	Female	Wallacea
AMNH	M-153027	Sulawesi	Female	Wallacea
AMNH	M-153029	Sulawesi	Female	Wallacea
AMNH	M-153030	Sulawesi	Female	Wallacea
AMNH	M-153032	Sulawesi	Female	Wallacea
AMNH	M-153033	Sulawesi	Female	Wallacea
AMNH	M-153034	Sulawesi	Female	Wallacea
AMNH	M-153037	Sulawesi	Female	Wallacea
AMNH	M-223102	Sulawesi	Female	Wallacea
AMNH	M-223104	Sulawesi	Female	Wallacea
AMNH	M-223105	Sulawesi	Female	Wallacea
AMNH	M-223106	Sulawesi	Female	Wallacea
AMNH	M-223108	Sulawesi	Female	Wallacea
AMNH	M-223112	Sulawesi	Female	Wallacea
AMNH	M-223113	Sulawesi	Female	Wallacea
AMNH	M-223116	Sulawesi	Female	Wallacea
AMNH	M-223117	Sulawesi	Female	Wallacea
AMNH	M-223118	Sulawesi	Female	Wallacea
AMNH	M-223119	Sulawesi	Female	Wallacea
AMNH	M-223121	Sulawesi	Female	Wallacea
AMNH	M-223122	Sulawesi	Female	Wallacea
AMNH	M-223126	Sulawesi	Female	Wallacea
AMNH	M-223128	Sulawesi	Female	Wallacea
AMNH	M-223131	Sulawesi	Female	Wallacea
AMNH	M-223133	Sulawesi	Female	Wallacea
AMNH	M-223135	Sulawesi	Female	Wallacea
AMNH	M-223136	Sulawesi	Female	Wallacea
AMNH	M-223137	Sulawesi	Female	Wallacea
AMNH	M-223141	Sulawesi	Female	Wallacea

Institute	Specimen	Island	Sex	Biogeographical region
AMNH	M-223144	Sulawesi	Female	Wallacea
AMNH	M-223146	Sulawesi	Female	Wallacea
AMNH	M-223367	Sulawesi	Female	Wallacea
AMNH	M-102672	Sumatra	Female	Greater Sunda Islands
AMNH	M-102675	Sumatra	Female	Greater Sunda Islands
AMNH	M-102676	Sumatra	Female	Greater Sunda Islands
AMNH	M-102677	Sumatra	Female	Greater Sunda Islands
AMNH	M-102678	Sumatra	Female	Greater Sunda Islands
AMNH	M-102679	Sumatra	Female	Greater Sunda Islands
AMNH	M-102682	Sumatra	Female	Greater Sunda Islands
AMNH	M-102684	Sumatra	Female	Greater Sunda Islands
AMNH	M-102690	Sumatra	Female	Greater Sunda Islands
AMNH	M-102802	Sumatra	Female	Greater Sunda Islands
AMNH	M-106360	Sumatra	Female	Greater Sunda Islands
AMNH	M-106361	Sumatra	Female	Greater Sunda Islands
AMNH	M-106362	Sumatra	Female	Greater Sunda Islands
AMNH	M-106363	Sumatra	Female	Greater Sunda Islands
AMNH	M-106364	Sumatra	Female	Greater Sunda Islands
AMNH	M-106367	Sumatra	Female	Greater Sunda Islands
AMNH	M-106368	Sumatra	Female	Greater Sunda Islands
AMNH	M-106369	Sumatra	Female	Greater Sunda Islands
AMNH	M-106370	Sumatra	Female	Greater Sunda Islands
AMNH	M-106371	Sumatra	Female	Greater Sunda Islands
AMNH	M-106659	Sumatra	Female	Greater Sunda Islands
AMNH	M-106660	Sumatra	Female	Greater Sunda Islands
AMNH	M-244145	Sumatra	Female	Greater Sunda Islands
AMNH	M-109300	Taliabu	Female	Wallacea
AMNH	M-109302	Taliabu	Female	Wallacea
AMNH	M-109303	Taliabu	Female	Wallacea
AMNH	M-109308	Taliabu	Female	Wallacea
AMNH	M-109312	Taliabu	Female	Wallacea
AMNH	M-109313	Taliabu	Female	Wallacea
AMNH	M-109315	Taliabu	Female	Wallacea
AMNH	M-109316	Taliabu	Female	Wallacea
AMNH	M-109317	Taliabu	Female	Wallacea
AMNH	M-159687	Woodlark Island	Female	Melanesia
AMNH	M-159688	Woodlark Island	Female	Melanesia
AMNH	M-159690	Woodlark Island	Female	Melanesia
AMNH	M-159691	Woodlark Island	Female	Melanesia
AMNH	M-267704	Ambon	Male	Wallacea
AMNH	M-102159	Bali	Male	Lesser Sunda Islands
AMNH	M-107979	Bali	Male	Lesser Sunda Islands
AMNH	M-107980	Bali	Male	Lesser Sunda Islands
AMNH	M-107983	Bali	Male	Lesser Sunda Islands
AMNH	M-107984	Bali	Male	Lesser Sunda Islands
AMNH	M-103516	Borneo	Male	Greater Sunda Islands
AMNH	M-103518	Borneo	Male	Greater Sunda Islands
AMNH	M-103519	Borneo	Male	Greater Sunda Islands
AMNH	M-103520	Borneo	Male	Greater Sunda Islands
AMNH	M-103521	Borneo	Male	Greater Sunda Islands
AMNH	M-103522	Borneo	Male	Greater Sunda Islands
AMNH	M-103527	Borneo	Male	Greater Sunda Islands
AMNH	M-103829	Borneo	Male	Greater Sunda Islands
AMNH	M-103830	Borneo	Male	Greater Sunda Islands
AMNH	M-103833	Borneo	Male	Greater Sunda Islands
AMNH	M-103841	Borneo	Male	Greater Sunda Islands
AMNH	M-103928	Borneo	Male	Greater Sunda Islands
AMNH	M-103929	Borneo	Male	Greater Sunda Islands
AMNH	M-103931	Borneo	Male	Greater Sunda Islands
AMNH	M-103932	Borneo	Male	Greater Sunda Islands
AMNH	M-103933	Borneo	Male	Greater Sunda Islands
AMNH	M-106127	Borneo	Male	Greater Sunda Islands
AMNH	M-106129	Borneo	Male	Greater Sunda Islands
AMNH	M-106194	Borneo	Male	Greater Sunda Islands
AMNH	M-106198	Borneo	Male	Greater Sunda Islands
AMNH	M-106201	Borneo	Male	Greater Sunda Islands

Institute	Specimen	Island	Sex	Biogeographical region
AMNH	M-106202	Borneo	Male	Greater Sunda Islands
AMNH	M-106203	Borneo	Male	Greater Sunda Islands
AMNH	M-106204	Borneo	Male	Greater Sunda Islands
AMNH	M-106205	Borneo	Male	Greater Sunda Islands
AMNH	M-106207	Borneo	Male	Greater Sunda Islands
AMNH	M-107028	Borneo	Male	Greater Sunda Islands
AMNH	M-107029	Borneo	Male	Greater Sunda Islands
AMNH	M-107030	Borneo	Male	Greater Sunda Islands
AMNH	M-107032	Borneo	Male	Greater Sunda Islands
AMNH	M-107033	Borneo	Male	Greater Sunda Islands
AMNH	M-107036	Borneo	Male	Greater Sunda Islands
AMNH	M-107038	Borneo	Male	Greater Sunda Islands
AMNH	M-107039	Borneo	Male	Greater Sunda Islands
AMNH	M-107046	Borneo	Male	Greater Sunda Islands
AMNH	M-163810	Burma	Male	Mainland
AMNH	M-163813	Burma	Male	Mainland
AMNH	M-163815	Burma	Male	Mainland
AMNH	M-163816	Burma	Male	Mainland
AMNH	M-163817	Burma	Male	Mainland
AMNH	M-31296	Buru Island	Male	Wallacea
AMNH	M-159737	Fergusson Island	Male	Melanesia
AMNH	M-159738	Fergusson Island	Male	Melanesia
AMNH	M-159739	Fergusson Island	Male	Melanesia
AMNH	M-159740	Fergusson Island	Male	Melanesia
AMNH	M-157925	Goodenough Island	Male	Melanesia
AMNH	M-157926	Goodenough Island	Male	Melanesia
AMNH	M-157927	Goodenough Island	Male	Melanesia
AMNH	M-157928	Goodenough Island	Male	Melanesia
AMNH	M-157929	Goodenough Island	Male	Melanesia
AMNH	M-157930	Goodenough Island	Male	Melanesia
AMNH	M-157931	Goodenough Island	Male	Melanesia
AMNH	M-157932	Goodenough Island	Male	Melanesia
AMNH	M-157933	Goodenough Island	Male	Melanesia
AMNH	M-157934	Goodenough Island	Male	Melanesia
AMNH	M-157942	Goodenough Island	Male	Melanesia
AMNH	M-157943	Goodenough Island	Male	Melanesia
AMNH	M-101295	Halmahera	Male	Wallacea
AMNH	M-101296	Halmahera	Male	Wallacea
AMNH	M-101298	Halmahera	Male	Wallacea
AMNH	M-101299	Halmahera	Male	Wallacea
AMNH	M-101300	Halmahera	Male	Wallacea
AMNH	M-101303	Halmahera	Male	Wallacea
AMNH	M-101304	Halmahera	Male	Wallacea
AMNH	M-267664	Halmahera	Male	Wallacea
AMNH	M-267665	Halmahera	Male	Wallacea
AMNH	M-267666	Halmahera	Male	Wallacea
AMNH	M-267667	Halmahera	Male	Wallacea
AMNH	M-267669	Halmahera	Male	Wallacea
AMNH	M-267670	Halmahera	Male	Wallacea
AMNH	M-267677	Halmahera	Male	Wallacea
AMNH	M-267679	Halmahera	Male	Wallacea
AMNH	M-267683	Halmahera	Male	Wallacea
AMNH	M-267686	Halmahera	Male	Wallacea
AMNH	M-267687	Halmahera	Male	Wallacea
AMNH	M-267688	Halmahera	Male	Wallacea
AMNH	M-267689	Halmahera	Male	Wallacea
AMNH	M-143880	Hawaii	Male	Polynesia
AMNH	M-143881	Hawaii	Male	Polynesia
AMNH	M-102006	Java	Male	Greater Sunda Islands
AMNH	M-102007	Java	Male	Greater Sunda Islands
AMNH	M-106644	Java	Male	Greater Sunda Islands
AMNH	M-106868	Java	Male	Greater Sunda Islands
AMNH	M-187190	Luzon	Male	Wallacea
AMNH	M-187191	Luzon	Male	Wallacea
AMNH	M-187192	Luzon	Male	Wallacea
AMNH	M-153296	Malenge Island	Male	Wallacea

Institute	Specimen	Island	Sex	Biogeographical region
AMNH	M-153297	Malenge Island	Male	Wallacea
AMNH	M-153298	Malenge Island	Male	Wallacea
AMNH	M-153299	Malenge Island	Male	Wallacea
AMNH	M-153300	Malenge Island	Male	Wallacea
AMNH	M-153302	Malenge Island	Male	Wallacea
AMNH	M-153304	Malenge Island	Male	Wallacea
AMNH	M-153307	Malenge Island	Male	Wallacea
AMNH	M-153308	Malenge Island	Male	Wallacea
AMNH	M-153309	Malenge Island	Male	Wallacea
AMNH	M-153312	Malenge Island	Male	Wallacea
AMNH	M-153313	Malenge Island	Male	Wallacea
AMNH	M-153315	Malenge Island	Male	Wallacea
AMNH	M-153316	Malenge Island	Male	Wallacea
AMNH	M-32659	Mindoro	Male	Wallacea
AMNH	M-159732	Misima Island	Male	Melanesia
AMNH	M-159736	Misima Island	Male	Melanesia
AMNH	M-207545	Negros	Male	Wallacea
AMNH	M-207546	Negros	Male	Wallacea
AMNH	M-207548	Negros	Male	Wallacea
AMNH	M-207549	Negros	Male	Wallacea
AMNH	M-207550	Negros	Male	Wallacea
AMNH	M-194381	New Britain Island	Male	Melanesia
AMNH	M-194382	New Britain Island	Male	Melanesia
AMNH	M-194386	New Britain Island	Male	Melanesia
AMNH	M-194387	New Britain Island	Male	Melanesia
AMNH	M-194389	New Britain Island	Male	Melanesia
AMNH	M-194391	New Britain Island	Male	Melanesia
AMNH	M-222323	New Britain Island	Male	Melanesia
AMNH	M-222324	New Britain Island	Male	Melanesia
AMNH	M-222328	New Britain Island	Male	Melanesia
AMNH	M-222329	New Britain Island	Male	Melanesia
AMNH	M-222331	New Britain Island	Male	Melanesia
AMNH	M-222334	New Britain Island	Male	Melanesia
AMNH	M-222335	New Britain Island	Male	Melanesia
AMNH	M-222336	New Britain Island	Male	Melanesia
AMNH	M-222401	New Britain Island	Male	Melanesia
AMNH	M-104161	New Guinea	Male	Melanesia
AMNH	M-104220	New Guinea	Male	Melanesia
AMNH	M-104236	New Guinea	Male	Melanesia
AMNH	M-104241	New Guinea	Male	Melanesia
AMNH	M-104250	New Guinea	Male	Melanesia
AMNH	M-104275	New Guinea	Male	Melanesia
AMNH	M-108201	New Guinea	Male	Melanesia
AMNH	M-108215	New Guinea	Male	Melanesia
AMNH	M-108247	New Guinea	Male	Melanesia
AMNH	M-108248	New Guinea	Male	Melanesia
AMNH	M-108249	New Guinea	Male	Melanesia
AMNH	M-108250	New Guinea	Male	Melanesia
AMNH	M-110085	New Guinea	Male	Melanesia
AMNH	M-110088	New Guinea	Male	Melanesia
AMNH	M-110093	New Guinea	Male	Melanesia
AMNH	M-110094	New Guinea	Male	Melanesia
AMNH	M-110095	New Guinea	Male	Melanesia
AMNH	M-110096	New Guinea	Male	Melanesia
AMNH	M-110097	New Guinea	Male	Melanesia
AMNH	M-110100	New Guinea	Male	Melanesia
AMNH	M-110101	New Guinea	Male	Melanesia
AMNH	M-110103	New Guinea	Male	Melanesia
AMNH	M-110107	New Guinea	Male	Melanesia
AMNH	M-110110	New Guinea	Male	Melanesia
AMNH	M-110111	New Guinea	Male	Melanesia
AMNH	M-110114	New Guinea	Male	Melanesia
AMNH	M-110121	New Guinea	Male	Melanesia
AMNH	M-110122	New Guinea	Male	Melanesia
AMNH	M-110125	New Guinea	Male	Melanesia
AMNH	M-110127	New Guinea	Male	Melanesia

Institute	Specimen	Island	Sex	Biogeographical region
AMNH	M-110129	New Guinea	Male	Melanesia
AMNH	M-110130	New Guinea	Male	Melanesia
AMNH	M-143870	New Guinea	Male	Melanesia
AMNH	M-143872	New Guinea	Male	Melanesia
AMNH	M-150919	New Guinea	Male	Melanesia
AMNH	M-157858l	New Guinea	Male	Melanesia
AMNH	M-157859	New Guinea	Male	Melanesia
AMNH	M-157860	New Guinea	Male	Melanesia
AMNH	M-157861	New Guinea	Male	Melanesia
AMNH	M-157862	New Guinea	Male	Melanesia
AMNH	M-157863	New Guinea	Male	Melanesia
AMNH	M-157864	New Guinea	Male	Melanesia
AMNH	M-157865	New Guinea	Male	Melanesia
AMNH	M-157866	New Guinea	Male	Melanesia
AMNH	M-157867	New Guinea	Male	Melanesia
AMNH	M-157868	New Guinea	Male	Melanesia
AMNH	M-157869	New Guinea	Male	Melanesia
AMNH	M-157870	New Guinea	Male	Melanesia
AMNH	M-157871	New Guinea	Male	Melanesia
AMNH	M-157872	New Guinea	Male	Melanesia
AMNH	M-157908	New Guinea	Male	Melanesia
AMNH	M-157909	New Guinea	Male	Melanesia
AMNH	M-157910	New Guinea	Male	Melanesia
AMNH	M-157911	New Guinea	Male	Melanesia
AMNH	M-157912	New Guinea	Male	Melanesia
AMNH	M-157913	New Guinea	Male	Melanesia
AMNH	M-157914	New Guinea	Male	Melanesia
AMNH	M-157916	New Guinea	Male	Melanesia
AMNH	M-191543	New Guinea	Male	Melanesia
AMNH	M-191544	New Guinea	Male	Melanesia
AMNH	M-191545	New Guinea	Male	Melanesia
AMNH	M-191546	New Guinea	Male	Melanesia
AMNH	M-191557	New Guinea	Male	Melanesia
AMNH	M-191558	New Guinea	Male	Melanesia
AMNH	M-191559	New Guinea	Male	Melanesia
AMNH	M-191560	New Guinea	Male	Melanesia
AMNH	M-191564	New Guinea	Male	Melanesia
AMNH	M-191569	New Guinea	Male	Melanesia
AMNH	M-191570	New Guinea	Male	Melanesia
AMNH	M-191571	New Guinea	Male	Melanesia
AMNH	M-191572	New Guinea	Male	Melanesia
AMNH	M-191578	New Guinea	Male	Melanesia
AMNH	M-191579	New Guinea	Male	Melanesia
AMNH	M-191580	New Guinea	Male	Melanesia
AMNH	M-191581	New Guinea	Male	Melanesia
AMNH	M-191582	New Guinea	Male	Melanesia
AMNH	M-191583	New Guinea	Male	Melanesia
AMNH	M-191584	New Guinea	Male	Melanesia
AMNH	M-191585	New Guinea	Male	Melanesia
AMNH	M-191586	New Guinea	Male	Melanesia
AMNH	M-191587	New Guinea	Male	Melanesia
AMNH	M-191588	New Guinea	Male	Melanesia
AMNH	M-191595	New Guinea	Male	Melanesia
AMNH	M-191596	New Guinea	Male	Melanesia
AMNH	M-191597	New Guinea	Male	Melanesia
AMNH	M-191599	New Guinea	Male	Melanesia
AMNH	M-191600	New Guinea	Male	Melanesia
AMNH	M-191601	New Guinea	Male	Melanesia
AMNH	M-191610	New Guinea	Male	Melanesia
AMNH	M-191611	New Guinea	Male	Melanesia
AMNH	M-191612	New Guinea	Male	Melanesia
AMNH	M-191613	New Guinea	Male	Melanesia
AMNH	M-191614	New Guinea	Male	Melanesia
AMNH	M-191619	New Guinea	Male	Melanesia
AMNH	M-191805	New Guinea	Male	Melanesia
AMNH	M-195041	New Guinea	Male	Melanesia

Institute	Specimen	Island	Sex	Biogeographical region
AMNH	M-195042	New Guinea	Male	Melanesia
AMNH	M-195043	New Guinea	Male	Melanesia
AMNH	M-195045	New Guinea	Male	Melanesia
AMNH	M-195046	New Guinea	Male	Melanesia
AMNH	M-195052	New Guinea	Male	Melanesia
AMNH	M-195055	New Guinea	Male	Melanesia
AMNH	M-195056	New Guinea	Male	Melanesia
AMNH	M-195057	New Guinea	Male	Melanesia
AMNH	M-195058	New Guinea	Male	Melanesia
AMNH	M-195061	New Guinea	Male	Melanesia
AMNH	M-195063	New Guinea	Male	Melanesia
AMNH	M-195065	New Guinea	Male	Melanesia
AMNH	M-195066	New Guinea	Male	Melanesia
AMNH	M-195071	New Guinea	Male	Melanesia
AMNH	M-195073	New Guinea	Male	Melanesia
AMNH	M-195076	New Guinea	Male	Melanesia
AMNH	M-195077	New Guinea	Male	Melanesia
AMNH	M-195078	New Guinea	Male	Melanesia
AMNH	M-195080	New Guinea	Male	Melanesia
AMNH	M-195083	New Guinea	Male	Melanesia
AMNH	M-195084	New Guinea	Male	Melanesia
AMNH	M-195085	New Guinea	Male	Melanesia
AMNH	M-195086	New Guinea	Male	Melanesia
AMNH	M-195087	New Guinea	Male	Melanesia
AMNH	M-195090	New Guinea	Male	Melanesia
AMNH	M-198794	New Guinea	Male	Melanesia
AMNH	M-198795	New Guinea	Male	Melanesia
AMNH	M-198796	New Guinea	Male	Melanesia
AMNH	M-198797	New Guinea	Male	Melanesia
AMNH	M-198802	New Guinea	Male	Melanesia
AMNH	M-198803	New Guinea	Male	Melanesia
AMNH	M-198804	New Guinea	Male	Melanesia
AMNH	M-198805	New Guinea	Male	Melanesia
AMNH	M-198806	New Guinea	Male	Melanesia
AMNH	M-222286	New Guinea	Male	Melanesia
AMNH	M-222290	New Guinea	Male	Melanesia
AMNH	M-222293	New Guinea	Male	Melanesia
AMNH	M-222294	New Guinea	Male	Melanesia
AMNH	M-222295	New Guinea	Male	Melanesia
AMNH	M-222297	New Guinea	Male	Melanesia
AMNH	M-222299	New Guinea	Male	Melanesia
AMNH	M-222303	New Guinea	Male	Melanesia
AMNH	M-222304	New Guinea	Male	Melanesia
AMNH	M-222308	New Guinea	Male	Melanesia
AMNH	M-222309	New Guinea	Male	Melanesia
AMNH	M-222311	New Guinea	Male	Melanesia
AMNH	M-222312	New Guinea	Male	Melanesia
AMNH	M-222313	New Guinea	Male	Melanesia
AMNH	M-222314	New Guinea	Male	Melanesia
AMNH	M-222316	New Guinea	Male	Melanesia
AMNH	M-222318	New Guinea	Male	Melanesia
AMNH	M-222320	New Guinea	Male	Melanesia
AMNH	M-222339	New Guinea	Male	Melanesia
AMNH	M-222340	New Guinea	Male	Melanesia
AMNH	M-222341	New Guinea	Male	Melanesia
AMNH	M-222344	New Guinea	Male	Melanesia
AMNH	M-222346	New Guinea	Male	Melanesia
AMNH	M-222350	New Guinea	Male	Melanesia
AMNH	M-222353	New Guinea	Male	Melanesia
AMNH	M-222354	New Guinea	Male	Melanesia
AMNH	M-222356	New Guinea	Male	Melanesia
AMNH	M-222361	New Guinea	Male	Melanesia
AMNH	M-222363	New Guinea	Male	Melanesia
AMNH	M-222364	New Guinea	Male	Melanesia
AMNH	M-222365	New Guinea	Male	Melanesia
AMNH	M-222384	New Guinea	Male	Melanesia

Institute	Specimen	Island	Sex	Biogeographical region
AMNH	M-222386	New Guinea	Male	Melanesia
AMNH	M-222388	New Guinea	Male	Melanesia
AMNH	M-222392	New Guinea	Male	Melanesia
AMNH	M-222394	New Guinea	Male	Melanesia
AMNH	M-222396	New Guinea	Male	Melanesia
AMNH	M-222404	New Guinea	Male	Melanesia
AMNH	M-159744	Normanby Island	Male	Melanesia
AMNH	M-159745	Normanby Island	Male	Melanesia
AMNH	M-159746	Normanby Island	Male	Melanesia
AMNH	M-159747	Normanby Island	Male	Melanesia
AMNH	M-159748	Normanby Island	Male	Melanesia
AMNH	M-159749	Normanby Island	Male	Melanesia
AMNH	M-159750	Normanby Island	Male	Melanesia
AMNH	M-159751	Normanby Island	Male	Melanesia
AMNH	M-159752	Normanby Island	Male	Melanesia
AMNH	M-159753	Normanby Island	Male	Melanesia
AMNH	M-159754	Normanby Island	Male	Melanesia
AMNH	M-159755	Normanby Island	Male	Melanesia
AMNH	M-159756	Normanby Island	Male	Melanesia
AMNH	M-159757	Normanby Island	Male	Melanesia
AMNH	M-207607	Palawan	Male	Sunda
AMNH	M-109176	Peleng Island	Male	Wallacea
AMNH	M-109177	Peleng Island	Male	Wallacea
AMNH	M-109178	Peleng Island	Male	Wallacea
AMNH	M-109180	Peleng Island	Male	Wallacea
AMNH	M-68742	Rose Island	Male	Polynesia
AMNH	M-159692	Rossel Island	Male	Melanesia
AMNH	M-159693	Rossel Island	Male	Melanesia
AMNH	M-159694	Rossel Island	Male	Melanesia
AMNH	M-159695	Rossel Island	Male	Melanesia
AMNH	M-159696	Rossel Island	Male	Melanesia
AMNH	M-159697	Rossel Island	Male	Melanesia
AMNH	M-159698	Rossel Island	Male	Melanesia
AMNH	M-159699	Rossel Island	Male	Melanesia
AMNH	M-159712	Rossel Island	Male	Melanesia
AMNH	M-159713	Rossel Island	Male	Melanesia
AMNH	M-159717	Sudest Island	Male	Melanesia
AMNH	M-159718	Sudest Island	Male	Melanesia
AMNH	M-159719	Sudest Island	Male	Melanesia
AMNH	M-159720	Sudest Island	Male	Melanesia
AMNH	M-159721	Sudest Island	Male	Melanesia
AMNH	M-159722	Sudest Island	Male	Melanesia
AMNH	M-159723	Sudest Island	Male	Melanesia
AMNH	M-159724	Sudest Island	Male	Melanesia
AMNH	M-159729	Sudest Island	Male	Melanesia
AMNH	M-103165	Sulawesi	Male	Greater Sunda Islands
AMNH	M-100985	Sulawesi	Male	Wallacea
AMNH	M-100987	Sulawesi	Male	Wallacea
AMNH	M-100989	Sulawesi	Male	Wallacea
AMNH	M-100990	Sulawesi	Male	Wallacea
AMNH	M-100992	Sulawesi	Male	Wallacea
AMNH	M-101047	Sulawesi	Male	Wallacea
AMNH	M-101048	Sulawesi	Male	Wallacea
AMNH	M-101049	Sulawesi	Male	Wallacea
AMNH	M-101190	Sulawesi	Male	Wallacea
AMNH	M-101262	Sulawesi	Male	Wallacea
AMNH	M-101264	Sulawesi	Male	Wallacea
AMNH	M-101265	Sulawesi	Male	Wallacea
AMNH	M-101270	Sulawesi	Male	Wallacea
AMNH	M-153014	Sulawesi	Male	Wallacea
AMNH	M-153022	Sulawesi	Male	Wallacea
AMNH	M-153023	Sulawesi	Male	Wallacea
AMNH	M-153024	Sulawesi	Male	Wallacea
AMNH	M-153025	Sulawesi	Male	Wallacea
AMNH	M-153026	Sulawesi	Male	Wallacea
AMNH	M-153028	Sulawesi	Male	Wallacea

Institute	Specimen	Island	Sex	Biogeographical region
AMNH	M-153031	Sulawesi	Male	Wallacea
AMNH	M-153035	Sulawesi	Male	Wallacea
AMNH	M-153036	Sulawesi	Male	Wallacea
AMNH	M-223101	Sulawesi	Male	Wallacea
AMNH	M-223103	Sulawesi	Male	Wallacea
AMNH	M-223107	Sulawesi	Male	Wallacea
AMNH	M-223109	Sulawesi	Male	Wallacea
AMNH	M-223110	Sulawesi	Male	Wallacea
AMNH	M-223111	Sulawesi	Male	Wallacea
AMNH	M-223114	Sulawesi	Male	Wallacea
AMNH	M-223115	Sulawesi	Male	Wallacea
AMNH	M-223120	Sulawesi	Male	Wallacea
AMNH	M-223123	Sulawesi	Male	Wallacea
AMNH	M-223124	Sulawesi	Male	Wallacea
AMNH	M-223125	Sulawesi	Male	Wallacea
AMNH	M-223127	Sulawesi	Male	Wallacea
AMNH	M-223129	Sulawesi	Male	Wallacea
AMNH	M-223130	Sulawesi	Male	Wallacea
AMNH	M-223132	Sulawesi	Male	Wallacea
AMNH	M-223134	Sulawesi	Male	Wallacea
AMNH	M-223138	Sulawesi	Male	Wallacea
AMNH	M-223139	Sulawesi	Male	Wallacea
AMNH	M-223140	Sulawesi	Male	Wallacea
AMNH	M-223142	Sulawesi	Male	Wallacea
AMNH	M-223143	Sulawesi	Male	Wallacea
AMNH	M-223145	Sulawesi	Male	Wallacea
AMNH	M-223471	Sulawesi	Male	Wallacea
AMNH	M-224182	Sulawesi	Male	Wallacea
AMNH	M-267775	Sulawesi	Male	Wallacea
AMNH	M-267776	Sulawesi	Male	Wallacea
AMNH	M-102656	Sumatra	Male	Greater Sunda Islands
AMNH	M-102669	Sumatra	Male	Greater Sunda Islands
AMNH	M-102670	Sumatra	Male	Greater Sunda Islands
AMNH	M-102671	Sumatra	Male	Greater Sunda Islands
AMNH	M-102680	Sumatra	Male	Greater Sunda Islands
AMNH	M-102683	Sumatra	Male	Greater Sunda Islands
AMNH	M-102685	Sumatra	Male	Greater Sunda Islands
AMNH	M-102687	Sumatra	Male	Greater Sunda Islands
AMNH	M-102688	Sumatra	Male	Greater Sunda Islands
AMNH	M-102689	Sumatra	Male	Greater Sunda Islands
AMNH	M-102799	Sumatra	Male	Greater Sunda Islands
AMNH	M-102800	Sumatra	Male	Greater Sunda Islands
AMNH	M-102801	Sumatra	Male	Greater Sunda Islands
AMNH	M-102803	Sumatra	Male	Greater Sunda Islands
AMNH	M-102997	Sumatra	Male	Greater Sunda Islands
AMNH	M-106365	Sumatra	Male	Greater Sunda Islands
AMNH	M-106366	Sumatra	Male	Greater Sunda Islands
AMNH	M-106372	Sumatra	Male	Greater Sunda Islands
AMNH	M-109301	Taliabu	Male	Wallacea
AMNH	M-109304	Taliabu	Male	Wallacea
AMNH	M-109305	Taliabu	Male	Wallacea
AMNH	M-109306	Taliabu	Male	Wallacea
AMNH	M-109307	Taliabu	Male	Wallacea
AMNH	M-109309	Taliabu	Male	Wallacea
AMNH	M-109310	Taliabu	Male	Wallacea
AMNH	M-109311	Taliabu	Male	Wallacea
AMNH	M-109314	Taliabu	Male	Wallacea
AMNH	M-68744	Tau Island	Male	Polynesia
AMNH	M-240756	Vietnam	Male	Mainland
AMNH	M-159684	Woodlark Island	Male	Melanesia
AMNH	M-159685	Woodlark Island	Male	Melanesia
AMNH	M-159686	Woodlark Island	Male	Melanesia
AMNH	M-103842	Borneo	Unknown	Greater Sunda Islands
AMNH	M-103843	Borneo	Unknown	Greater Sunda Islands
AMNH	M-101302	Halmahera	Unknown	Wallacea
AMNH	M-101305	Halmahera	Unknown	Wallacea

Institute	Specimen	Island	Sex	Biogeographical region
AMNH	M-242148	Luzon	Unknown	Wallacea
AMNH	M-242182	Mindanao	Unknown	Wallacea
AMNH	M-222337	New Britain Island	Unknown	Melanesia
AMNH	M-108182	New Guinea	Unknown	Melanesia
AMNH	M-191608	New Guinea	Unknown	Melanesia
AMNH	M-191609	New Guinea	Unknown	Melanesia
AMNH	M-222302	New Guinea	Unknown	Melanesia
AMNH	M-222306	New Guinea	Unknown	Melanesia
AMNH	M-222367	New Guinea	Unknown	Melanesia
AMNH	M-222368	New Guinea	Unknown	Melanesia
AMNH	M-222369	New Guinea	Unknown	Melanesia
AMNH	M-222370	New Guinea	Unknown	Melanesia
AMNH	M-222371	New Guinea	Unknown	Melanesia
AMNH	M-222372	New Guinea	Unknown	Melanesia
AMNH	M-222373	New Guinea	Unknown	Melanesia
AMNH	M-222374	New Guinea	Unknown	Melanesia
AMNH	M-222375	New Guinea	Unknown	Melanesia
AMNH	M-222376	New Guinea	Unknown	Melanesia
AMNH	M-222377	New Guinea	Unknown	Melanesia
AMNH	M-222378	New Guinea	Unknown	Melanesia
AMNH	M-222379	New Guinea	Unknown	Melanesia
AMNH	M-222380	New Guinea	Unknown	Melanesia
AMNH	M-222381	New Guinea	Unknown	Melanesia
AMNH	M-222383	New Guinea	Unknown	Melanesia
AMNH	M-222385	New Guinea	Unknown	Melanesia
AMNH	M-222387	New Guinea	Unknown	Melanesia
AMNH	M-222389	New Guinea	Unknown	Melanesia
AMNH	M-222391	New Guinea	Unknown	Melanesia
AMNH	M-222393	New Guinea	Unknown	Melanesia
AMNH	M-222395	New Guinea	Unknown	Melanesia
AMNH	M-222397	New Guinea	Unknown	Melanesia
AMNH	M-222398	New Guinea	Unknown	Melanesia
AMNH	M-222399	New Guinea	Unknown	Melanesia
AMNH	M-242207	Palawan	Unknown	Sunda
AMNH	M-242186	Polillo Island	Unknown	Wallacea
AMNH	M-109299	Taliabu	Unknown	Wallacea

Institute	Specimen	Island	Sex	Biogeographical region
USNM	292713	Borneo	Female	Greater Sunda Islands
USNM	292714	Borneo	Female	Greater Sunda Islands
USNM	292715	Borneo	Female	Greater Sunda Islands
USNM	292716	Borneo	Female	Greater Sunda Islands
USNM	292717	Borneo	Female	Greater Sunda Islands
USNM	292718	Borneo	Female	Greater Sunda Islands
USNM	292721	Borneo	Female	Greater Sunda Islands
USNM	292722	Borneo	Female	Greater Sunda Islands
USNM	292725	Borneo	Female	Greater Sunda Islands
USNM	292726	Borneo	Female	Greater Sunda Islands
USNM	292727	Borneo	Female	Greater Sunda Islands
USNM	292731	Borneo	Female	Greater Sunda Islands
USNM	292732	Borneo	Female	Greater Sunda Islands
USNM	301042	Borneo	Female	Greater Sunda Islands
USNM	317243	Borneo	Female	Greater Sunda Islands
USNM	197327	Borneo	Female	Greater Sunda Islands
USNM	197328	Borneo	Female	Greater Sunda Islands
USNM	197333	Borneo	Female	Greater Sunda Islands
USNM	197334	Borneo	Female	Greater Sunda Islands
USNM	197335	Borneo	Female	Greater Sunda Islands
USNM	197336	Borneo	Female	Greater Sunda Islands
USNM	197338	Borneo	Female	Greater Sunda Islands
USNM	197340	Borneo	Female	Greater Sunda Islands
USNM	197341	Borneo	Female	Greater Sunda Islands
USNM	142253	Borneo	Female	Greater Sunda Islands
USNM	142254	Borneo	Female	Greater Sunda Islands
USNM	142257	Borneo	Female	Greater Sunda Islands
USNM	145556	Borneo	Female	Greater Sunda Islands
USNM	145557	Borneo	Female	Greater Sunda Islands
USNM	145559	Borneo	Female	Greater Sunda Islands
USNM	145561	Borneo	Female	Greater Sunda Islands
USNM	153720	Borneo	Female	Greater Sunda Islands
USNM	198799	Borneo	Female	Greater Sunda Islands
USNM	197355	Borneo	Female	Greater Sunda Islands
USNM	489012	Borneo	Female	Greater Sunda Islands
USNM	489013	Borneo	Female	Greater Sunda Islands
USNM	521879	Borneo	Female	Greater Sunda Islands
USNM	521880	Borneo	Female	Greater Sunda Islands
USNM	521882	Borneo	Female	Greater Sunda Islands
USNM	521883	Borneo	Female	Greater Sunda Islands
USNM	521884	Borneo	Female	Greater Sunda Islands
USNM	301043	Borneo	Male	Greater Sunda Islands
USNM	521878	Borneo	Male	Greater Sunda Islands
USNM	292719	Borneo	Male	Greater Sunda Islands
USNM	292720	Borneo	Male	Greater Sunda Islands
USNM	292724	Borneo	Male	Greater Sunda Islands
USNM	292728	Borneo	Male	Greater Sunda Islands
USNM	292729	Borneo	Male	Greater Sunda Islands
USNM	292730	Borneo	Male	Greater Sunda Islands
USNM	301040	Borneo	Male	Greater Sunda Islands
USNM	301041	Borneo	Male	Greater Sunda Islands
USNM	301044	Borneo	Male	Greater Sunda Islands
USNM	317242	Borneo	Male	Greater Sunda Islands
USNM	317245	Borneo	Male	Greater Sunda Islands
USNM	197325	Borneo	Male	Greater Sunda Islands
USNM	197326	Borneo	Male	Greater Sunda Islands
USNM	197329	Borneo	Male	Greater Sunda Islands
USNM	197330	Borneo	Male	Greater Sunda Islands
USNM	197332	Borneo	Male	Greater Sunda Islands
USNM	197337	Borneo	Male	Greater Sunda Islands
USNM	197339	Borneo	Male	Greater Sunda Islands
USNM	197342	Borneo	Male	Greater Sunda Islands
USNM	197343	Borneo	Male	Greater Sunda Islands
USNM	197344	Borneo	Male	Greater Sunda Islands
USNM	197345	Borneo	Male	Greater Sunda Islands
USNM	197346	Borneo	Male	Greater Sunda Islands

Institute	Specimen	Island	Sex	Biogeographical region
USNM	197347	Borneo	Male	Greater Sunda Islands
USNM	142249	Borneo	Male	Greater Sunda Islands
USNM	145558	Borneo	Male	Greater Sunda Islands
USNM	145562	Borneo	Male	Greater Sunda Islands
USNM	145564	Borneo	Male	Greater Sunda Islands
USNM	197348	Borneo	Male	Greater Sunda Islands
USNM	197349	Borneo	Male	Greater Sunda Islands
USNM	196765	Borneo	Male	Greater Sunda Islands
USNM	196766	Borneo	Male	Greater Sunda Islands
USNM	521881	Borneo	Male	Greater Sunda Islands
USNM	197331	Borneo	Male	Greater Sunda Islands
USNM	317244	Borneo	Unknown	Greater Sunda Islands
USNM	155710	Java	Female	Greater Sunda Islands
USNM	481583	Java	Female	Greater Sunda Islands
USNM	359656	Java	Female	Greater Sunda Islands
USNM	359657	Java	Female	Greater Sunda Islands
USNM	359658	Java	Female	Greater Sunda Islands
USNM	359665	Java	Female	Greater Sunda Islands
USNM	481534	Java	Female	Greater Sunda Islands
USNM	481540	Java	Female	Greater Sunda Islands
USNM	481542	Java	Female	Greater Sunda Islands
USNM	481544	Java	Female	Greater Sunda Islands
USNM	481548	Java	Female	Greater Sunda Islands
USNM	481549	Java	Female	Greater Sunda Islands
USNM	481551	Java	Female	Greater Sunda Islands
USNM	481559	Java	Female	Greater Sunda Islands
USNM	481562	Java	Female	Greater Sunda Islands
USNM	481563	Java	Female	Greater Sunda Islands
USNM	481569	Java	Female	Greater Sunda Islands
USNM	481570	Java	Female	Greater Sunda Islands
USNM	481571	Java	Female	Greater Sunda Islands
USNM	481572	Java	Female	Greater Sunda Islands
USNM	481576	Java	Female	Greater Sunda Islands
USNM	481578	Java	Female	Greater Sunda Islands
USNM	481581	Java	Female	Greater Sunda Islands
USNM	481585	Java	Female	Greater Sunda Islands
USNM	481586	Java	Female	Greater Sunda Islands
USNM	481590	Java	Female	Greater Sunda Islands
USNM	481595	Java	Female	Greater Sunda Islands
USNM	496965	Java	Female	Greater Sunda Islands
USNM	521872	Java	Female	Greater Sunda Islands
USNM	521874	Java	Female	Greater Sunda Islands
USNM	521892	Java	Female	Greater Sunda Islands
USNM	521894	Java	Female	Greater Sunda Islands
USNM	521896	Java	Female	Greater Sunda Islands
USNM	521898	Java	Female	Greater Sunda Islands
USNM	521899	Java	Female	Greater Sunda Islands
USNM	521900	Java	Female	Greater Sunda Islands
USNM	521903	Java	Female	Greater Sunda Islands
USNM	521911	Java	Female	Greater Sunda Islands
USNM	521913	Java	Female	Greater Sunda Islands
USNM	521914	Java	Female	Greater Sunda Islands
USNM	535941	Java	Female	Greater Sunda Islands
USNM	535944	Java	Female	Greater Sunda Islands
USNM	535946	Java	Female	Greater Sunda Islands
USNM	535947	Java	Female	Greater Sunda Islands
USNM	535948	Java	Female	Greater Sunda Islands
USNM	535949	Java	Female	Greater Sunda Islands
USNM	535951	Java	Female	Greater Sunda Islands
USNM	535959	Java	Female	Greater Sunda Islands
USNM	481566	Java	Female	Greater Sunda Islands
USNM	481567	Java	Female	Greater Sunda Islands
USNM	155711	Java	Female	Greater Sunda Islands
USNM	481587	Java	Male	Greater Sunda Islands
USNM	155709	Java	Male	Greater Sunda Islands
USNM	155713	Java	Male	Greater Sunda Islands

Institute	Specimen	Island	Sex	Biogeographical region
USNM	156383	Java	Male	Greater Sunda Islands
USNM	359659	Java	Male	Greater Sunda Islands
USNM	359660	Java	Male	Greater Sunda Islands
USNM	359661	Java	Male	Greater Sunda Islands
USNM	359662	Java	Male	Greater Sunda Islands
USNM	359663	Java	Male	Greater Sunda Islands
USNM	359664	Java	Male	Greater Sunda Islands
USNM	481529	Java	Male	Greater Sunda Islands
USNM	481530	Java	Male	Greater Sunda Islands
USNM	481531	Java	Male	Greater Sunda Islands
USNM	481532	Java	Male	Greater Sunda Islands
USNM	481533	Java	Male	Greater Sunda Islands
USNM	481535	Java	Male	Greater Sunda Islands
USNM	481536	Java	Male	Greater Sunda Islands
USNM	481537	Java	Male	Greater Sunda Islands
USNM	481538	Java	Male	Greater Sunda Islands
USNM	481539	Java	Male	Greater Sunda Islands
USNM	481541	Java	Male	Greater Sunda Islands
USNM	481543	Java	Male	Greater Sunda Islands
USNM	481545	Java	Male	Greater Sunda Islands
USNM	481550	Java	Male	Greater Sunda Islands
USNM	481552	Java	Male	Greater Sunda Islands
USNM	481553	Java	Male	Greater Sunda Islands
USNM	481554	Java	Male	Greater Sunda Islands
USNM	481555	Java	Male	Greater Sunda Islands
USNM	481556	Java	Male	Greater Sunda Islands
USNM	481557	Java	Male	Greater Sunda Islands
USNM	481560	Java	Male	Greater Sunda Islands
USNM	481561	Java	Male	Greater Sunda Islands
USNM	481564	Java	Male	Greater Sunda Islands
USNM	481575	Java	Male	Greater Sunda Islands
USNM	481577	Java	Male	Greater Sunda Islands
USNM	481579	Java	Male	Greater Sunda Islands
USNM	481580	Java	Male	Greater Sunda Islands
USNM	481588	Java	Male	Greater Sunda Islands
USNM	481589	Java	Male	Greater Sunda Islands
USNM	481591	Java	Male	Greater Sunda Islands
USNM	481593	Java	Male	Greater Sunda Islands
USNM	481594	Java	Male	Greater Sunda Islands
USNM	496951	Java	Male	Greater Sunda Islands
USNM	496963	Java	Male	Greater Sunda Islands
USNM	496966	Java	Male	Greater Sunda Islands
USNM	496967	Java	Male	Greater Sunda Islands
USNM	521873	Java	Male	Greater Sunda Islands
USNM	521875	Java	Male	Greater Sunda Islands
USNM	521876	Java	Male	Greater Sunda Islands
USNM	521877	Java	Male	Greater Sunda Islands
USNM	521885	Java	Male	Greater Sunda Islands
USNM	521891	Java	Male	Greater Sunda Islands
USNM	521893	Java	Male	Greater Sunda Islands
USNM	521895	Java	Male	Greater Sunda Islands
USNM	521897	Java	Male	Greater Sunda Islands
USNM	521902	Java	Male	Greater Sunda Islands
USNM	521908	Java	Male	Greater Sunda Islands
USNM	521909	Java	Male	Greater Sunda Islands
USNM	521910	Java	Male	Greater Sunda Islands
USNM	521912	Java	Male	Greater Sunda Islands
USNM	521915	Java	Male	Greater Sunda Islands
USNM	521916	Java	Male	Greater Sunda Islands
USNM	535942	Java	Male	Greater Sunda Islands
USNM	535943	Java	Male	Greater Sunda Islands
USNM	535945	Java	Male	Greater Sunda Islands
USNM	535950	Java	Male	Greater Sunda Islands
USNM	535952	Java	Male	Greater Sunda Islands
USNM	535953	Java	Male	Greater Sunda Islands
USNM	535954	Java	Male	Greater Sunda Islands

Institute	Specimen	Island	Sex	Biogeographical region
USNM	535955	Java	Male	Greater Sunda Islands
USNM	535957	Java	Male	Greater Sunda Islands
USNM	535958	Java	Male	Greater Sunda Islands
USNM	535960	Java	Male	Greater Sunda Islands
USNM	481558	Java	Male	Greater Sunda Islands
USNM	481565	Java	Male	Greater Sunda Islands
USNM	481568	Java	Male	Greater Sunda Islands
USNM	481573	Java	Male	Greater Sunda Islands
USNM	481574	Java	Male	Greater Sunda Islands
USNM	481582	Java	Male	Greater Sunda Islands
USNM	481584	Java	Male	Greater Sunda Islands
USNM	481592	Java	Male	Greater Sunda Islands
USNM	521886	Java	Male	Greater Sunda Islands
USNM	521901	Java	Male	Greater Sunda Islands
USNM	535956	Java	Male	Greater Sunda Islands
USNM	141196	Sumatra	Female	Greater Sunda Islands
USNM	141198	Sumatra	Female	Greater Sunda Islands
USNM	141199	Sumatra	Female	Greater Sunda Islands
USNM	141200	Sumatra	Female	Greater Sunda Islands
USNM	142126	Sumatra	Female	Greater Sunda Islands
USNM	142127	Sumatra	Female	Greater Sunda Islands
USNM	143249	Sumatra	Female	Greater Sunda Islands
USNM	143251	Sumatra	Female	Greater Sunda Islands
USNM	114188	Sumatra	Female	Greater Sunda Islands
USNM	114189	Sumatra	Female	Greater Sunda Islands
USNM	114195	Sumatra	Female	Greater Sunda Islands
USNM	114197	Sumatra	Female	Greater Sunda Islands
USNM	114199	Sumatra	Female	Greater Sunda Islands
USNM	124888	Sumatra	Female	Greater Sunda Islands
USNM	141066	Sumatra	Male	Greater Sunda Islands
USNM	141068	Sumatra	Male	Greater Sunda Islands
USNM	144233	Sumatra	Male	Greater Sunda Islands
USNM	141197	Sumatra	Male	Greater Sunda Islands
USNM	114181	Sumatra	Male	Greater Sunda Islands
USNM	114182	Sumatra	Male	Greater Sunda Islands
USNM	114183	Sumatra	Male	Greater Sunda Islands
USNM	114185	Sumatra	Male	Greater Sunda Islands
USNM	114186	Sumatra	Male	Greater Sunda Islands
USNM	114187	Sumatra	Male	Greater Sunda Islands
USNM	114190	Sumatra	Male	Greater Sunda Islands
USNM	114191	Sumatra	Male	Greater Sunda Islands
USNM	114192	Sumatra	Male	Greater Sunda Islands
USNM	114193	Sumatra	Male	Greater Sunda Islands
USNM	114194	Sumatra	Male	Greater Sunda Islands
USNM	114196	Sumatra	Male	Greater Sunda Islands
USNM	114198	Sumatra	Male	Greater Sunda Islands
USNM	114200	Sumatra	Male	Greater Sunda Islands
USNM	121506	Sumatra	Male	Greater Sunda Islands
USNM	121507	Sumatra	Male	Greater Sunda Islands
USNM	114184	Sumatra	Male	Greater Sunda Islands
USNM	521904	Sumatra	Male	Greater Sunda Islands
USNM	521905	Sumatra	Male	Greater Sunda Islands
USNM	521906	Sumatra	Male	Greater Sunda Islands
USNM	521907	Sumatra	Male	Greater Sunda Islands
USNM	530281	Bangladesh	Female	Mainland
USNM	530282	Bangladesh	Female	Mainland
USNM	240536	Laos	Female	Mainland
USNM	240541	Laos	Female	Mainland
USNM	240542	Laos	Female	Mainland
USNM	355472	Laos	Male	Mainland
USNM	240540	Laos	Male	Mainland
USNM	143295	Malaysia	Female	Mainland
USNM	355370	Malaysia	Female	Mainland
USNM	112443	Malaysia	Female	Mainland
USNM	290237	Malaysia	Female	Mainland
USNM	152190	Malaysia	Female	Mainland

Institute	Specimen	Island	Sex	Biogeographical region
USNM	291293	Malaysia	Female	Mainland
USNM	101764	Malaysia	Male	Mainland
USNM	152189	Malaysia	Male	Mainland
USNM	283696	Malaysia	Male	Mainland
USNM	291294	Malaysia	Male	Mainland
USNM	355369	Malaysia	Male	Mainland
USNM	277426	Myanmar	Female	Mainland
USNM	277812	Myanmar	Female	Mainland
USNM	277813	Myanmar	Female	Mainland
USNM	277814	Myanmar	Female	Mainland
USNM	277815	Myanmar	Female	Mainland
USNM	277818	Myanmar	Female	Mainland
USNM	277821	Myanmar	Female	Mainland
USNM	277822	Myanmar	Female	Mainland
USNM	277823	Myanmar	Female	Mainland
USNM	277824	Myanmar	Female	Mainland
USNM	277825	Myanmar	Female	Mainland
USNM	277827	Myanmar	Female	Mainland
USNM	277866	Myanmar	Female	Mainland
USNM	279287	Myanmar	Female	Mainland
USNM	279288	Myanmar	Female	Mainland
USNM	279289	Myanmar	Female	Mainland
USNM	279292	Myanmar	Female	Mainland
USNM	296805	Myanmar	Female	Mainland
USNM	583804	Myanmar	Female	Mainland
USNM	277425	Myanmar	Male	Mainland
USNM	277811	Myanmar	Male	Mainland
USNM	277816	Myanmar	Male	Mainland
USNM	277817	Myanmar	Male	Mainland
USNM	277819	Myanmar	Male	Mainland
USNM	277820	Myanmar	Male	Mainland
USNM	277826	Myanmar	Male	Mainland
USNM	277828	Myanmar	Male	Mainland
USNM	277829	Myanmar	Male	Mainland
USNM	277865	Myanmar	Male	Mainland
USNM	277867	Myanmar	Male	Mainland
USNM	279290	Myanmar	Male	Mainland
USNM	279291	Myanmar	Male	Mainland
USNM	111981	Myanmar	Male	Mainland
USNM	583803	Myanmar	Male	Mainland
USNM	583805	Myanmar	Male	Mainland
USNM	294352	Thailand	Female	Mainland
USNM	294354	Thailand	Female	Mainland
USNM	294394	Thailand	Female	Mainland
USNM	355154	Thailand	Female	Mainland
USNM	355161	Thailand	Female	Mainland
USNM	355175	Thailand	Female	Mainland
USNM	355176	Thailand	Female	Mainland
USNM	221539	Thailand	Female	Mainland
USNM	221540	Thailand	Female	Mainland
USNM	241082	Thailand	Female	Mainland
USNM	241460	Thailand	Female	Mainland
USNM	253595	Thailand	Female	Mainland
USNM	253596	Thailand	Female	Mainland
USNM	253597	Thailand	Female	Mainland
USNM	257821	Thailand	Female	Mainland
USNM	260637	Thailand	Female	Mainland
USNM	294346	Thailand	Female	Mainland
USNM	294347	Thailand	Female	Mainland
USNM	294348	Thailand	Female	Mainland
USNM	294350	Thailand	Female	Mainland
USNM	294389	Thailand	Female	Mainland
USNM	294390	Thailand	Female	Mainland
USNM	294391	Thailand	Female	Mainland
USNM	294392	Thailand	Female	Mainland
USNM	294396	Thailand	Female	Mainland

Institute	Specimen	Island	Sex	Biogeographical region
USNM	294399	Thailand	Female	Mainland
USNM	294400	Thailand	Female	Mainland
USNM	294402	Thailand	Female	Mainland
USNM	294915	Thailand	Female	Mainland
USNM	294916	Thailand	Female	Mainland
USNM	294919	Thailand	Female	Mainland
USNM	294920	Thailand	Female	Mainland
USNM	294925	Thailand	Female	Mainland
USNM	294928	Thailand	Female	Mainland
USNM	294929	Thailand	Female	Mainland
USNM	296554	Thailand	Female	Mainland
USNM	296555	Thailand	Female	Mainland
USNM	296557	Thailand	Female	Mainland
USNM	297110	Thailand	Female	Mainland
USNM	297111	Thailand	Female	Mainland
USNM	297115	Thailand	Female	Mainland
USNM	297116	Thailand	Female	Mainland
USNM	297117	Thailand	Female	Mainland
USNM	297118	Thailand	Female	Mainland
USNM	297119	Thailand	Female	Mainland
USNM	297121	Thailand	Female	Mainland
USNM	297122	Thailand	Female	Mainland
USNM	297124	Thailand	Female	Mainland
USNM	297126	Thailand	Female	Mainland
USNM	297127	Thailand	Female	Mainland
USNM	297130	Thailand	Female	Mainland
USNM	297131	Thailand	Female	Mainland
USNM	297135	Thailand	Female	Mainland
USNM	308201	Thailand	Female	Mainland
USNM	308203	Thailand	Female	Mainland
USNM	355142	Thailand	Female	Mainland
USNM	355146	Thailand	Female	Mainland
USNM	355151	Thailand	Female	Mainland
USNM	355152	Thailand	Female	Mainland
USNM	355153	Thailand	Female	Mainland
USNM	355155	Thailand	Female	Mainland
USNM	355156	Thailand	Female	Mainland
USNM	355157	Thailand	Female	Mainland
USNM	355159	Thailand	Female	Mainland
USNM	355160	Thailand	Female	Mainland
USNM	355162	Thailand	Female	Mainland
USNM	355167	Thailand	Female	Mainland
USNM	355171	Thailand	Female	Mainland
USNM	355172	Thailand	Female	Mainland
USNM	355177	Thailand	Female	Mainland
USNM	355178	Thailand	Female	Mainland
USNM	355179	Thailand	Female	Mainland
USNM	355180	Thailand	Female	Mainland
USNM	533417	Thailand	Female	Mainland
USNM	533418	Thailand	Female	Mainland
USNM	533419	Thailand	Female	Mainland
USNM	533420	Thailand	Female	Mainland
USNM	533422	Thailand	Female	Mainland
USNM	533426	Thailand	Female	Mainland
USNM	533428	Thailand	Female	Mainland
USNM	533430	Thailand	Female	Mainland
USNM	533431	Thailand	Female	Mainland
USNM	533432	Thailand	Female	Mainland
USNM	294398	Thailand	Male	Mainland
USNM	355145	Thailand	Male	Mainland
USNM	355170	Thailand	Male	Mainland
USNM	201543	Thailand	Male	Mainland
USNM	201544	Thailand	Male	Mainland
USNM	221538	Thailand	Male	Mainland
USNM	221541	Thailand	Male	Mainland
USNM	241456	Thailand	Male	Mainland

Institute	Specimen	Island	Sex	Biogeographical region
USNM	241458	Thailand	Male	Mainland
USNM	251708	Thailand	Male	Mainland
USNM	252264	Thailand	Male	Mainland
USNM	267250	Thailand	Male	Mainland
USNM	294349	Thailand	Male	Mainland
USNM	294353	Thailand	Male	Mainland
USNM	294401	Thailand	Male	Mainland
USNM	294917	Thailand	Male	Mainland
USNM	294918	Thailand	Male	Mainland
USNM	294921	Thailand	Male	Mainland
USNM	294922	Thailand	Male	Mainland
USNM	294923	Thailand	Male	Mainland
USNM	294924	Thailand	Male	Mainland
USNM	294926	Thailand	Male	Mainland
USNM	294927	Thailand	Male	Mainland
USNM	296551	Thailand	Male	Mainland
USNM	296552	Thailand	Male	Mainland
USNM	296553	Thailand	Male	Mainland
USNM	297112	Thailand	Male	Mainland
USNM	297113	Thailand	Male	Mainland
USNM	297114	Thailand	Male	Mainland
USNM	297120	Thailand	Male	Mainland
USNM	297123	Thailand	Male	Mainland
USNM	297125	Thailand	Male	Mainland
USNM	297128	Thailand	Male	Mainland
USNM	297129	Thailand	Male	Mainland
USNM	297132	Thailand	Male	Mainland
USNM	297134	Thailand	Male	Mainland
USNM	308200	Thailand	Male	Mainland
USNM	308202	Thailand	Male	Mainland
USNM	355141	Thailand	Male	Mainland
USNM	355143	Thailand	Male	Mainland
USNM	355144	Thailand	Male	Mainland
USNM	355147	Thailand	Male	Mainland
USNM	355148	Thailand	Male	Mainland
USNM	355149	Thailand	Male	Mainland
USNM	355150	Thailand	Male	Mainland
USNM	355158	Thailand	Male	Mainland
USNM	355164	Thailand	Male	Mainland
USNM	355165	Thailand	Male	Mainland
USNM	355166	Thailand	Male	Mainland
USNM	355168	Thailand	Male	Mainland
USNM	355169	Thailand	Male	Mainland
USNM	355173	Thailand	Male	Mainland
USNM	355174	Thailand	Male	Mainland
USNM	533416	Thailand	Male	Mainland
USNM	533421	Thailand	Male	Mainland
USNM	533425	Thailand	Male	Mainland
USNM	533427	Thailand	Male	Mainland
USNM	533429	Thailand	Male	Mainland
USNM	533433	Thailand	Male	Mainland
USNM	533434	Thailand	Male	Mainland
USNM	355163	Thailand	Unknown	Mainland
USNM	294351	Thailand	Unknown	Mainland
USNM	296556	Thailand	Unknown	Mainland
USNM	308199	Thailand	Unknown	Mainland
USNM	356493	Vietnam	Female	Mainland
USNM	356496	Vietnam	Female	Mainland
USNM	357601	Vietnam	Female	Mainland
USNM	321152	Vietnam	Female	Mainland
USNM	321153	Vietnam	Female	Mainland
USNM	321154	Vietnam	Female	Mainland
USNM	321155	Vietnam	Female	Mainland
USNM	321156	Vietnam	Female	Mainland
USNM	321161	Vietnam	Female	Mainland
USNM	321165	Vietnam	Female	Mainland

Institute	Specimen	Island	Sex	Biogeographical region
USNM	321166	Vietnam	Female	Mainland
USNM	321167	Vietnam	Female	Mainland
USNM	321168	Vietnam	Female	Mainland
USNM	321170	Vietnam	Female	Mainland
USNM	321173	Vietnam	Female	Mainland
USNM	321174	Vietnam	Female	Mainland
USNM	321175	Vietnam	Female	Mainland
USNM	321176	Vietnam	Female	Mainland
USNM	321177	Vietnam	Female	Mainland
USNM	321178	Vietnam	Female	Mainland
USNM	321179	Vietnam	Female	Mainland
USNM	321181	Vietnam	Female	Mainland
USNM	321183	Vietnam	Female	Mainland
USNM	321188	Vietnam	Female	Mainland
USNM	321193	Vietnam	Female	Mainland
USNM	321196	Vietnam	Female	Mainland
USNM	321197	Vietnam	Female	Mainland
USNM	321198	Vietnam	Female	Mainland
USNM	321200	Vietnam	Female	Mainland
USNM	321202	Vietnam	Female	Mainland
USNM	321207	Vietnam	Female	Mainland
USNM	321208	Vietnam	Female	Mainland
USNM	321210	Vietnam	Female	Mainland
USNM	321211	Vietnam	Female	Mainland
USNM	332462	Vietnam	Female	Mainland
USNM	332463	Vietnam	Female	Mainland
USNM	332464	Vietnam	Female	Mainland
USNM	334786	Vietnam	Female	Mainland
USNM	334787	Vietnam	Female	Mainland
USNM	334789	Vietnam	Female	Mainland
USNM	356482	Vietnam	Female	Mainland
USNM	356485	Vietnam	Female	Mainland
USNM	356486	Vietnam	Female	Mainland
USNM	356490	Vietnam	Female	Mainland
USNM	356801	Vietnam	Female	Mainland
USNM	356803	Vietnam	Female	Mainland
USNM	356804	Vietnam	Female	Mainland
USNM	356805	Vietnam	Female	Mainland
USNM	356913	Vietnam	Female	Mainland
USNM	356914	Vietnam	Female	Mainland
USNM	356917	Vietnam	Female	Mainland
USNM	356918	Vietnam	Female	Mainland
USNM	356920	Vietnam	Female	Mainland
USNM	357552	Vietnam	Female	Mainland
USNM	357021	Vietnam	Female	Mainland
USNM	357022	Vietnam	Female	Mainland
USNM	357024	Vietnam	Female	Mainland
USNM	357025	Vietnam	Female	Mainland
USNM	357027	Vietnam	Female	Mainland
USNM	357030	Vietnam	Female	Mainland
USNM	357033	Vietnam	Female	Mainland
USNM	357034	Vietnam	Female	Mainland
USNM	357035	Vietnam	Female	Mainland
USNM	357036	Vietnam	Female	Mainland
USNM	357038	Vietnam	Female	Mainland
USNM	357039	Vietnam	Female	Mainland
USNM	357040	Vietnam	Female	Mainland
USNM	357041	Vietnam	Female	Mainland
USNM	357046	Vietnam	Female	Mainland
USNM	357048	Vietnam	Female	Mainland
USNM	357049	Vietnam	Female	Mainland
USNM	357050	Vietnam	Female	Mainland
USNM	357054	Vietnam	Female	Mainland
USNM	357056	Vietnam	Female	Mainland
USNM	357060	Vietnam	Female	Mainland
USNM	357089	Vietnam	Female	Mainland

Institute	Specimen	Island	Sex	Biogeographical region
USNM	357591	Vietnam	Female	Mainland
USNM	357780	Vietnam	Female	Mainland
USNM	357782	Vietnam	Female	Mainland
USNM	357783	Vietnam	Female	Mainland
USNM	357786	Vietnam	Female	Mainland
USNM	358877	Vietnam	Female	Mainland
USNM	357292	Vietnam	Female	Mainland
USNM	357047	Vietnam	Female	Mainland
USNM	356494	Vietnam	Male	Mainland
USNM	321157	Vietnam	Male	Mainland
USNM	321158	Vietnam	Male	Mainland
USNM	321159	Vietnam	Male	Mainland
USNM	321160	Vietnam	Male	Mainland
USNM	321162	Vietnam	Male	Mainland
USNM	321163	Vietnam	Male	Mainland
USNM	321164	Vietnam	Male	Mainland
USNM	321169	Vietnam	Male	Mainland
USNM	321171	Vietnam	Male	Mainland
USNM	321172	Vietnam	Male	Mainland
USNM	334783	Vietnam	Male	Mainland
USNM	321185	Vietnam	Male	Mainland
USNM	321186	Vietnam	Male	Mainland
USNM	321187	Vietnam	Male	Mainland
USNM	321189	Vietnam	Male	Mainland
USNM	321190	Vietnam	Male	Mainland
USNM	321192	Vietnam	Male	Mainland
USNM	321199	Vietnam	Male	Mainland
USNM	321201	Vietnam	Male	Mainland
USNM	321203	Vietnam	Male	Mainland
USNM	321209	Vietnam	Male	Mainland
USNM	334784	Vietnam	Male	Mainland
USNM	334785	Vietnam	Male	Mainland
USNM	334788	Vietnam	Male	Mainland
USNM	334790	Vietnam	Male	Mainland
USNM	334791	Vietnam	Male	Mainland
USNM	356488	Vietnam	Male	Mainland
USNM	356489	Vietnam	Male	Mainland
USNM	356492	Vietnam	Male	Mainland
USNM	356802	Vietnam	Male	Mainland
USNM	357551	Vietnam	Male	Mainland
USNM	357019	Vietnam	Male	Mainland
USNM	357031	Vietnam	Male	Mainland
USNM	357032	Vietnam	Male	Mainland
USNM	357037	Vietnam	Male	Mainland
USNM	357043	Vietnam	Male	Mainland
USNM	357045	Vietnam	Male	Mainland
USNM	357051	Vietnam	Male	Mainland
USNM	357052	Vietnam	Male	Mainland
USNM	357053	Vietnam	Male	Mainland
USNM	357055	Vietnam	Male	Mainland
USNM	357057	Vietnam	Male	Mainland
USNM	355473	Vietnam	Male	Mainland
USNM	357788	Vietnam	Male	Mainland
USNM	357550	Vietnam	Male	Mainland
USNM	321182	Vietnam	Male	Mainland
USNM	321191	Vietnam	Male	Mainland
USNM	321194	Vietnam	Male	Mainland
USNM	321195	Vietnam	Male	Mainland
USNM	321204	Vietnam	Male	Mainland
USNM	321205	Vietnam	Male	Mainland
USNM	321206	Vietnam	Male	Mainland
USNM	356481	Vietnam	Male	Mainland
USNM	356483	Vietnam	Male	Mainland
USNM	356487	Vietnam	Male	Mainland
USNM	356491	Vietnam	Male	Mainland
USNM	356495	Vietnam	Male	Mainland

Institute	Specimen	Island	Sex	Biogeographical region
USNM	356800	Vietnam	Male	Mainland
USNM	356919	Vietnam	Male	Mainland
USNM	357020	Vietnam	Male	Mainland
USNM	357023	Vietnam	Male	Mainland
USNM	357026	Vietnam	Male	Mainland
USNM	357028	Vietnam	Male	Mainland
USNM	357029	Vietnam	Male	Mainland
USNM	357042	Vietnam	Male	Mainland
USNM	357044	Vietnam	Male	Mainland
USNM	357058	Vietnam	Male	Mainland
USNM	357059	Vietnam	Male	Mainland
USNM	357224	Vietnam	Male	Mainland
USNM	357663	Vietnam	Male	Mainland
USNM	357712	Vietnam	Male	Mainland
USNM	357781	Vietnam	Male	Mainland
USNM	357787	Vietnam	Male	Mainland
USNM	321180	Vietnam	Male	Mainland
USNM	356912	Vietnam	Male	Mainland
USNM	321184	Vietnam	Unknown	Mainland
USNM	277079	Bougainville	Female	Melanesia
USNM	276883	Bougainville	Female	Melanesia
USNM	276884	Bougainville	Female	Melanesia
USNM	276885	Bougainville	Female	Melanesia
USNM	276886	Bougainville	Female	Melanesia
USNM	276887	Bougainville	Female	Melanesia
USNM	276891	Bougainville	Female	Melanesia
USNM	276892	Bougainville	Female	Melanesia
USNM	276893	Bougainville	Female	Melanesia
USNM	277074	Bougainville	Female	Melanesia
USNM	277077	Bougainville	Female	Melanesia
USNM	276897	Bougainville	Female	Melanesia
USNM	276898	Bougainville	Female	Melanesia
USNM	276902	Bougainville	Female	Melanesia
USNM	276904	Bougainville	Female	Melanesia
USNM	276906	Bougainville	Female	Melanesia
USNM	276907	Bougainville	Female	Melanesia
USNM	276837	Bougainville	Female	Melanesia
USNM	276840	Bougainville	Female	Melanesia
USNM	276841	Bougainville	Female	Melanesia
USNM	276842	Bougainville	Female	Melanesia
USNM	276844	Bougainville	Female	Melanesia
USNM	276845	Bougainville	Female	Melanesia
USNM	276847	Bougainville	Female	Melanesia
USNM	276849	Bougainville	Female	Melanesia
USNM	276852	Bougainville	Female	Melanesia
USNM	276855	Bougainville	Female	Melanesia
USNM	276859	Bougainville	Female	Melanesia
USNM	276861	Bougainville	Female	Melanesia
USNM	276863	Bougainville	Female	Melanesia
USNM	276865	Bougainville	Female	Melanesia
USNM	276866	Bougainville	Female	Melanesia
USNM	276867	Bougainville	Female	Melanesia
USNM	276870	Bougainville	Female	Melanesia
USNM	276874	Bougainville	Female	Melanesia
USNM	276875	Bougainville	Female	Melanesia
USNM	276877	Bougainville	Female	Melanesia
USNM	276880	Bougainville	Female	Melanesia
USNM	277080	Bougainville	Male	Melanesia
USNM	276860	Bougainville	Male	Melanesia
USNM	276881	Bougainville	Male	Melanesia
USNM	276882	Bougainville	Male	Melanesia
USNM	276888	Bougainville	Male	Melanesia
USNM	276889	Bougainville	Male	Melanesia
USNM	276890	Bougainville	Male	Melanesia
USNM	276894	Bougainville	Male	Melanesia
USNM	276895	Bougainville	Male	Melanesia

Institute	Specimen	Island	Sex	Biogeographical region
USNM	276896	Bougainville	Male	Melanesia
USNM	277071	Bougainville	Male	Melanesia
USNM	277078	Bougainville	Male	Melanesia
USNM	277083	Bougainville	Male	Melanesia
USNM	276899	Bougainville	Male	Melanesia
USNM	276900	Bougainville	Male	Melanesia
USNM	276901	Bougainville	Male	Melanesia
USNM	276903	Bougainville	Male	Melanesia
USNM	276905	Bougainville	Male	Melanesia
USNM	276824	Bougainville	Male	Melanesia
USNM	276833	Bougainville	Male	Melanesia
USNM	276834	Bougainville	Male	Melanesia
USNM	276835	Bougainville	Male	Melanesia
USNM	276836	Bougainville	Male	Melanesia
USNM	276838	Bougainville	Male	Melanesia
USNM	276839	Bougainville	Male	Melanesia
USNM	276843	Bougainville	Male	Melanesia
USNM	276846	Bougainville	Male	Melanesia
USNM	276850	Bougainville	Male	Melanesia
USNM	276851	Bougainville	Male	Melanesia
USNM	276853	Bougainville	Male	Melanesia
USNM	276854	Bougainville	Male	Melanesia
USNM	276856	Bougainville	Male	Melanesia
USNM	276857	Bougainville	Male	Melanesia
USNM	276858	Bougainville	Male	Melanesia
USNM	276862	Bougainville	Male	Melanesia
USNM	276864	Bougainville	Male	Melanesia
USNM	276868	Bougainville	Male	Melanesia
USNM	276869	Bougainville	Male	Melanesia
USNM	276871	Bougainville	Male	Melanesia
USNM	276872	Bougainville	Male	Melanesia
USNM	276873	Bougainville	Male	Melanesia
USNM	276876	Bougainville	Male	Melanesia
USNM	276878	Bougainville	Male	Melanesia
USNM	276879	Bougainville	Male	Melanesia
USNM	277081	Bougainville	Male	Melanesia
USNM	277082	Bougainville	Male	Melanesia
USNM	277084	Bougainville	Male	Melanesia
USNM	276911	Emirau	Female	Melanesia
USNM	277073	Emirau	Male	Melanesia
USNM	276908	Emirau	Male	Melanesia
USNM	276909	Emirau	Male	Melanesia
USNM	276910	Emirau	Male	Melanesia
USNM	260694	Fiji	Female	Melanesia
USNM	260695	Fiji	Female	Melanesia
USNM	260693	Fiji	Male	Melanesia
USNM	3731	Fiji	Unknown	Melanesia
USNM	276740	Guadalcanal	Female	Melanesia
USNM	278077	Guadalcanal	Female	Melanesia
USNM	278080	Guadalcanal	Female	Melanesia
USNM	278082	Guadalcanal	Female	Melanesia
USNM	278084	Guadalcanal	Female	Melanesia
USNM	278086	Guadalcanal	Female	Melanesia
USNM	278087	Guadalcanal	Female	Melanesia
USNM	278088	Guadalcanal	Female	Melanesia
USNM	278093	Guadalcanal	Female	Melanesia
USNM	278096	Guadalcanal	Female	Melanesia
USNM	278102	Guadalcanal	Female	Melanesia
USNM	278104	Guadalcanal	Female	Melanesia
USNM	278106	Guadalcanal	Female	Melanesia
USNM	278110	Guadalcanal	Female	Melanesia
USNM	278111	Guadalcanal	Female	Melanesia
USNM	278112	Guadalcanal	Female	Melanesia
USNM	278113	Guadalcanal	Female	Melanesia
USNM	276741	Guadalcanal	Male	Melanesia
USNM	278073	Guadalcanal	Male	Melanesia

Institute	Specimen	Island	Sex	Biogeographical region
USNM	278074	Guadalcanal	Male	Melanesia
USNM	278075	Guadalcanal	Male	Melanesia
USNM	278076	Guadalcanal	Male	Melanesia
USNM	278078	Guadalcanal	Male	Melanesia
USNM	278079	Guadalcanal	Male	Melanesia
USNM	278081	Guadalcanal	Male	Melanesia
USNM	278083	Guadalcanal	Male	Melanesia
USNM	278085	Guadalcanal	Male	Melanesia
USNM	278089	Guadalcanal	Male	Melanesia
USNM	278090	Guadalcanal	Male	Melanesia
USNM	278091	Guadalcanal	Male	Melanesia
USNM	278092	Guadalcanal	Male	Melanesia
USNM	278094	Guadalcanal	Male	Melanesia
USNM	278095	Guadalcanal	Male	Melanesia
USNM	278097	Guadalcanal	Male	Melanesia
USNM	278098	Guadalcanal	Male	Melanesia
USNM	278099	Guadalcanal	Male	Melanesia
USNM	278100	Guadalcanal	Male	Melanesia
USNM	278101	Guadalcanal	Male	Melanesia
USNM	278103	Guadalcanal	Male	Melanesia
USNM	278105	Guadalcanal	Male	Melanesia
USNM	278107	Guadalcanal	Male	Melanesia
USNM	278108	Guadalcanal	Male	Melanesia
USNM	278109	Guadalcanal	Male	Melanesia
USNM	278114	Guadalcanal	Male	Melanesia
USNM	289914	Guadalcanal	Unknown	Melanesia
USNM	289915	Guadalcanal	Unknown	Melanesia
USNM	276750	New Caledonia	Female	Melanesia
USNM	276756	New Caledonia	Female	Melanesia
USNM	271510	New Caledonia	Female	Melanesia
USNM	271512	New Caledonia	Female	Melanesia
USNM	276744	New Caledonia	Female	Melanesia
USNM	276746	New Caledonia	Female	Melanesia
USNM	276748	New Caledonia	Female	Melanesia
USNM	276914	New Caledonia	Female	Melanesia
USNM	276915	New Caledonia	Female	Melanesia
USNM	276916	New Caledonia	Female	Melanesia
USNM	277069	New Caledonia	Female	Melanesia
USNM	271511	New Caledonia	Female	Melanesia
USNM	276762	New Caledonia	Female	Melanesia
USNM	276763	New Caledonia	Female	Melanesia
USNM	276764	New Caledonia	Female	Melanesia
USNM	276765	New Caledonia	Female	Melanesia
USNM	276766	New Caledonia	Female	Melanesia
USNM	276769	New Caledonia	Female	Melanesia
USNM	276771	New Caledonia	Female	Melanesia
USNM	276772	New Caledonia	Female	Melanesia
USNM	276773	New Caledonia	Female	Melanesia
USNM	276774	New Caledonia	Female	Melanesia
USNM	276785	New Caledonia	Female	Melanesia
USNM	276789	New Caledonia	Female	Melanesia
USNM	276751	New Caledonia	Female	Melanesia
USNM	276752	New Caledonia	Female	Melanesia
USNM	276753	New Caledonia	Female	Melanesia
USNM	276755	New Caledonia	Female	Melanesia
USNM	283354	New Caledonia	Female	Melanesia
USNM	283355	New Caledonia	Female	Melanesia
USNM	271500	New Caledonia	Female	Melanesia
USNM	271506	New Caledonia	Female	Melanesia
USNM	276760	New Caledonia	Female	Melanesia
USNM	271509	New Caledonia	Male	Melanesia
USNM	276745	New Caledonia	Male	Melanesia
USNM	276749	New Caledonia	Male	Melanesia
USNM	276912	New Caledonia	Male	Melanesia
USNM	276913	New Caledonia	Male	Melanesia
USNM	276757	New Caledonia	Male	Melanesia

Institute	Specimen	Island	Sex	Biogeographical region
USNM	276759	New Caledonia	Male	Melanesia
USNM	276761	New Caledonia	Male	Melanesia
USNM	276767	New Caledonia	Male	Melanesia
USNM	276770	New Caledonia	Male	Melanesia
USNM	276778	New Caledonia	Male	Melanesia
USNM	276779	New Caledonia	Male	Melanesia
USNM	276786	New Caledonia	Male	Melanesia
USNM	276788	New Caledonia	Male	Melanesia
USNM	276790	New Caledonia	Male	Melanesia
USNM	276791	New Caledonia	Male	Melanesia
USNM	276792	New Caledonia	Male	Melanesia
USNM	276754	New Caledonia	Male	Melanesia
USNM	271507	New Caledonia	Male	Melanesia
USNM	271508	New Caledonia	Male	Melanesia
USNM	357421	New Guinea	Female	Melanesia
USNM	357423	New Guinea	Female	Melanesia
USNM	277326	New Guinea	Female	Melanesia
USNM	277335	New Guinea	Female	Melanesia
USNM	277337	New Guinea	Female	Melanesia
USNM	277325	New Guinea	Female	Melanesia
USNM	357420	New Guinea	Female	Melanesia
USNM	277330	New Guinea	Female	Melanesia
USNM	277439	New Guinea	Female	Melanesia
USNM	277461	New Guinea	Female	Melanesia
USNM	277462	New Guinea	Female	Melanesia
USNM	277463	New Guinea	Female	Melanesia
USNM	295098	New Guinea	Female	Melanesia
USNM	295104	New Guinea	Female	Melanesia
USNM	295111	New Guinea	Female	Melanesia
USNM	283864	New Guinea	Female	Melanesia
USNM	283867	New Guinea	Female	Melanesia
USNM	277258	New Guinea	Female	Melanesia
USNM	357422	New Guinea	Male	Melanesia
USNM	357424	New Guinea	Male	Melanesia
USNM	277327	New Guinea	Male	Melanesia
USNM	277328	New Guinea	Male	Melanesia
USNM	277329	New Guinea	Male	Melanesia
USNM	277332	New Guinea	Male	Melanesia
USNM	277334	New Guinea	Male	Melanesia
USNM	277336	New Guinea	Male	Melanesia
USNM	277338	New Guinea	Male	Melanesia
USNM	295091	New Guinea	Male	Melanesia
USNM	295092	New Guinea	Male	Melanesia
USNM	277324	New Guinea	Male	Melanesia
USNM	277331	New Guinea	Male	Melanesia
USNM	277333	New Guinea	Male	Melanesia
USNM	277440	New Guinea	Male	Melanesia
USNM	277464	New Guinea	Male	Melanesia
USNM	277465	New Guinea	Male	Melanesia
USNM	283861	New Guinea	Male	Melanesia
USNM	283862	New Guinea	Male	Melanesia
USNM	295094	New Guinea	Male	Melanesia
USNM	295095	New Guinea	Male	Melanesia
USNM	295096	New Guinea	Male	Melanesia
USNM	295100	New Guinea	Male	Melanesia
USNM	295103	New Guinea	Male	Melanesia
USNM	283863	New Guinea	Male	Melanesia
USNM	283865	New Guinea	Male	Melanesia
USNM	283866	New Guinea	Male	Melanesia
USNM	283868	New Guinea	Male	Melanesia
USNM	276646	New Guinea	Male	Melanesia
USNM	276647	New Guinea	Male	Melanesia
USNM	580043	New Ireland	Female	Melanesia
USNM	580044	New Ireland	Female	Melanesia
USNM	580039	New Ireland	Male	Melanesia
USNM	580042	New Ireland	Male	Melanesia

Institute	Specimen	Island	Sex	Biogeographical region
USNM	278710	Ontong Java	Female	Melanesia
USNM	278711	Ontong Java	Female	Melanesia
USNM	278707	Ontong Java	Female	Melanesia
USNM	278709	Ontong Java	Male	Melanesia
USNM	278708	Ontong Java	Male	Melanesia
USNM	277308	Padaido Islands	Male	Melanesia
USNM	278713	Ugi	Male	Melanesia
USNM	271518	Vanuatu	Female	Melanesia
USNM	276919	Vanuatu	Female	Melanesia
USNM	276921	Vanuatu	Female	Melanesia
USNM	277930	Vanuatu	Female	Melanesia
USNM	277931	Vanuatu	Female	Melanesia
USNM	277932	Vanuatu	Female	Melanesia
USNM	277943	Vanuatu	Female	Melanesia
USNM	277950	Vanuatu	Female	Melanesia
USNM	277951	Vanuatu	Female	Melanesia
USNM	277953	Vanuatu	Female	Melanesia
USNM	277954	Vanuatu	Female	Melanesia
USNM	277955	Vanuatu	Female	Melanesia
USNM	276922	Vanuatu	Female	Melanesia
USNM	277924	Vanuatu	Female	Melanesia
USNM	277926	Vanuatu	Female	Melanesia
USNM	277927	Vanuatu	Female	Melanesia
USNM	277928	Vanuatu	Female	Melanesia
USNM	271513	Vanuatu	Female	Melanesia
USNM	271514	Vanuatu	Female	Melanesia
USNM	271517	Vanuatu	Female	Melanesia
USNM	276925	Vanuatu	Female	Melanesia
USNM	276917	Vanuatu	Male	Melanesia
USNM	276918	Vanuatu	Male	Melanesia
USNM	276920	Vanuatu	Male	Melanesia
USNM	276832	Vanuatu	Male	Melanesia
USNM	277929	Vanuatu	Male	Melanesia
USNM	277933	Vanuatu	Male	Melanesia
USNM	277934	Vanuatu	Male	Melanesia
USNM	277941	Vanuatu	Male	Melanesia
USNM	277942	Vanuatu	Male	Melanesia
USNM	277944	Vanuatu	Male	Melanesia
USNM	276923	Vanuatu	Male	Melanesia
USNM	276924	Vanuatu	Male	Melanesia
USNM	277020	Vanuatu	Male	Melanesia
USNM	277070	Vanuatu	Male	Melanesia
USNM	277925	Vanuatu	Male	Melanesia
USNM	271515	Vanuatu	Male	Melanesia
USNM	271516	Vanuatu	Male	Melanesia
USNM	277949	Vanuatu	Male	Melanesia
USNM	277952	Vanuatu	Male	Melanesia
USNM	277019	Vanuatu	Male	Melanesia
USNM	302971	Ant Atoll	Female	Micronesia
USNM	291948	Arno Atoll	Female	Micronesia
USNM	291949	Arno Atoll	Female	Micronesia
USNM	291950	Arno Atoll	Female	Micronesia
USNM	291951	Arno Atoll	Female	Micronesia
USNM	291952	Arno Atoll	Male	Micronesia
USNM	356301	Babelthuap Island	Female	Micronesia
USNM	356303	Babelthuap Island	Female	Micronesia
USNM	356304	Babelthuap Island	Female	Micronesia
USNM	356302	Babelthuap Island	Male	Micronesia
USNM	279480	Belau	Female	Micronesia
USNM	279481	Belau	Female	Micronesia
USNM	279482	Belau	Female	Micronesia
USNM	279483	Belau	Female	Micronesia
USNM	279484	Belau	Female	Micronesia
USNM	308258	Belau	Male	Micronesia
USNM	361455	Bikar Atoll	Female	Micronesia
USNM	361457	Bikar Atoll	Female	Micronesia

Institute	Specimen	Island	Sex	Biogeographical region
USNM	361466	Bikar Atoll	Female	Micronesia
USNM	362189	Bikar Atoll	Female	Micronesia
USNM	362190	Bikar Atoll	Female	Micronesia
USNM	362191	Bikar Atoll	Female	Micronesia
USNM	361461	Bikar Atoll	Male	Micronesia
USNM	361464	Bikar Atoll	Male	Micronesia
USNM	361460	Bikar Atoll	Male	Micronesia
USNM	361456	Bikar Atoll	Male	Micronesia
USNM	361463	Bikar Atoll	Male	Micronesia
USNM	361465	Bikar Atoll	Male	Micronesia
USNM	361462	Bikar Atoll	Unknown	Micronesia
USNM	302970	Caroline Islands	Female	Micronesia
USNM	282219	Caroline Islands	Female	Micronesia
USNM	282220	Caroline Islands	Female	Micronesia
USNM	282217	Caroline Islands	Female	Micronesia
USNM	278810	Caroline Islands	Female	Micronesia
USNM	278814	Caroline Islands	Female	Micronesia
USNM	278815	Caroline Islands	Female	Micronesia
USNM	278819	Caroline Islands	Female	Micronesia
USNM	278821	Caroline Islands	Female	Micronesia
USNM	278828	Caroline Islands	Female	Micronesia
USNM	278830	Caroline Islands	Female	Micronesia
USNM	278832	Caroline Islands	Female	Micronesia
USNM	278836	Caroline Islands	Female	Micronesia
USNM	278838	Caroline Islands	Female	Micronesia
USNM	278841	Caroline Islands	Female	Micronesia
USNM	278846	Caroline Islands	Female	Micronesia
USNM	278847	Caroline Islands	Female	Micronesia
USNM	278848	Caroline Islands	Female	Micronesia
USNM	361451	Caroline Islands	Female	Micronesia
USNM	278794	Caroline Islands	Female	Micronesia
USNM	278802	Caroline Islands	Female	Micronesia
USNM	278807	Caroline Islands	Female	Micronesia
USNM	278850	Caroline Islands	Female	Micronesia
USNM	282216	Caroline Islands	Male	Micronesia
USNM	278808	Caroline Islands	Male	Micronesia
USNM	278809	Caroline Islands	Male	Micronesia
USNM	278811	Caroline Islands	Male	Micronesia
USNM	278812	Caroline Islands	Male	Micronesia
USNM	278813	Caroline Islands	Male	Micronesia
USNM	278816	Caroline Islands	Male	Micronesia
USNM	278817	Caroline Islands	Male	Micronesia
USNM	278818	Caroline Islands	Male	Micronesia
USNM	278820	Caroline Islands	Male	Micronesia
USNM	278822	Caroline Islands	Male	Micronesia
USNM	278823	Caroline Islands	Male	Micronesia
USNM	278824	Caroline Islands	Male	Micronesia
USNM	278825	Caroline Islands	Male	Micronesia
USNM	278826	Caroline Islands	Male	Micronesia
USNM	278827	Caroline Islands	Male	Micronesia
USNM	278829	Caroline Islands	Male	Micronesia
USNM	278831	Caroline Islands	Male	Micronesia
USNM	278834	Caroline Islands	Male	Micronesia
USNM	278835	Caroline Islands	Male	Micronesia
USNM	278837	Caroline Islands	Male	Micronesia
USNM	278839	Caroline Islands	Male	Micronesia
USNM	278840	Caroline Islands	Male	Micronesia
USNM	278842	Caroline Islands	Male	Micronesia
USNM	278843	Caroline Islands	Male	Micronesia
USNM	278844	Caroline Islands	Male	Micronesia
USNM	278845	Caroline Islands	Male	Micronesia
USNM	278849	Caroline Islands	Male	Micronesia
USNM	361450	Caroline Islands	Male	Micronesia
USNM	278791	Caroline Islands	Male	Micronesia
USNM	278792	Caroline Islands	Male	Micronesia
USNM	278793	Caroline Islands	Male	Micronesia

Institute	Specimen	Island	Sex	Biogeographical region
USNM	278795	Caroline Islands	Male	Micronesia
USNM	278796	Caroline Islands	Male	Micronesia
USNM	278797	Caroline Islands	Male	Micronesia
USNM	278798	Caroline Islands	Male	Micronesia
USNM	278799	Caroline Islands	Male	Micronesia
USNM	278803	Caroline Islands	Male	Micronesia
USNM	278804	Caroline Islands	Male	Micronesia
USNM	278805	Caroline Islands	Male	Micronesia
USNM	278806	Caroline Islands	Male	Micronesia
USNM	303316	Caroline Islands	Male	Micronesia
USNM	278851	Caroline Islands	Male	Micronesia
USNM	278852	Caroline Islands	Male	Micronesia
USNM	278853	Caroline Islands	Male	Micronesia
USNM	278854	Caroline Islands	Male	Micronesia
USNM	278918	Guam	Female	Micronesia
USNM	278439	Guam	Female	Micronesia
USNM	278920	Guam	Female	Micronesia
USNM	278922	Guam	Female	Micronesia
USNM	278926	Guam	Female	Micronesia
USNM	278927	Guam	Female	Micronesia
USNM	278928	Guam	Female	Micronesia
USNM	278932	Guam	Female	Micronesia
USNM	278933	Guam	Female	Micronesia
USNM	278936	Guam	Female	Micronesia
USNM	278938	Guam	Female	Micronesia
USNM	277470	Guam	Female	Micronesia
USNM	278365	Guam	Female	Micronesia
USNM	278369	Guam	Female	Micronesia
USNM	278374	Guam	Female	Micronesia
USNM	278375	Guam	Female	Micronesia
USNM	278437	Guam	Female	Micronesia
USNM	278906	Guam	Female	Micronesia
USNM	278909	Guam	Female	Micronesia
USNM	278910	Guam	Female	Micronesia
USNM	278438	Guam	Male	Micronesia
USNM	278917	Guam	Male	Micronesia
USNM	278919	Guam	Male	Micronesia
USNM	278921	Guam	Male	Micronesia
USNM	278923	Guam	Male	Micronesia
USNM	278924	Guam	Male	Micronesia
USNM	278925	Guam	Male	Micronesia
USNM	278929	Guam	Male	Micronesia
USNM	278930	Guam	Male	Micronesia
USNM	278931	Guam	Male	Micronesia
USNM	278934	Guam	Male	Micronesia
USNM	278935	Guam	Male	Micronesia
USNM	278937	Guam	Male	Micronesia
USNM	277466	Guam	Male	Micronesia
USNM	277468	Guam	Male	Micronesia
USNM	277471	Guam	Male	Micronesia
USNM	277472	Guam	Male	Micronesia
USNM	277473	Guam	Male	Micronesia
USNM	278366	Guam	Male	Micronesia
USNM	278367	Guam	Male	Micronesia
USNM	278368	Guam	Male	Micronesia
USNM	278370	Guam	Male	Micronesia
USNM	278371	Guam	Male	Micronesia
USNM	278372	Guam	Male	Micronesia
USNM	278373	Guam	Male	Micronesia
USNM	278376	Guam	Male	Micronesia
USNM	278377	Guam	Male	Micronesia
USNM	278378	Guam	Male	Micronesia
USNM	278430	Guam	Male	Micronesia
USNM	278433	Guam	Male	Micronesia
USNM	278434	Guam	Male	Micronesia
USNM	278907	Guam	Male	Micronesia

Institute	Specimen	Island	Sex	Biogeographical region
USNM	278908	Guam	Male	Micronesia
USNM	278911	Guam	Male	Micronesia
USNM	278912	Guam	Male	Micronesia
USNM	278913	Guam	Male	Micronesia
USNM	278914	Guam	Male	Micronesia
USNM	278915	Guam	Male	Micronesia
USNM	278916	Guam	Male	Micronesia
USNM	278428	Guam	Male	Micronesia
USNM	278429	Guam	Male	Micronesia
USNM	530805	Guguan Island	Male	Micronesia
USNM	530806	Guguan Island	Male	Micronesia
USNM	279397	Koror Island	Female	Micronesia
USNM	279472	Koror Island	Female	Micronesia
USNM	279476	Koror Island	Female	Micronesia
USNM	279477	Koror Island	Female	Micronesia
USNM	279478	Koror Island	Female	Micronesia
USNM	279479	Koror Island	Female	Micronesia
USNM	279398	Koror Island	Male	Micronesia
USNM	279393	Koror Island	Male	Micronesia
USNM	279394	Koror Island	Male	Micronesia
USNM	279395	Koror Island	Male	Micronesia
USNM	279396	Koror Island	Male	Micronesia
USNM	279399	Koror Island	Male	Micronesia
USNM	279471	Koror Island	Male	Micronesia
USNM	279473	Koror Island	Male	Micronesia
USNM	279474	Koror Island	Male	Micronesia
USNM	279475	Koror Island	Male	Micronesia
USNM	302969	Kosrae	Female	Micronesia
USNM	297272	Kosrae	Male	Micronesia
USNM	297273	Kosrae	Male	Micronesia
USNM	297274	Kosrae	Male	Micronesia
USNM	306278	Majuro Atoll	Female	Micronesia
USNM	306274	Majuro Atoll	Male	Micronesia
USNM	306275	Majuro Atoll	Male	Micronesia
USNM	306276	Majuro Atoll	Male	Micronesia
USNM	306277	Majuro Atoll	Male	Micronesia
USNM	308266	Majuro Atoll	Male	Micronesia
USNM	308268	Majuro Atoll	Male	Micronesia
USNM	308269	Majuro Atoll	Male	Micronesia
USNM	308270	Majuro Atoll	Male	Micronesia
USNM	308267	Majuro Atoll	Male	Micronesia
USNM	276634	Marshall Islands	Female	Micronesia
USNM	276635	Marshall Islands	Female	Micronesia
USNM	538393	Marshall Islands	Female	Micronesia
USNM	538394	Marshall Islands	Female	Micronesia
USNM	276632	Marshall Islands	Male	Micronesia
USNM	276633	Marshall Islands	Male	Micronesia
USNM	276636	Marshall Islands	Unknown	Micronesia
USNM	278675	Peleliu Island	Male	Micronesia
USNM	306280	Pohnpei Island	Female	Micronesia
USNM	306283	Pohnpei Island	Female	Micronesia
USNM	306281	Pohnpei Island	Female	Micronesia
USNM	302009	Pohnpei Island	Female	Micronesia
USNM	302011	Pohnpei Island	Female	Micronesia
USNM	302960	Pohnpei Island	Female	Micronesia
USNM	302961	Pohnpei Island	Female	Micronesia
USNM	302963	Pohnpei Island	Female	Micronesia
USNM	302964	Pohnpei Island	Female	Micronesia
USNM	302966	Pohnpei Island	Female	Micronesia
USNM	302967	Pohnpei Island	Female	Micronesia
USNM	302968	Pohnpei Island	Female	Micronesia
USNM	283114	Pohnpei Island	Female	Micronesia
USNM	301995	Pohnpei Island	Female	Micronesia
USNM	301996	Pohnpei Island	Female	Micronesia
USNM	301998	Pohnpei Island	Female	Micronesia
USNM	301999	Pohnpei Island	Female	Micronesia

Institute	Specimen	Island	Sex	Biogeographical region
USNM	302002	Pohnpei Island	Female	Micronesia
USNM	302003	Pohnpei Island	Female	Micronesia
USNM	302005	Pohnpei Island	Female	Micronesia
USNM	302006	Pohnpei Island	Female	Micronesia
USNM	302007	Pohnpei Island	Female	Micronesia
USNM	302008	Pohnpei Island	Female	Micronesia
USNM	306279	Pohnpei Island	Male	Micronesia
USNM	302010	Pohnpei Island	Male	Micronesia
USNM	302962	Pohnpei Island	Male	Micronesia
USNM	302965	Pohnpei Island	Male	Micronesia
USNM	306282	Pohnpei Island	Male	Micronesia
USNM	301997	Pohnpei Island	Male	Micronesia
USNM	302000	Pohnpei Island	Male	Micronesia
USNM	302001	Pohnpei Island	Male	Micronesia
USNM	302004	Pohnpei Island	Male	Micronesia
USNM	277072	Ponam	Female	Micronesia
USNM	282246	Rongelap Atoll	Female	Micronesia
USNM	282247	Rongelap Atoll	Female	Micronesia
USNM	282839	Rongerik Atoll	Male	Micronesia
USNM	282840	Rongerik Atoll	Male	Micronesia
USNM	282844	Rongerik Atoll	Male	Micronesia
USNM	282845	Rongerik Atoll	Male	Micronesia
USNM	279124	Saipan Island	Female	Micronesia
USNM	298365	Saipan Island	Female	Micronesia
USNM	298366	Saipan Island	Female	Micronesia
USNM	277559	Saipan Island	Female	Micronesia
USNM	277560	Saipan Island	Female	Micronesia
USNM	277706	Saipan Island	Female	Micronesia
USNM	277708	Saipan Island	Female	Micronesia
USNM	277707	Saipan Island	Male	Micronesia
USNM	361458	Taka Atoll	Male	Micronesia
USNM	361459	Taka Atoll	Male	Micronesia
USNM	277709	Tinian Island	Male	Micronesia
USNM	277710	Tinian Island	Male	Micronesia
USNM	243874	Wake Island	Female	Micronesia
USNM	243887	Wake Island	Female	Micronesia
USNM	243889	Wake Island	Female	Micronesia
USNM	243876	Wake Island	Female	Micronesia
USNM	243877	Wake Island	Female	Micronesia
USNM	243878	Wake Island	Female	Micronesia
USNM	243883	Wake Island	Female	Micronesia
USNM	265392	Wake Island	Female	Micronesia
USNM	243875	Wake Island	Male	Micronesia
USNM	243879	Wake Island	Male	Micronesia
USNM	243880	Wake Island	Male	Micronesia
USNM	243881	Wake Island	Male	Micronesia
USNM	243882	Wake Island	Male	Micronesia
USNM	265391	Wake Island	Male	Micronesia
USNM	243864	Wake Island	Male	Micronesia
USNM	243868	Wake Island	Male	Micronesia
USNM	243869	Wake Island	Male	Micronesia
USNM	243870	Wake Island	Male	Micronesia
USNM	243872	Wake Island	Male	Micronesia
USNM	243873	Wake Island	Male	Micronesia
USNM	243885	Wake Island	Male	Micronesia
USNM	243886	Wake Island	Male	Micronesia
USNM	300636	Kapingamarangi Atc	Female	Outlier (Polynesian Outlier)
USNM	300638	Kapingamarangi Atc	Female	Outlier (Polynesian Outlier)
USNM	300637	Kapingamarangi Atc	Male	Outlier (Polynesian Outlier)
USNM	532911	American Samoa	Female	Polynesia
USNM	532912	American Samoa	Female	Polynesia
USNM	532904	American Samoa	Female	Polynesia
USNM	532901	American Samoa	Female	Polynesia
USNM	532902	American Samoa	Female	Polynesia
USNM	532905	American Samoa	Female	Polynesia
USNM	532906	American Samoa	Female	Polynesia

Institute	Specimen	Island	Sex	Biogeographical region
USNM	532907	American Samoa	Female	Polynesia
USNM	532903	American Samoa	Male	Polynesia
USNM	532908	American Samoa	Male	Polynesia
USNM	532909	American Samoa	Male	Polynesia
USNM	532910	American Samoa	Male	Polynesia
USNM	532913	American Samoa	Male	Polynesia
USNM	360997	Atafu Atoll	Female	Polynesia
USNM	360998	Atafu Atoll	Female	Polynesia
USNM	532890	Aunu'U Island	Female	Polynesia
USNM	532894	Aunu'U Island	Female	Polynesia
USNM	532888	Aunu'U Island	Male	Polynesia
USNM	532889	Aunu'U Island	Male	Polynesia
USNM	532891	Aunu'U Island	Male	Polynesia
USNM	532892	Aunu'U Island	Male	Polynesia
USNM	532893	Aunu'U Island	Male	Polynesia
USNM	282127	Bikini Atoll	Female	Polynesia
USNM	282340	Bikini Atoll	Female	Polynesia
USNM	536411	Bikini Atoll	Female	Polynesia
USNM	282125	Bikini Atoll	Male	Polynesia
USNM	282126	Bikini Atoll	Male	Polynesia
USNM	282128	Bikini Atoll	Male	Polynesia
USNM	282129	Bikini Atoll	Male	Polynesia
USNM	282130	Bikini Atoll	Male	Polynesia
USNM	282131	Bikini Atoll	Male	Polynesia
USNM	282132	Bikini Atoll	Male	Polynesia
USNM	282133	Bikini Atoll	Male	Polynesia
USNM	536410	Bikini Atoll	Male	Polynesia
USNM	536412	Bikini Atoll	Male	Polynesia
USNM	536413	Bikini Atoll	Male	Polynesia
USNM	338660	Birnie Atoll	Female	Polynesia
USNM	338661	Birnie Atoll	Female	Polynesia
USNM	338662	Birnie Atoll	Female	Polynesia
USNM	338664	Birnie Atoll	Female	Polynesia
USNM	338663	Birnie Atoll	Female	Polynesia
USNM	338673	Birnie Atoll	Female	Polynesia
USNM	338658	Birnie Atoll	Male	Polynesia
USNM	361454	Birnie Atoll	Male	Polynesia
USNM	338665	Birnie Atoll	Male	Polynesia
USNM	338667	Birnie Atoll	Male	Polynesia
USNM	338670	Birnie Atoll	Male	Polynesia
USNM	338672	Birnie Atoll	Male	Polynesia
USNM	338674	Birnie Atoll	Male	Polynesia
USNM	338675	Birnie Atoll	Male	Polynesia
USNM	338679	Birnie Atoll	Male	Polynesia
USNM	338680	Birnie Atoll	Male	Polynesia
USNM	338682	Birnie Atoll	Male	Polynesia
USNM	361452	Birnie Atoll	Male	Polynesia
USNM	338666	Birnie Atoll	Male	Polynesia
USNM	338668	Birnie Atoll	Male	Polynesia
USNM	338669	Birnie Atoll	Male	Polynesia
USNM	338676	Birnie Atoll	Male	Polynesia
USNM	338677	Birnie Atoll	Male	Polynesia
USNM	338678	Birnie Atoll	Male	Polynesia
USNM	338671	Birnie Atoll	Unknown	Polynesia
USNM	338681	Birnie Atoll	Unknown	Polynesia
USNM	338688	Christmas Island	Male	Polynesia
USNM	338685	Christmas Island	Unknown	Polynesia
USNM	338687	Christmas Island	Unknown	Polynesia
USNM	538392	Enewetok Atoll	Female	Polynesia
USNM	448344	Enewetok Atoll	Male	Polynesia
USNM	360994	Fakaofo Atoll	Female	Polynesia
USNM	360993	Fakaofo Atoll	Male	Polynesia
USNM	257425	Hatutaa Island	Female	Polynesia
USNM	257427	Hatutaa Island	Female	Polynesia
USNM	257428	Hatutaa Island	Male	Polynesia
USNM	257426	Hatutaa Island	Male	Polynesia

Institute	Specimen	Island	Sex	Biogeographical region
USNM	324887	Hawaii	Female	Polynesia
USNM	324890	Hawaii	Female	Polynesia
USNM	324891	Hawaii	Female	Polynesia
USNM	324892	Hawaii	Female	Polynesia
USNM	324893	Hawaii	Female	Polynesia
USNM	324896	Hawaii	Female	Polynesia
USNM	324897	Hawaii	Female	Polynesia
USNM	271390	Hawaii	Female	Polynesia
USNM	271391	Hawaii	Female	Polynesia
USNM	257936	Hawaii	Female	Polynesia
USNM	257939	Hawaii	Female	Polynesia
USNM	257940	Hawaii	Female	Polynesia
USNM	257942	Hawaii	Female	Polynesia
USNM	257943	Hawaii	Female	Polynesia
USNM	257944	Hawaii	Female	Polynesia
USNM	257945	Hawaii	Female	Polynesia
USNM	324885	Hawaii	Male	Polynesia
USNM	324886	Hawaii	Male	Polynesia
USNM	324888	Hawaii	Male	Polynesia
USNM	324889	Hawaii	Male	Polynesia
USNM	324894	Hawaii	Male	Polynesia
USNM	324895	Hawaii	Male	Polynesia
USNM	556523	Hawaii	Male	Polynesia
USNM	271388	Hawaii	Male	Polynesia
USNM	271389	Hawaii	Male	Polynesia
USNM	257937	Hawaii	Male	Polynesia
USNM	257938	Hawaii	Male	Polynesia
USNM	257941	Hawaii	Male	Polynesia
USNM	257946	Hawaii	Male	Polynesia
USNM	257947	Hawaii	Male	Polynesia
USNM	99673	Hawaiian Islands	Unknown	Polynesia
USNM	361431	Kanton Island	Female	Polynesia
USNM	361433	Kanton Island	Female	Polynesia
USNM	361436	Kanton Island	Female	Polynesia
USNM	361437	Kanton Island	Female	Polynesia
USNM	361439	Kanton Island	Female	Polynesia
USNM	361432	Kanton Island	Male	Polynesia
USNM	361434	Kanton Island	Male	Polynesia
USNM	269149	Kanton Island	Unknown	Polynesia
USNM	361435	Kanton Island	Unknown	Polynesia
USNM	570868	Kauai	Female	Polynesia
USNM	338683	Kiritimati	Unknown	Polynesia
USNM	338684	Kiritimati	Unknown	Polynesia
USNM	338686	Kiritimati	Unknown	Polynesia
USNM	338629	Kure Atoll	Female	Polynesia
USNM	361417	Kure Atoll	Female	Polynesia
USNM	361425	Kure Atoll	Female	Polynesia
USNM	355886	Kure Atoll	Female	Polynesia
USNM	243900	Kure Atoll	Female	Polynesia
USNM	243901	Kure Atoll	Female	Polynesia
USNM	338631	Kure Atoll	Female	Polynesia
USNM	355879	Kure Atoll	Female	Polynesia
USNM	361376	Kure Atoll	Female	Polynesia
USNM	361378	Kure Atoll	Female	Polynesia
USNM	361379	Kure Atoll	Female	Polynesia
USNM	361381	Kure Atoll	Female	Polynesia
USNM	361382	Kure Atoll	Female	Polynesia
USNM	361383	Kure Atoll	Female	Polynesia
USNM	355757	Kure Atoll	Female	Polynesia
USNM	355760	Kure Atoll	Female	Polynesia
USNM	355761	Kure Atoll	Female	Polynesia
USNM	355766	Kure Atoll	Female	Polynesia
USNM	355771	Kure Atoll	Female	Polynesia
USNM	355772	Kure Atoll	Female	Polynesia
USNM	355878	Kure Atoll	Female	Polynesia
USNM	355880	Kure Atoll	Female	Polynesia

Institute	Specimen	Island	Sex	Biogeographical region
USNM	355881	Kure Atoll	Female	Polynesia
USNM	355883	Kure Atoll	Female	Polynesia
USNM	355884	Kure Atoll	Female	Polynesia
USNM	243890	Kure Atoll	Male	Polynesia
USNM	243891	Kure Atoll	Male	Polynesia
USNM	243892	Kure Atoll	Male	Polynesia
USNM	243893	Kure Atoll	Male	Polynesia
USNM	243894	Kure Atoll	Male	Polynesia
USNM	243895	Kure Atoll	Male	Polynesia
USNM	243896	Kure Atoll	Male	Polynesia
USNM	243897	Kure Atoll	Male	Polynesia
USNM	243898	Kure Atoll	Male	Polynesia
USNM	243899	Kure Atoll	Male	Polynesia
USNM	243902	Kure Atoll	Male	Polynesia
USNM	338630	Kure Atoll	Male	Polynesia
USNM	338632	Kure Atoll	Male	Polynesia
USNM	338653	Kure Atoll	Male	Polynesia
USNM	338654	Kure Atoll	Male	Polynesia
USNM	355876	Kure Atoll	Male	Polynesia
USNM	361377	Kure Atoll	Male	Polynesia
USNM	361380	Kure Atoll	Male	Polynesia
USNM	361384	Kure Atoll	Male	Polynesia
USNM	361385	Kure Atoll	Male	Polynesia
USNM	361386	Kure Atoll	Male	Polynesia
USNM	361387	Kure Atoll	Male	Polynesia
USNM	355758	Kure Atoll	Male	Polynesia
USNM	355759	Kure Atoll	Male	Polynesia
USNM	355762	Kure Atoll	Male	Polynesia
USNM	355763	Kure Atoll	Male	Polynesia
USNM	355764	Kure Atoll	Male	Polynesia
USNM	355765	Kure Atoll	Male	Polynesia
USNM	355767	Kure Atoll	Male	Polynesia
USNM	355768	Kure Atoll	Male	Polynesia
USNM	355769	Kure Atoll	Male	Polynesia
USNM	355770	Kure Atoll	Male	Polynesia
USNM	355877	Kure Atoll	Male	Polynesia
USNM	355882	Kure Atoll	Male	Polynesia
USNM	355885	Kure Atoll	Male	Polynesia
USNM	361426	Kure Atoll	Male	Polynesia
USNM	288022	Kure Atoll	Unknown	Polynesia
USNM	361414	Manra Island	Female	Polynesia
USNM	257023	Maui	Female	Polynesia
USNM	257926	Maui	Female	Polynesia
USNM	257928	Maui	Female	Polynesia
USNM	257930	Maui	Female	Polynesia
USNM	257931	Maui	Female	Polynesia
USNM	257933	Maui	Female	Polynesia
USNM	257934	Maui	Female	Polynesia
USNM	257935	Maui	Male	Polynesia
USNM	257022	Maui	Male	Polynesia
USNM	257925	Maui	Male	Polynesia
USNM	257927	Maui	Male	Polynesia
USNM	257929	Maui	Male	Polynesia
USNM	257932	Maui	Male	Polynesia
USNM	360996	Nukunonu Atoll	Female	Polynesia
USNM	360995	Nukunonu Atoll	Male	Polynesia
USNM	240915	Oahu	Female	Polynesia
USNM	257953	Oahu	Female	Polynesia
USNM	257954	Oahu	Female	Polynesia
USNM	257955	Oahu	Female	Polynesia
USNM	257956	Oahu	Female	Polynesia
USNM	257959	Oahu	Female	Polynesia
USNM	236897	Oahu	Female	Polynesia
USNM	236898	Oahu	Female	Polynesia
USNM	236899	Oahu	Female	Polynesia
USNM	240917	Oahu	Female	Polynesia

Institute	Specimen	Island	Sex	Biogeographical region
USNM	257949	Oahu	Female	Polynesia
USNM	257110	Oahu	Female	Polynesia
USNM	257111	Oahu	Female	Polynesia
USNM	243905	Oahu	Male	Polynesia
USNM	361467	Oahu	Male	Polynesia
USNM	257952	Oahu	Male	Polynesia
USNM	257957	Oahu	Male	Polynesia
USNM	257958	Oahu	Male	Polynesia
USNM	236896	Oahu	Male	Polynesia
USNM	236900	Oahu	Male	Polynesia
USNM	236901	Oahu	Male	Polynesia
USNM	240916	Oahu	Male	Polynesia
USNM	257948	Oahu	Male	Polynesia
USNM	257950	Oahu	Male	Polynesia
USNM	257109	Oahu	Male	Polynesia
USNM	243906	Oahu	Male	Polynesia
USNM	257951	Oahu	Unknown	Polynesia
USNM	294551	Onotoa Atoll	Female	Polynesia
USNM	294547	Onotoa Atoll	Male	Polynesia
USNM	294549	Onotoa Atoll	Male	Polynesia
USNM	294550	Onotoa Atoll	Male	Polynesia
USNM	294548	Onotoa Atoll	Unknown	Polynesia
USNM	361430	Orona Atoll	Male	Polynesia
USNM	269148	Orona Atoll	Unknown	Polynesia
USNM	294991	Raroia Atoll	Female	Polynesia
USNM	294994	Raroia Atoll	Female	Polynesia
USNM	294995	Raroia Atoll	Female	Polynesia
USNM	294996	Raroia Atoll	Female	Polynesia
USNM	294992	Raroia Atoll	Male	Polynesia
USNM	294993	Raroia Atoll	Male	Polynesia
USNM	294997	Raroia Atoll	Male	Polynesia
USNM	532898	Rose Atoll	Female	Polynesia
USNM	532900	Rose Atoll	Female	Polynesia
USNM	532897	Rose Atoll	Male	Polynesia
USNM	532895	Rose Atoll	Male	Polynesia
USNM	532896	Rose Atoll	Male	Polynesia
USNM	532899	Rose Atoll	Male	Polynesia
USNM	362194	Swains Island	Female	Polynesia
USNM	362197	Swains Island	Female	Polynesia
USNM	362192	Swains Island	Female	Polynesia
USNM	362195	Swains Island	Female	Polynesia
USNM	362198	Swains Island	Female	Polynesia
USNM	362199	Swains Island	Female	Polynesia
USNM	362201	Swains Island	Female	Polynesia
USNM	362202	Swains Island	Female	Polynesia
USNM	362193	Swains Island	Male	Polynesia
USNM	362196	Swains Island	Male	Polynesia
USNM	362200	Swains Island	Male	Polynesia
USNM	362203	Swains Island	Male	Polynesia
USNM	3730	Tahiti	Unknown	Polynesia
USNM	256168	Niuafo'Ou Island	Female	Polynesia
USNM	256162	Niuafo'Ou Island	Male	Polynesia
USNM	256169	Niuafo'Ou Island	Male	Polynesia
USNM	8605	Tuamotu Archipelag	Female	Polynesia
USNM	361438	Vostok Island	Female	Polynesia
USNM	361441	Vostok Island	Female	Polynesia
USNM	361443	Vostok Island	Female	Polynesia
USNM	361444	Vostok Island	Female	Polynesia
USNM	361449	Vostok Island	Female	Polynesia
USNM	361442	Vostok Island	Male	Polynesia
USNM	361445	Vostok Island	Male	Polynesia
USNM	361446	Vostok Island	Male	Polynesia
USNM	361447	Vostok Island	Male	Polynesia
USNM	361448	Vostok Island	Male	Polynesia
USNM	478039	Palawan	Female	Sunda
USNM	478040	Palawan	Female	Sunda

Institute	Specimen	Island	Sex	Biogeographical region
USNM	478042	Palawan	Female	Sunda
USNM	478043	Palawan	Female	Sunda
USNM	478050	Palawan	Female	Sunda
USNM	478063	Palawan	Female	Sunda
USNM	478073	Palawan	Female	Sunda
USNM	478041	Palawan	Female	Sunda
USNM	478013	Palawan	Female	Sunda
USNM	478014	Palawan	Female	Sunda
USNM	478015	Palawan	Female	Sunda
USNM	478017	Palawan	Female	Sunda
USNM	478021	Palawan	Female	Sunda
USNM	478022	Palawan	Female	Sunda
USNM	478023	Palawan	Female	Sunda
USNM	478026	Palawan	Female	Sunda
USNM	478028	Palawan	Female	Sunda
USNM	478031	Palawan	Female	Sunda
USNM	478032	Palawan	Female	Sunda
USNM	478033	Palawan	Female	Sunda
USNM	478047	Palawan	Female	Sunda
USNM	478053	Palawan	Female	Sunda
USNM	478054	Palawan	Female	Sunda
USNM	478055	Palawan	Female	Sunda
USNM	478056	Palawan	Female	Sunda
USNM	478058	Palawan	Female	Sunda
USNM	478062	Palawan	Female	Sunda
USNM	478068	Palawan	Female	Sunda
USNM	478074	Palawan	Female	Sunda
USNM	478075	Palawan	Female	Sunda
USNM	478078	Palawan	Female	Sunda
USNM	478080	Palawan	Female	Sunda
USNM	478082	Palawan	Female	Sunda
USNM	478083	Palawan	Female	Sunda
USNM	478085	Palawan	Female	Sunda
USNM	478048	Palawan	Male	Sunda
USNM	478049	Palawan	Male	Sunda
USNM	478077	Palawan	Male	Sunda
USNM	348619	Palawan	Male	Sunda
USNM	478011	Palawan	Male	Sunda
USNM	478012	Palawan	Male	Sunda
USNM	478016	Palawan	Male	Sunda
USNM	478019	Palawan	Male	Sunda
USNM	478020	Palawan	Male	Sunda
USNM	478024	Palawan	Male	Sunda
USNM	478025	Palawan	Male	Sunda
USNM	478030	Palawan	Male	Sunda
USNM	478034	Palawan	Male	Sunda
USNM	478035	Palawan	Male	Sunda
USNM	478036	Palawan	Male	Sunda
USNM	478037	Palawan	Male	Sunda
USNM	478038	Palawan	Male	Sunda
USNM	478044	Palawan	Male	Sunda
USNM	478045	Palawan	Male	Sunda
USNM	478046	Palawan	Male	Sunda
USNM	478051	Palawan	Male	Sunda
USNM	478052	Palawan	Male	Sunda
USNM	478057	Palawan	Male	Sunda
USNM	478059	Palawan	Male	Sunda
USNM	478060	Palawan	Male	Sunda
USNM	478061	Palawan	Male	Sunda
USNM	478067	Palawan	Male	Sunda
USNM	478069	Palawan	Male	Sunda
USNM	478070	Palawan	Male	Sunda
USNM	478071	Palawan	Male	Sunda
USNM	478072	Palawan	Male	Sunda
USNM	478079	Palawan	Male	Sunda
USNM	478081	Palawan	Male	Sunda

Institute	Specimen	Island	Sex	Biogeographical region
USNM	478084	Palawan	Male	Sunda
USNM	478086	Palawan	Male	Sunda
USNM	478087	Palawan	Male	Sunda
USNM	478088	Palawan	Male	Sunda
USNM	478027	Palawan	Unknown	Sunda
USNM	478076	Palawan	Unknown	Sunda
USNM	478018	Palawan	Unknown	Sunda
USNM	478029	Palawan	Unknown	Sunda
USNM	144635	Basilan Island	Male	Wallacea
USNM	144634	Basilan Island	Male	Wallacea
USNM	144636	Basilan Island	Male	Wallacea
USNM	478090	Busuanga Island	Female	Wallacea
USNM	478091	Busuanga Island	Female	Wallacea
USNM	478094	Busuanga Island	Female	Wallacea
USNM	478095	Busuanga Island	Female	Wallacea
USNM	478096	Busuanga Island	Female	Wallacea
USNM	478099	Busuanga Island	Female	Wallacea
USNM	478100	Busuanga Island	Female	Wallacea
USNM	478103	Busuanga Island	Female	Wallacea
USNM	478089	Busuanga Island	Male	Wallacea
USNM	478092	Busuanga Island	Male	Wallacea
USNM	478093	Busuanga Island	Male	Wallacea
USNM	478097	Busuanga Island	Male	Wallacea
USNM	478098	Busuanga Island	Male	Wallacea
USNM	478101	Busuanga Island	Male	Wallacea
USNM	478102	Busuanga Island	Male	Wallacea
USNM	478104	Busuanga Island	Male	Wallacea
USNM	478105	Busuanga Island	Male	Wallacea
USNM	155144	Catanduanes Island	Female	Wallacea
USNM	219702	Celebes	Female	Wallacea
USNM	219697	Celebes	Female	Wallacea
USNM	217797	Celebes	Female	Wallacea
USNM	217798	Celebes	Female	Wallacea
USNM	217800	Celebes	Female	Wallacea
USNM	217722	Celebes	Female	Wallacea
USNM	217723	Celebes	Female	Wallacea
USNM	199911	Celebes	Female	Wallacea
USNM	199913	Celebes	Female	Wallacea
USNM	199919	Celebes	Female	Wallacea
USNM	199920	Celebes	Female	Wallacea
USNM	199921	Celebes	Female	Wallacea
USNM	199922	Celebes	Female	Wallacea
USNM	199931	Celebes	Female	Wallacea
USNM	199932	Celebes	Female	Wallacea
USNM	199933	Celebes	Female	Wallacea
USNM	216954	Celebes	Female	Wallacea
USNM	217796	Celebes	Female	Wallacea
USNM	257626	Celebes	Female	Wallacea
USNM	199974	Celebes	Female	Wallacea
USNM	199977	Celebes	Female	Wallacea
USNM	199978	Celebes	Female	Wallacea
USNM	199979	Celebes	Female	Wallacea
USNM	199980	Celebes	Female	Wallacea
USNM	199981	Celebes	Female	Wallacea
USNM	199982	Celebes	Female	Wallacea
USNM	199993	Celebes	Female	Wallacea
USNM	199994	Celebes	Female	Wallacea
USNM	199995	Celebes	Female	Wallacea
USNM	199956	Celebes	Female	Wallacea
USNM	199960	Celebes	Female	Wallacea
USNM	199965	Celebes	Female	Wallacea
USNM	199969	Celebes	Female	Wallacea
USNM	199938	Celebes	Female	Wallacea
USNM	199949	Celebes	Female	Wallacea
USNM	200016	Celebes	Female	Wallacea
USNM	200017	Celebes	Female	Wallacea

Institute	Specimen	Island	Sex	Biogeographical region
USNM	200018	Celebes	Female	Wallacea
USNM	200000	Celebes	Female	Wallacea
USNM	200003	Celebes	Female	Wallacea
USNM	200004	Celebes	Female	Wallacea
USNM	219696	Celebes	Female	Wallacea
USNM	219698	Celebes	Female	Wallacea
USNM	219700	Celebes	Female	Wallacea
USNM	496913	Celebes	Female	Wallacea
USNM	496914	Celebes	Female	Wallacea
USNM	496917	Celebes	Female	Wallacea
USNM	496918	Celebes	Female	Wallacea
USNM	496919	Celebes	Female	Wallacea
USNM	496920	Celebes	Female	Wallacea
USNM	496922	Celebes	Female	Wallacea
USNM	496923	Celebes	Female	Wallacea
USNM	496925	Celebes	Female	Wallacea
USNM	496926	Celebes	Female	Wallacea
USNM	496927	Celebes	Female	Wallacea
USNM	496929	Celebes	Female	Wallacea
USNM	496930	Celebes	Female	Wallacea
USNM	496932	Celebes	Female	Wallacea
USNM	496933	Celebes	Female	Wallacea
USNM	496937	Celebes	Female	Wallacea
USNM	496938	Celebes	Female	Wallacea
USNM	496939	Celebes	Female	Wallacea
USNM	496940	Celebes	Female	Wallacea
USNM	496943	Celebes	Female	Wallacea
USNM	496944	Celebes	Female	Wallacea
USNM	496946	Celebes	Female	Wallacea
USNM	496952	Celebes	Female	Wallacea
USNM	496954	Celebes	Female	Wallacea
USNM	496956	Celebes	Female	Wallacea
USNM	496957	Celebes	Female	Wallacea
USNM	496958	Celebes	Female	Wallacea
USNM	496960	Celebes	Female	Wallacea
USNM	496961	Celebes	Female	Wallacea
USNM	496964	Celebes	Female	Wallacea
USNM	257624	Celebes	Female	Wallacea
USNM	199984	Celebes	Female	Wallacea
USNM	217799	Celebes	Male	Wallacea
USNM	217801	Celebes	Male	Wallacea
USNM	199912	Celebes	Male	Wallacea
USNM	199914	Celebes	Male	Wallacea
USNM	199915	Celebes	Male	Wallacea
USNM	199917	Celebes	Male	Wallacea
USNM	199923	Celebes	Male	Wallacea
USNM	199924	Celebes	Male	Wallacea
USNM	199925	Celebes	Male	Wallacea
USNM	199928	Celebes	Male	Wallacea
USNM	199929	Celebes	Male	Wallacea
USNM	199934	Celebes	Male	Wallacea
USNM	216965	Celebes	Male	Wallacea
USNM	216966	Celebes	Male	Wallacea
USNM	199975	Celebes	Male	Wallacea
USNM	199983	Celebes	Male	Wallacea
USNM	199985	Celebes	Male	Wallacea
USNM	199986	Celebes	Male	Wallacea
USNM	199988	Celebes	Male	Wallacea
USNM	199989	Celebes	Male	Wallacea
USNM	199990	Celebes	Male	Wallacea
USNM	199955	Celebes	Male	Wallacea
USNM	199958	Celebes	Male	Wallacea
USNM	199959	Celebes	Male	Wallacea
USNM	199961	Celebes	Male	Wallacea
USNM	199962	Celebes	Male	Wallacea
USNM	199963	Celebes	Male	Wallacea

Institute	Specimen	Island	Sex	Biogeographical region
USNM	199964	Celebes	Male	Wallacea
USNM	199966	Celebes	Male	Wallacea
USNM	199967	Celebes	Male	Wallacea
USNM	199968	Celebes	Male	Wallacea
USNM	199970	Celebes	Male	Wallacea
USNM	199971	Celebes	Male	Wallacea
USNM	199972	Celebes	Male	Wallacea
USNM	199973	Celebes	Male	Wallacea
USNM	199935	Celebes	Male	Wallacea
USNM	199936	Celebes	Male	Wallacea
USNM	199937	Celebes	Male	Wallacea
USNM	199939	Celebes	Male	Wallacea
USNM	199940	Celebes	Male	Wallacea
USNM	199941	Celebes	Male	Wallacea
USNM	199942	Celebes	Male	Wallacea
USNM	199943	Celebes	Male	Wallacea
USNM	199944	Celebes	Male	Wallacea
USNM	199945	Celebes	Male	Wallacea
USNM	199946	Celebes	Male	Wallacea
USNM	199947	Celebes	Male	Wallacea
USNM	199948	Celebes	Male	Wallacea
USNM	199950	Celebes	Male	Wallacea
USNM	199951	Celebes	Male	Wallacea
USNM	199954	Celebes	Male	Wallacea
USNM	200014	Celebes	Male	Wallacea
USNM	200015	Celebes	Male	Wallacea
USNM	199996	Celebes	Male	Wallacea
USNM	199997	Celebes	Male	Wallacea
USNM	199998	Celebes	Male	Wallacea
USNM	199999	Celebes	Male	Wallacea
USNM	200001	Celebes	Male	Wallacea
USNM	200002	Celebes	Male	Wallacea
USNM	200005	Celebes	Male	Wallacea
USNM	200006	Celebes	Male	Wallacea
USNM	200007	Celebes	Male	Wallacea
USNM	200008	Celebes	Male	Wallacea
USNM	200009	Celebes	Male	Wallacea
USNM	200010	Celebes	Male	Wallacea
USNM	200011	Celebes	Male	Wallacea
USNM	200012	Celebes	Male	Wallacea
USNM	200013	Celebes	Male	Wallacea
USNM	219693	Celebes	Male	Wallacea
USNM	219694	Celebes	Male	Wallacea
USNM	219695	Celebes	Male	Wallacea
USNM	219701	Celebes	Male	Wallacea
USNM	219699	Celebes	Male	Wallacea
USNM	199976	Celebes	Male	Wallacea
USNM	496924	Celebes	Male	Wallacea
USNM	496928	Celebes	Male	Wallacea
USNM	496931	Celebes	Male	Wallacea
USNM	496934	Celebes	Male	Wallacea
USNM	496935	Celebes	Male	Wallacea
USNM	496936	Celebes	Male	Wallacea
USNM	496941	Celebes	Male	Wallacea
USNM	496942	Celebes	Male	Wallacea
USNM	496945	Celebes	Male	Wallacea
USNM	496947	Celebes	Male	Wallacea
USNM	496948	Celebes	Male	Wallacea
USNM	496949	Celebes	Male	Wallacea
USNM	496950	Celebes	Male	Wallacea
USNM	496953	Celebes	Male	Wallacea
USNM	496955	Celebes	Male	Wallacea
USNM	496959	Celebes	Male	Wallacea
USNM	496962	Celebes	Male	Wallacea
USNM	257625	Celebes	Male	Wallacea
USNM	496912	Celebes	Male	Wallacea

Institute	Specimen	Island	Sex	Biogeographical region
USNM	496915	Celebes	Male	Wallacea
USNM	496916	Celebes	Male	Wallacea
USNM	496921	Celebes	Male	Wallacea
USNM	478107	Culion Island	Female	Wallacea
USNM	478108	Culion Island	Female	Wallacea
USNM	478110	Culion Island	Female	Wallacea
USNM	478106	Culion Island	Male	Wallacea
USNM	478109	Culion Island	Male	Wallacea
USNM	277414	Leyte Island	Female	Wallacea
USNM	277692	Leyte Island	Unknown	Wallacea
USNM	145848	Luzon	Female	Wallacea
USNM	277697	Luzon	Female	Wallacea
USNM	289916	Luzon	Female	Wallacea
USNM	348572	Luzon	Female	Wallacea
USNM	348599	Luzon	Female	Wallacea
USNM	348600	Luzon	Female	Wallacea
USNM	348603	Luzon	Female	Wallacea
USNM	348604	Luzon	Female	Wallacea
USNM	348605	Luzon	Female	Wallacea
USNM	348607	Luzon	Female	Wallacea
USNM	348608	Luzon	Female	Wallacea
USNM	348609	Luzon	Female	Wallacea
USNM	348612	Luzon	Female	Wallacea
USNM	348614	Luzon	Female	Wallacea
USNM	348618	Luzon	Female	Wallacea
USNM	348621	Luzon	Female	Wallacea
USNM	348622	Luzon	Female	Wallacea
USNM	348624	Luzon	Female	Wallacea
USNM	348626	Luzon	Female	Wallacea
USNM	145806	Luzon	Female	Wallacea
USNM	145811	Luzon	Female	Wallacea
USNM	145814	Luzon	Female	Wallacea
USNM	145818	Luzon	Female	Wallacea
USNM	145819	Luzon	Female	Wallacea
USNM	145820	Luzon	Female	Wallacea
USNM	145821	Luzon	Female	Wallacea
USNM	145822	Luzon	Female	Wallacea
USNM	145826	Luzon	Female	Wallacea
USNM	145827	Luzon	Female	Wallacea
USNM	145828	Luzon	Female	Wallacea
USNM	145829	Luzon	Female	Wallacea
USNM	145830	Luzon	Female	Wallacea
USNM	145832	Luzon	Female	Wallacea
USNM	145834	Luzon	Female	Wallacea
USNM	145836	Luzon	Female	Wallacea
USNM	145837	Luzon	Female	Wallacea
USNM	145841	Luzon	Female	Wallacea
USNM	145842	Luzon	Female	Wallacea
USNM	145843	Luzon	Female	Wallacea
USNM	145846	Luzon	Female	Wallacea
USNM	151491	Luzon	Female	Wallacea
USNM	151492	Luzon	Female	Wallacea
USNM	151497	Luzon	Female	Wallacea
USNM	151498	Luzon	Female	Wallacea
USNM	151499	Luzon	Female	Wallacea
USNM	277903	Luzon	Female	Wallacea
USNM	277904	Luzon	Female	Wallacea
USNM	277905	Luzon	Female	Wallacea
USNM	277906	Luzon	Female	Wallacea
USNM	277908	Luzon	Female	Wallacea
USNM	277699	Luzon	Female	Wallacea
USNM	278014	Luzon	Female	Wallacea
USNM	278015	Luzon	Female	Wallacea
USNM	278017	Luzon	Female	Wallacea
USNM	278018	Luzon	Female	Wallacea
USNM	278458	Luzon	Female	Wallacea

Institute	Specimen	Island	Sex	Biogeographical region
USNM	278459	Luzon	Female	Wallacea
USNM	278461	Luzon	Female	Wallacea
USNM	278462	Luzon	Female	Wallacea
USNM	278463	Luzon	Female	Wallacea
USNM	279096	Luzon	Female	Wallacea
USNM	279097	Luzon	Female	Wallacea
USNM	279098	Luzon	Female	Wallacea
USNM	278570	Luzon	Female	Wallacea
USNM	278577	Luzon	Female	Wallacea
USNM	278580	Luzon	Female	Wallacea
USNM	278582	Luzon	Female	Wallacea
USNM	278583	Luzon	Female	Wallacea
USNM	278625	Luzon	Female	Wallacea
USNM	278626	Luzon	Female	Wallacea
USNM	279078	Luzon	Female	Wallacea
USNM	279081	Luzon	Female	Wallacea
USNM	279083	Luzon	Female	Wallacea
USNM	279085	Luzon	Female	Wallacea
USNM	279086	Luzon	Female	Wallacea
USNM	279088	Luzon	Female	Wallacea
USNM	279089	Luzon	Female	Wallacea
USNM	279090	Luzon	Female	Wallacea
USNM	261171	Luzon	Female	Wallacea
USNM	261172	Luzon	Female	Wallacea
USNM	304296	Luzon	Female	Wallacea
USNM	287427	Luzon	Female	Wallacea
USNM	287428	Luzon	Female	Wallacea
USNM	278154	Luzon	Female	Wallacea
USNM	279608	Luzon	Female	Wallacea
USNM	279609	Luzon	Female	Wallacea
USNM	279610	Luzon	Female	Wallacea
USNM	356633	Luzon	Female	Wallacea
USNM	356634	Luzon	Female	Wallacea
USNM	356635	Luzon	Female	Wallacea
USNM	356637	Luzon	Female	Wallacea
USNM	356640	Luzon	Female	Wallacea
USNM	356641	Luzon	Female	Wallacea
USNM	283840	Luzon	Female	Wallacea
USNM	283842	Luzon	Female	Wallacea
USNM	348576	Luzon	Female	Wallacea
USNM	348577	Luzon	Female	Wallacea
USNM	348623	Luzon	Female	Wallacea
USNM	536767	Luzon	Female	Wallacea
USNM	277703	Luzon	Male	Wallacea
USNM	144600	Luzon	Male	Wallacea
USNM	145771	Luzon	Male	Wallacea
USNM	348573	Luzon	Male	Wallacea
USNM	348574	Luzon	Male	Wallacea
USNM	348579	Luzon	Male	Wallacea
USNM	348601	Luzon	Male	Wallacea
USNM	348606	Luzon	Male	Wallacea
USNM	348610	Luzon	Male	Wallacea
USNM	348611	Luzon	Male	Wallacea
USNM	348613	Luzon	Male	Wallacea
USNM	348615	Luzon	Male	Wallacea
USNM	348616	Luzon	Male	Wallacea
USNM	348617	Luzon	Male	Wallacea
USNM	348620	Luzon	Male	Wallacea
USNM	348625	Luzon	Male	Wallacea
USNM	145804	Luzon	Male	Wallacea
USNM	145805	Luzon	Male	Wallacea
USNM	145807	Luzon	Male	Wallacea
USNM	145812	Luzon	Male	Wallacea
USNM	145813	Luzon	Male	Wallacea
USNM	145815	Luzon	Male	Wallacea
USNM	145816	Luzon	Male	Wallacea

Institute	Specimen	Island	Sex	Biogeographical region
USNM	145817	Luzon	Male	Wallacea
USNM	145823	Luzon	Male	Wallacea
USNM	145824	Luzon	Male	Wallacea
USNM	145831	Luzon	Male	Wallacea
USNM	145835	Luzon	Male	Wallacea
USNM	145838	Luzon	Male	Wallacea
USNM	145839	Luzon	Male	Wallacea
USNM	145840	Luzon	Male	Wallacea
USNM	145844	Luzon	Male	Wallacea
USNM	145845	Luzon	Male	Wallacea
USNM	145849	Luzon	Male	Wallacea
USNM	145850	Luzon	Male	Wallacea
USNM	151493	Luzon	Male	Wallacea
USNM	151494	Luzon	Male	Wallacea
USNM	151495	Luzon	Male	Wallacea
USNM	277695	Luzon	Male	Wallacea
USNM	277907	Luzon	Male	Wallacea
USNM	277698	Luzon	Male	Wallacea
USNM	277700	Luzon	Male	Wallacea
USNM	277701	Luzon	Male	Wallacea
USNM	278010	Luzon	Male	Wallacea
USNM	278011	Luzon	Male	Wallacea
USNM	278012	Luzon	Male	Wallacea
USNM	278013	Luzon	Male	Wallacea
USNM	278016	Luzon	Male	Wallacea
USNM	278460	Luzon	Male	Wallacea
USNM	278464	Luzon	Male	Wallacea
USNM	278564	Luzon	Male	Wallacea
USNM	278565	Luzon	Male	Wallacea
USNM	278566	Luzon	Male	Wallacea
USNM	278567	Luzon	Male	Wallacea
USNM	278568	Luzon	Male	Wallacea
USNM	279099	Luzon	Male	Wallacea
USNM	279100	Luzon	Male	Wallacea
USNM	279101	Luzon	Male	Wallacea
USNM	278569	Luzon	Male	Wallacea
USNM	278575	Luzon	Male	Wallacea
USNM	278578	Luzon	Male	Wallacea
USNM	278579	Luzon	Male	Wallacea
USNM	278581	Luzon	Male	Wallacea
USNM	278624	Luzon	Male	Wallacea
USNM	279079	Luzon	Male	Wallacea
USNM	279080	Luzon	Male	Wallacea
USNM	279082	Luzon	Male	Wallacea
USNM	279084	Luzon	Male	Wallacea
USNM	279087	Luzon	Male	Wallacea
USNM	279091	Luzon	Male	Wallacea
USNM	279092	Luzon	Male	Wallacea
USNM	279093	Luzon	Male	Wallacea
USNM	279094	Luzon	Male	Wallacea
USNM	279095	Luzon	Male	Wallacea
USNM	261173	Luzon	Male	Wallacea
USNM	261174	Luzon	Male	Wallacea
USNM	261175	Luzon	Male	Wallacea
USNM	261179	Luzon	Male	Wallacea
USNM	287426	Luzon	Male	Wallacea
USNM	304293	Luzon	Male	Wallacea
USNM	304294	Luzon	Male	Wallacea
USNM	304295	Luzon	Male	Wallacea
USNM	304297	Luzon	Male	Wallacea
USNM	304298	Luzon	Male	Wallacea
USNM	304299	Luzon	Male	Wallacea
USNM	304300	Luzon	Male	Wallacea
USNM	278153	Luzon	Male	Wallacea
USNM	279607	Luzon	Male	Wallacea
USNM	356636	Luzon	Male	Wallacea

Institute	Specimen	Island	Sex	Biogeographical region
USNM	356639	Luzon	Male	Wallacea
USNM	356642	Luzon	Male	Wallacea
USNM	283837	Luzon	Male	Wallacea
USNM	283838	Luzon	Male	Wallacea
USNM	283839	Luzon	Male	Wallacea
USNM	283841	Luzon	Male	Wallacea
USNM	145833	Luzon	Male	Wallacea
USNM	348575	Luzon	Male	Wallacea
USNM	348578	Luzon	Male	Wallacea
USNM	348580	Luzon	Male	Wallacea
USNM	348627	Luzon	Male	Wallacea
USNM	348628	Luzon	Male	Wallacea
USNM	277690	Luzon	Unknown	Wallacea
USNM	174861	Luzon	Unknown	Wallacea
USNM	174862	Luzon	Unknown	Wallacea
USNM	277691	Luzon	Unknown	Wallacea
USNM	277693	Luzon	Unknown	Wallacea
USNM	277694	Luzon	Unknown	Wallacea
USNM	277696	Luzon	Unknown	Wallacea
USNM	277702	Luzon	Unknown	Wallacea
USNM	287425	Luzon	Unknown	Wallacea
USNM	143636	Mindanao	Adult	Wallacea
USNM	143635	Mindanao	Female	Wallacea
USNM	145769	Mindanao	Female	Wallacea
USNM	145773	Mindanao	Female	Wallacea
USNM	145780	Mindanao	Female	Wallacea
USNM	145781	Mindanao	Female	Wallacea
USNM	145782	Mindanao	Female	Wallacea
USNM	145787	Mindanao	Female	Wallacea
USNM	125218	Mindanao	Female	Wallacea
USNM	125220	Mindanao	Female	Wallacea
USNM	125221	Mindanao	Female	Wallacea
USNM	125222	Mindanao	Female	Wallacea
USNM	144613	Mindanao	Female	Wallacea
USNM	125223	Mindanao	Female	Wallacea
USNM	123294	Mindanao	Female	Wallacea
USNM	125216	Mindanao	Female	Wallacea
USNM	125224	Mindanao	Female	Wallacea
USNM	462219	Mindanao	Female	Wallacea
USNM	462220	Mindanao	Female	Wallacea
USNM	462221	Mindanao	Female	Wallacea
USNM	462222	Mindanao	Female	Wallacea
USNM	462223	Mindanao	Female	Wallacea
USNM	462224	Mindanao	Female	Wallacea
USNM	125229	Mindanao	Female	Wallacea
USNM	145778	Mindanao	Male	Wallacea
USNM	145775	Mindanao	Male	Wallacea
USNM	261168	Mindanao	Male	Wallacea
USNM	261169	Mindanao	Male	Wallacea
USNM	125219	Mindanao	Male	Wallacea
USNM	144614	Mindanao	Male	Wallacea
USNM	261176	Mindanao	Male	Wallacea
USNM	462209	Mindanao	Male	Wallacea
USNM	462214	Mindanao	Male	Wallacea
USNM	462215	Mindanao	Male	Wallacea
USNM	462216	Mindanao	Male	Wallacea
USNM	462217	Mindanao	Male	Wallacea
USNM	462218	Mindanao	Male	Wallacea
USNM	125225	Mindanao	Male	Wallacea
USNM	125227	Mindanao	Male	Wallacea
USNM	145786	Mindanao	Unknown	Wallacea
USNM	145791	Mindanao	Unknown	Wallacea
USNM	145785	Mindanao	Unknown	Wallacea
USNM	277633	Mindoro	Female	Wallacea
USNM	144960	Mindoro	Female	Wallacea
USNM	348602	Mindoro	Female	Wallacea

Institute	Specimen	Island	Sex	Biogeographical region
USNM	277656	Mindoro	Female	Wallacea
USNM	277658	Mindoro	Female	Wallacea
USNM	277659	Mindoro	Female	Wallacea
USNM	277660	Mindoro	Female	Wallacea
USNM	278587	Mindoro	Female	Wallacea
USNM	278590	Mindoro	Female	Wallacea
USNM	278594	Mindoro	Female	Wallacea
USNM	283844	Mindoro	Female	Wallacea
USNM	277585	Mindoro	Female	Wallacea
USNM	277586	Mindoro	Female	Wallacea
USNM	277640	Mindoro	Female	Wallacea
USNM	277643	Mindoro	Female	Wallacea
USNM	277645	Mindoro	Female	Wallacea
USNM	277646	Mindoro	Female	Wallacea
USNM	277647	Mindoro	Female	Wallacea
USNM	277650	Mindoro	Female	Wallacea
USNM	277655	Mindoro	Female	Wallacea
USNM	144638	Mindoro	Male	Wallacea
USNM	144961	Mindoro	Male	Wallacea
USNM	277657	Mindoro	Male	Wallacea
USNM	278585	Mindoro	Male	Wallacea
USNM	278586	Mindoro	Male	Wallacea
USNM	278589	Mindoro	Male	Wallacea
USNM	278591	Mindoro	Male	Wallacea
USNM	278593	Mindoro	Male	Wallacea
USNM	283843	Mindoro	Male	Wallacea
USNM	144639	Mindoro	Male	Wallacea
USNM	277583	Mindoro	Male	Wallacea
USNM	277584	Mindoro	Male	Wallacea
USNM	277641	Mindoro	Male	Wallacea
USNM	277642	Mindoro	Male	Wallacea
USNM	277644	Mindoro	Male	Wallacea
USNM	277648	Mindoro	Male	Wallacea
USNM	277649	Mindoro	Male	Wallacea
USNM	277651	Mindoro	Male	Wallacea
USNM	277652	Mindoro	Male	Wallacea
USNM	277653	Mindoro	Male	Wallacea
USNM	277654	Mindoro	Male	Wallacea
USNM	144637	Mindoro	Male	Wallacea
USNM	277554	Mindoro	Unknown	Wallacea
USNM	277555	Mindoro	Unknown	Wallacea
USNM	458832	Negros	Female	Wallacea
USNM	458834	Negros	Female	Wallacea
USNM	458837	Negros	Female	Wallacea
USNM	458841	Negros	Female	Wallacea
USNM	458825	Negros	Female	Wallacea
USNM	458828	Negros	Female	Wallacea
USNM	458842	Negros	Male	Wallacea
USNM	458835	Negros	Male	Wallacea
USNM	458836	Negros	Male	Wallacea
USNM	458839	Negros	Male	Wallacea
USNM	458840	Negros	Male	Wallacea
USNM	458843	Negros	Male	Wallacea
USNM	458826	Negros	Male	Wallacea
USNM	458827	Negros	Male	Wallacea
USNM	458829	Negros	Male	Wallacea
USNM	458831	Negros	Male	Wallacea
USNM	458833	Negros	Male	Wallacea
USNM	458838	Negros	Male	Wallacea
USNM	458844	Negros	Male	Wallacea
USNM	239244	Negros	Unknown	Wallacea
USNM	239243	Negros	Unknown	Wallacea
USNM	261165	Panay	Female	Wallacea
USNM	261167	Panay	Female	Wallacea
USNM	261181	Panay	Female	Wallacea
USNM	261180	Panay	Female	Wallacea

Institute	Specimen	Island	Sex	Biogeographical region
USNM	261166	Panay	Male	Wallacea
USNM	278131	Samar	Female	Wallacea
USNM	278132	Samar	Female	Wallacea
USNM	278136	Samar	Female	Wallacea
USNM	278137	Samar	Female	Wallacea
USNM	278130	Samar	Male	Wallacea
USNM	278133	Samar	Male	Wallacea
USNM	278134	Samar	Male	Wallacea
USNM	278135	Samar	Male	Wallacea
USNM	199927	Sulawesi	Male	Wallacea

Institute	Specimen	Island	Sex	Biogeographical region
NMNZ	337	Cuvier Island (Repanga)	male	Polynesia
NMNZ	338	Cuvier Island (Repanga)	male	Polynesia
NMNZ	339	Cuvier Island (Repanga)	male	Polynesia
NMNZ	340	Cuvier Island (Repanga)	female	Polynesia
NMNZ	341	Cuvier Island (Repanga)	male	Polynesia
NMNZ	342	Cuvier Island (Repanga)	female	Polynesia
NMNZ	n.n.	Cuvier Island (Repanga)	male	Polynesia
NMNZ	AED119	Hen Island	male	Polynesia
NMNZ	AED375	Hen Island	male	Polynesia
NMNZ	AED376	Hen Island	female	Polynesia
NMNZ	AED377	Hen Island	male	Polynesia
NMNZ	AED205	Little Barrier	male	Polynesia
NMNZ	AED238	Little Barrier	female	Polynesia
NMNZ	AED241	Little Barrier	male	Polynesia
NMNZ	AED490	Little Barrier	male	Polynesia
NMNZ	AED491	Little Barrier	female	Polynesia
NMNZ	AED492	Little Barrier	female	Polynesia
NMNZ	AED493	Little Barrier	male	Polynesia
NMNZ	AED494	Little Barrier	male	Polynesia
NMNZ	AED495	Little Barrier	female	Polynesia
NMNZ	AED496	Little Barrier	male	Polynesia
NMNZ	AED497	Little Barrier	female	Polynesia
NMNZ	AED498	Little Barrier	male	Polynesia
NMNZ	AED499	Little Barrier	female	Polynesia
NMNZ	AED501	Little Barrier	female	Polynesia
NMNZ	LB5	Little Barrier	male	Polynesia
NMNZ	LB11	Little Barrier	male	Polynesia
NMNZ	LB22	Little Barrier	female	Polynesia
NMNZ	LB43	Little Barrier	female	Polynesia
NMNZ	LB17	Little Barrier	female	Polynesia
NMNZ	LB19	Little Barrier	male	Polynesia
NMNZ	LB24	Little Barrier	male	Polynesia
NMNZ	LB25	Little Barrier	female	Polynesia
NMNZ	LB30	Little Barrier	male	Polynesia
NMNZ	LB31	Little Barrier	female	Polynesia
NMNZ	LB32	Little Barrier	female	Polynesia
NMNZ	LB33	Little Barrier	male	Polynesia
NMNZ	LB39	Little Barrier	female	Polynesia
NMNZ	LB40	Little Barrier	male	Polynesia
NMNZ	LB41	Little Barrier	female	Polynesia
NMNZ	LB44	Little Barrier	female	Polynesia
NMNZ	LB45	Little Barrier	female	Polynesia
NMNZ	LB52	Little Barrier	female	Polynesia
NMNZ	LB53	Little Barrier	male	Polynesia
NMNZ	LB54	Little Barrier	male	Polynesia
NMNZ	LB55	Little Barrier	male	Polynesia
NMNZ	E2	Macauley Island	male	Polynesia
NMNZ	E3	Macauley Island	female	Polynesia
NMNZ	AED469	Pitcairn	male	Polynesia
NMNZ	AED466	Pitcairn	male	Polynesia
NMNZ	AED467	Pitcairn	N/A	Polynesia
NMNZ	AED468	Pitcairn	male	Polynesia
NMNZ	LM142	Raoul Island	female	Polynesia
NMNZ	LM146	Raoul Island	female	Polynesia
NMNZ	LM764	Raoul Island	female	Polynesia
NMNZ	LM765	Raoul Island	N/A	Polynesia
NMNZ	LM766	Raoul Island	male	Polynesia
NMNZ	LM767	Raoul Island	female	Polynesia
NMNZ	LM768	Raoul Island	female	Polynesia
NMNZ	LM772	Raoul Island	female	Polynesia
NMNZ	LM773	Raoul Island	male	Polynesia
NMNZ	214	Rurima Rocks	N/A	Polynesia
NMNZ	215	Rurima Rocks	N/A	Polynesia
NMNZ	216	Rurima Rocks	N/A	Polynesia
NMNZ	217	Rurima Rocks	N/A	Polynesia
NMNZ	AED463	South Island	male	Polynesia

Institute	Specimen	Island	Sex	Biogeographical region		
NMNZ	AED464	South Island	male	Polynesia		
NMNZ	AED465	South Island	female	Polynesia		
NMNZ	LM770	South Island	N/A	Polynesia		
NMNZ	LM769	South Island	N/A	Polynesia		
NMNZ	LM771	South Island	N/A	Polynesia		dorsal pelage only
NMNZ	198	Stewart	male	Polynesia		
NMNZ	E4	Stewart	male	Polynesia		
NMNZ	1	North Island	N/A	Polynesia	first sac	
NMNZ	2	North Island	N/A	Polynesia	first sac	
NMNZ	3	North Island	N/A	Polynesia	first sac	
NMNZ	4	North Island	N/A	Polynesia	first sac	
NMNZ	5	North Island	N/A	Polynesia	first sac	
NMNZ	20	North Island	N/A	Polynesia	first sac	
NMNZ	36	North Island	N/A	Polynesia	first sac	
NMNZ	40	North Island	N/A	Polynesia	first sac	
NMNZ	52	North Island	N/A	Polynesia	first sac	
NMNZ	82	North Island	N/A	Polynesia	first sac	
NMNZ	83	North Island	N/A	Polynesia	first sac	
NMNZ	84 (1)	North Island	N/A	Polynesia	first sac	
NMNZ	84 (2)	North Island	N/A	Polynesia	first sac	
NMNZ	85	North Island	N/A	Polynesia	first sac	
NMNZ	86	North Island	N/A	Polynesia	first sac	
NMNZ	87	North Island	N/A	Polynesia	first sac	
NMNZ	88	North Island	N/A	Polynesia	first sac	
NMNZ	90	North Island	N/A	Polynesia	first sac	
NMNZ	92	North Island	N/A	Polynesia	first sac	
NMNZ	93	North Island	N/A	Polynesia	first sac	
NMNZ	97	North Island	N/A	Polynesia	first sac	
NMNZ	110	North Island	N/A	Polynesia	first sac	
NMNZ	112	North Island	N/A	Polynesia	first sac	
NMNZ	116	North Island	N/A	Polynesia	first sac	
NMNZ	85	North Island	N/A	Polynesia	second bag (with Nive)	
NMNZ	89	North Island	N/A	Polynesia	second bag (with Nive)	
NMNZ	91	North Island	N/A	Polynesia	second bag (with Nive)	
NMNZ	107	North Island	N/A	Polynesia	second bag (with Nive)	
NMNZ	1	North Island	male	Polynesia	third bag ("1974")	
NMNZ	4	North Island	female	Polynesia	third bag ("1974")	
NMNZ	8	North Island	female	Polynesia	third bag ("1974")	
NMNZ	21	North Island	male	Polynesia	third bag ("1974")	
NMNZ	24	North Island	female	Polynesia	third bag ("1974")	
NMNZ	25	North Island	male	Polynesia	third bag ("1974")	
NMNZ	26	North Island	female	Polynesia	third bag ("1974")	
NMNZ	27	North Island	female	Polynesia	third bag ("1974")	
NMNZ	28	North Island	female	Polynesia	third bag ("1974")	
NMNZ	70	North Island	male	Polynesia	fourth bag (first specimen 1977)	
NMNZ	73	North Island	male	Polynesia	fourth bag (first specimen 1977)	
NMNZ	34	North Island	male	Polynesia	sac LM 863	
NMNZ	34	North Island	male	Polynesia	sac LM 863	
NMNZ	40	North Island	N/A	Polynesia	sac LM 863	
NMNZ	40	North Island	N/A	Polynesia	sac LM 863	
NMNZ	41	North Island	N/A	Polynesia	sac LM 863	
NMNZ	41	North Island	N/A	Polynesia	sac LM 863	
NMNZ	42	North Island	N/A	Polynesia	sac LM 863	
NMNZ	42	North Island	N/A	Polynesia	sac LM 863	
NMNZ	43	North Island	N/A	Polynesia	sac LM 863	
NMNZ	43	North Island	N/A	Polynesia	sac LM 863	
NMNZ	44	North Island	N/A	Polynesia	sac LM 863	
NMNZ	44	North Island	N/A	Polynesia	sac LM 863	
NMNZ	45	North Island	N/A	Polynesia	sac LM 863	
NMNZ	46	North Island	N/A	Polynesia	sac LM 863	
NMNZ	48	North Island	N/A	Polynesia	sac LM 863	
NMNZ	48	North Island	N/A	Polynesia	sac LM 863	
NMNZ	49	North Island	N/A	Polynesia	sac LM 863	
NMNZ	49	North Island	N/A	Polynesia	sac LM 863	
NMNZ	50	North Island	N/A	Polynesia	sac LM 863	
NMNZ	51	North Island	N/A	Polynesia	sac LM 863	

Institute	Specimen	Island	Sex	Biogeographical region	
NMNZ	52	North Island	N/A	Polynesia	sac LM 863
NMNZ	52	North Island	N/A	Polynesia	sac LM 863
NMNZ	55	North Island	N/A	Polynesia	sac LM 863
NMNZ	55	North Island	N/A	Polynesia	sac LM 863
NMNZ	57	North Island	N/A	Polynesia	sac LM 863
NMNZ	59	North Island	N/A	Polynesia	sac LM 863
NMNZ	60 (1)	North Island	N/A	Polynesia	sac LM 863
NMNZ	60 (2)	North Island	N/A	Polynesia	sac LM 863
NMNZ	61	North Island	N/A	Polynesia	sac LM 863
NMNZ	64 (1)	North Island	N/A	Polynesia	sac LM 863
NMNZ	64 (2)	North Island	N/A	Polynesia	sac LM 863
NMNZ	65	North Island	N/A	Polynesia	sac LM 863
NMNZ	66 (1)	North Island	N/A	Polynesia	sac LM 863
NMNZ	66 (2)	North Island	N/A	Polynesia	sac LM 863
NMNZ	67 (1)	North Island	N/A	Polynesia	sac LM 863
NMNZ	67 (2)	North Island	N/A	Polynesia	sac LM 863
NMNZ	68 (1)	North Island	N/A	Polynesia	sac LM 863
NMNZ	68 (2)	North Island	N/A	Polynesia	sac LM 863
NMNZ	69	North Island	N/A	Polynesia	sac LM 863
NMNZ	71	North Island	N/A	Polynesia	sac LM 863
NMNZ	72	North Island	N/A	Polynesia	sac LM 863
NMNZ	73 (1)	North Island	N/A	Polynesia	sac LM 863
NMNZ	73 (2)	North Island	N/A	Polynesia	sac LM 863
NMNZ	74	North Island	N/A	Polynesia	sac LM 863
NMNZ	75	North Island	N/A	Polynesia	sac LM 863
NMNZ	78	North Island	N/A	Polynesia	sac LM 863
NMNZ	82	North Island	N/A	Polynesia	sac LM 863
NMNZ	n.n.	White Island (Whakaari)	N/A	Polynesia	mummified specimen
NMNZ	117	White Island (Whakaari)	male	Polynesia	
NMNZ	107	White Island (Whakaari)	female	Polynesia	
NMNZ	109	White Island (Whakaari)	female	Polynesia	
NMNZ	110	White Island (Whakaari)	male	Polynesia	
NMNZ	118	White Island (Whakaari)	male	Polynesia	
NMNZ	116	White Island (Whakaari)	male	Polynesia	
NMNZ	762	White Island (Whakaari)	male	Polynesia	
