# Supplementary material for: Effect of isolation on coat colour polymorphism of Polynesian rats in Island Southeast Asia and the Pacific
Source: PeerJ. 2019 May 8;7:e6894. doi: 10.7717/peerj.6894 (PMC6511229; doi:10.7717/peerj.6894)
Supplement: Data S2 [file peerj-07-6894-s002.pdf]

Effect of isolation on coat colour polymorphism of wild populations of Polynesian rats (*Rattus exulans*) in Island Southeast Asia and the Pacific

Alexandra A.E. van der Geer

Naturalis Biodiversity Center
Leiden
the Netherlands

alexandra.vandergeer@naturalis.nl

DATA FILE S2

This file contains island features of the studied islands.

Explanation of column headers

PREDATORS: number of ecologically relevant native predators.
COMPETITORS: number of ecologically relevant native competitors.
AREA: island area in km^2.
TIME CLASS: time in isolation, classified in time bins of 1,000 years.

References for TIME CLASS

Anderson A. 2005. Crossing the Luzon Strait: archaeological chronology in the Batanes Islands, Philippines and the regional sequence of Neolithic dispersal. *Journal of Austronesian Studies* **1**:25–44.

Anderson A. 2008. Traditionalism, interaction, and long-distance seafaring in Polynesia. *Journal of Island and Coastal Archaeology* **3**:240–250. DOI 10.1080/15564890802340000

Anderson A., Sinoto Y. 2002. New radiocarbon dates of colonization sites in East Polynesia. *Asian Perspectives* **41**:242–257.

Anderson A., White P. 2001. Prehistoric settlement on Norfolk Island and its Oceanic context. *Records of the Australian Museum, Supplement* **27**:135–141.

Burley DV, Weisler M, Zhao XJ. 2012. High precision U/Th dating of first polynesian settlement. *PLoSONE* **7 (11)**:e48769. DOI 10.1371/journal.pone.0048769

Carson MT, Hung H-C, Summerhayes G, Bellwood P. 2013. The pottery trail from Southeast Asia to Remote Oceania. *Journal of Island and Coastal Archaeology* **8**:17–36. DOI 10.1080/15564894.2012.726941

Clark JT, Quintus S, Weisler M, St Pierre E, Nothdurft L, Feng Y. 2016. Refining the chronology for West Polynesian colonization: new data from the Samoan archipelago. *Journal of Archaeological Science Reports* **6**:266–274. DOI 10.1016/j.jasrep.2016.02.011

Cranbrook Earl of. 2000. Northern Borneo environments of the past 40,000 years: archaeozoological evidence. *The Sarawak Museum Journal* **55**:61–109.

Denham T, Ramsey CB, Specht J. 2012. Dating the appearance of Lapita pottery in the Bismarck Archipelago and its dispersal to Remote Oceania. *Archaeology in Oceania* **47**:39–46. DOI 10.1002/j.1834-4453.2012.tb00113.x

Green RC, Jones M, Sheppard P. 2008. The reconstructed environment and absolute dating of SE-SZ-8 Lapita site on Nendö, Santa Cruz, Solomon Islands. *Archaeology in Oceania* **43**:49–61. DOI 10.1002/j.1834-4453.2008.tb00030.x

Hanebuth TJJ, Voris HK, Yokoyama Y, Saito Y, Okuno JI. 2011. Formation and fate of sedimentary depocenters on Southeast Asia's Sunda Shelf over the past sea-level cycle and biogeographic implications. *Earth-Science Reviews* **104**:92–110.

Kirch PV. 1984. *The Evolution of Polynesian Chiefdoms*. Cambridge, UK: Cambridge University Press.

McCoy MD, Alerson HA, Hemi R, Cheng H, Lawrence Edwards R. 2016. Earliest direct evidence of monument building at the archaeological site of Nan Madol (Pohnpei, Micronesia) identified using ^{230}Th/U coral dating and geochemical sourcing of megalithic architectural stone. *Quaternary Research* **86**:295–303. DOI 10.1016/j.ygres.2016.08.002

Summerhayes GR, Leavesley M, Fairbairn A, Mandui H, Field J, Ford A, Fullagar R. 2010. Human adaptation and plant use in highland New Guinea 49,000 to 44,000 years ago. *Science* **330**:78–81. DOI 10.1126/science.1193130

West K, Collins C, Kardailsky O, Kahn J, Hunt TL, Burley DV, Matisoo-Smith E. 2017. The Pacific rat race to Easter Island: tracking the prehistoric dispersal of *Rattus exulans* using ancient mitochondrial genomes. *Frontiers in Ecology and Evolution* **5**:52. DOI 10.3389/fevo.2017.00052

Wilmshurst JM, Hunt TL, Lipo CP, Anderson AJ. 2011. High-precision radiocarbon dating shows recent and rapid initial human colonization of East Polynesia. *Proceedings of the National Academy of Sciences of the United States of America* **108**:1815–1820.

ISLAND	REGION	PREDATORS	COMPETITORS	AREA	TIME CLASS	SOURCE FOR TIME
Ambon	Wallacea	0	0	805.8	4	Anderson 2005
And Atoll	Micronesia	0	0	0.35	1	West et al. 2017
Arno atoll	Micronesia	0	0	13	1	West et al. 2017
Atafu	Polynesia	0	0	2.2	2	Anderson, 2008
Aunuu	Polynesia	0	0	1.6	3	Clark et al. 2016
Babelthuap	Micronesia	0	0	374.1	2	inferred from Pohnpei data
Balabac	Sunda	1	3	318.8	5	Hanebuth et al. 2011
Bali	Sunda	3	2	5416.4	5	Hanebuth et al. 2011
Bali Kukup	Sunda	N/A	N/A	0.182	5	Hanebuth et al. 2011
Banka	Sunda	N/A	N/A	11413.3	5	Hanebuth et al. 2011
Basilan	Wallacea	1	1	1265.5	4	inferred from Luzon
Batam	Sunda	0	0	399.1	5	Hanebuth et al. 2011
Bikar Atoll	Micronesia	0	0	0.5	1	West et al. 2017
Bikini	Micronesia	0	0	6	1	West et al. 2017
Birnie	Polynesia	0	0	0.2	1	inferred from Line Islands
Borneo	Sunda	15	3	748168.1	5	Hanebuth et al. 2011
Bougainville	Melanesia	0	3	9317.8	4	as Bismarck
Buru	Wallacea	0	0	8473.2	4	Anderson 2005
Busuanga	Wallacea	2	3	980	4	inferred from Northern Luzon data
Carlos Island	Micronesia	0	0	N/A	N/A	
Catanduanes	Wallacea	0	3	1522.9	4	inferred from Northern Luzon data
David Island	Micronesia	0	0	N/A	N/A	
Dick Island	Micronesia	0	0	N/A	N/A	
Éfaté	Outlier	0	0	899.5	3	Kirch (1984)
Emirau	Melanesia	0	0	41	4	Green et al. 2008; Summerhayes et al. 2010
Enewetak	Micronesia	0	0	5.8	1	West et al. 2017
Espiritu Santo	Melanesia	0	0	3955.5	4	Denham et al. 2012
Fakaofo	Polynesia	0	0	2.6	2	Anderson, 2008
Fergusson	Melanesia	0	6	1436.7	4	as Bismarck
Fiji	Melanesia	0	0	10531	3	Denham et al. 2012
Goodenough	Melanesia	0	4	686.7	4	as Bismarck
Grande Terre	Melanesia	0	0	16648.4	4	as Bismarck
Guadalcanal	Melanesia	0	3	5352.9	4	as Bismarck
Guam	Micronesia	0	0	541	4	Carson et al. 2013
Guguan	Micronesia	0	0	4	4	Carson et al. 2013
Halmahera	Wallacea	0	3	18039.6	4	Anderson 2005
Hatutaa	Polynesia	0	0	18.1	1	Wilmshurst et al., 2011
Hawaii	Polynesia	0	0	10435	1	Wilmshurst et al. 2011
Hen Island	Polynesia	0	0	4.7	1	Wilmshurst et al. 2011
Java	Sunda	13	5	138793.6	5	Hanebuth et al. 2011
Kalap Island	Micronesia	0	0	1.3	2	inferred from Pohnpei data
Kanton	Polynesia	0	0	9.1	1	inferred from Line Islands
Kapingamarangi	Outlier	0	0	1.1	3	Kirch (1984)
Kauai	Polynesia	0	0	1434.6	1	Wilmshurst et al. 2011
Kayangel	Micronesia	0	0	1.7	2	inferred from Pohnpei data
Kiritimati	Polynesia	0	0	321	1	Wilmshurst et al., 2011
Kiriwinia	Melanesia	0	0	266.5	4	as Bismarck
Koror	Micronesia	0	0	9.3	2	inferred from Pohnpei data
Kosrae	Micronesia	0	0	110	2	inferred from Pohnpei data
Kure Atoll	Polynesia	0	0	0.77685	1	Wilmshurst et al. 2011
Kwajalein Atoll	Micronesia	0	0	16	1	West et al. 2017
Leyte	Wallacea	0	4	7367.6	4	inferred from Northern Luzon data
Little Barrier	Polynesia	0	0	28	1	Wilmshurst et al. 2011
Luzon	Wallacea	0	40	109964.9	4	Anderson 2005
Macauley	Polynesia	0	0	3.7	1	Wilmshurst et al. 2011
Majuro	Micronesia	0	0	9	1	West et al. 2017

Malenge	Wallacea	N/A	N/A	13.38	4	inferred from Sulawesi data
Manra Island	Polynesia	0	0	4.4	1	inferred from Line Islands
Maui	Polynesia	0	0	1903.3	1	Wilmshurst et al. 2011
Mindanao	Wallacea	0	13	97530	4	inferred from Northern Luzon data
Mindoro	Wallacea	0	6	10571.8	4	inferred from Northern Luzon data
Misima Island	Melanesia	0	0	202.5	4	as Bismarck
Moen Island	Micronesia	0	0	18.8	2	inferred from Pohnpei data
Negros	Wallacea	0	1	13074.5	4	inferred from Northern Luzon data
New Britain	Melanesia	0	3	35144.6	4	Green et al. 2008; Summerhayes et al. 2010
New Guinea	Melanesia	3	73	785753	4	as Bismarck
New Ireland	Melanesia	0	1	7404.5	4	Green et al. 2008; Summerhayes et al. 2010
Nias Island	Sunda	N/A	N/A	4048	5	Hanebuth et al. 2011
Niuafo'Ou Island	Polynesia	0	0	52.3	3	Anderson et al. 2001
Normanby	Melanesia	0	5	1040	4	as Bismarck
North Island	Polynesia	0	0	111582.8	1	Wilmshurst et al. 2011
Nukunonu	Polynesia	0	0	5.4	2	Anderson, 2008
Nusi Island	Melanesia	0	0	5	4	as Bismarck
O'ahu	Polynesia	0	0	1583.3	1	Wilmshurst et al. 2011
Ofu	Polynesia	0	0	7.5	3	Burley et al. 2012
Olosega	Polynesia	0	0	5.4	3	Burley et al. 2012
Onotoa Atoll	Polynesia	0	0	13.5	1	inferred from Line Islands
Ontong Java	Melanesia	0	0	12	4	as Bismarck
Orona	Polynesia	0	0	3.9	1	inferred from Line Islands
Palawan	Sunda	5	6	12188.6	5	Cranbrook 2000
Panay	Wallacea	0	2	12011.1	4	inferred from Northern Luzon data
Peleliu Island	Micronesia	0	0	12.7	2	inferred from Pohnpei data
Peleng	Wallacea	N/A	N/A	2345.6	4	Anderson, 2005
Pitcairn	Polynesia	0	0	4.2	1	Anderson et al. 2002
Pohnpei	Micronesia	0	0	334	2	McCoy et al., 2016
Polillo Island	Wallacea	0	1	628.9	4	inferred from Northern Luzon data
Ponam	Melanesia	0	0	1.4	4	as Bismarck
Raoul	Polynesia	0	0	29.8	1	Wilmshurst et al. 2011
Raroia Atoll	Polynesia	0	0	9	1	West et al. 2017
Repanga	Polynesia	0	0	1.94	1	Wilmshurst et al. 2011
Rongelap	Micronesia	0	0	7.8	1	West et al. 2017
Rose Atoll	Polynesia	0	0	0.1	3	Burley et al. 2012
Rossel	Melanesia	0	0	262.5	4	as Bismarck
Rurima	Polynesia	0	0	0.045	1	Wilmshurst et al., 2011
Saipan	Micronesia	0	0	122.9	4	Carson et al. 2013
Samar	Wallacea	0	2	12849.4	4	inferred from Northern Luzon data
Simeulue	Sunda	0	2	1754.1	5	Hanebuth et al. 2011
South Island	Polynesia	0	0	145836.4	1	Wilmshurst et al., 2011
Stewart	Polynesia	0	0	1814.7	1	Wilmshurst et al., 2011
Sulawesi	Wallacea	1	36	180680.7	4	Anderson, 2005
Sumatra	Sunda	13	13	443065.8	5	Hanebuth et al. 2011
Swains	Polynesia	0	0	3.6	3	Burley et al. 2012
Tahiti	Polynesia	0	0	1068.8	1	Wilmshurst et al. 2011
Taka Atoll	Micronesia	0	0	3.4	1	
Taliabu	Wallacea	0	3	2913.2	4	Anderson 2005
Ta'u	Polynesia	0	0	45.7	3	as Ofu, Olosega
Tepoto	Polynesia	0	0	4	1	West et al. 2017
Tinian Island	Micronesia	0	0	102	4	Carson et al. 2013
Ugi Island	Melanesia	0	0	43.98	4	as Bismarck
Ulithi Atoll	Micronesia	0	0	4.5	2	inferred from Pohnpei data
Vanatinai	Melanesia	0	3	830	4	as Bismarck
Vostok	Polynesia	0	0	0.2	1	Wilmshurst et al., 2011
Wake Island	Micronesia	0	0	7.1	N/A	
Whakaari	Polynesia	0	0	3.25	1	Wilmshurst et al., 2011
Woodlark	Melanesia	0	2	873.9	4	as Bismarck
