# Supplementary material for: Effect of isolation on coat colour polymorphism of Polynesian rats in Island Southeast Asia and the Pacific
Source: PeerJ. 2019 May 8;7:e6894. doi: 10.7717/peerj.6894 (PMC6511229; doi:10.7717/peerj.6894)
Supplement: Table S1 — The analyses were performed using Past 3.16 (Hammer, Harper & Ryan, 2001). [file peerj-07-6894-s003.docx]

| **SUMMARY STATISTICS** | | | | | |
| --- | --- | --- | --- | --- | --- |
|  | **native predators** | **native competitors** | **log^10^ surface area (km^2^)** | **time bin** | **aberrant coats** |
| N | 110 | 110 | 112 | 111 | 115 |
| Min | 0 | 0 | -1.34679 | 1 | 0 |
| Max | 15 | 73 | 5.895286 | 5 | 2 |
| Sum | 57 | 255 | 243.4256 | 313 | 14 |
| Mean | 0.5181818 | 2.318182 | 2.173443 | 2.81982 | 0.121739 |
| Std. error | 0.2205958 | 0.8308011 | 0.162478 | 0.1397308 | 0.035256 |
| Variance | 5.352877 | 75.92535 | 2.956688 | 2.16724 | 0.142944 |
| Stand. dev | 2.313629 | 8.713516 | 1.719502 | 1.472155 | 0.37808 |
| Median | 0 | 0 | 2.197988 | 3 | 0 |
| 25 percentile | 0 | 0 | 0.707326 | 1 | 0 |
| 75 percentile | 0 | 1.25 | 3.563994 | 4 | 0 |
| Skewness | 5.309198 | 6.306174 | 0.176912 | -0.1174809 | 3.277397 |
| Kurtosis | 28.38465 | 44.48883 | -0.8788 | -1.590915 | 10.88542 |
| Geom. mean | 0 | 0 | 0 | 2.358313 | 0 |
| Coeff. var | 446.4897 | 375.8771 | 79.11421 | 52.20741 | 310.5655 |
|  |  |  |  |  |  |
| **NORMALITY TESTS: no variable is normally distributed (p lower than 0.05)** | | | | | |
| N | 110 | 110 | 112 | 111 | 115 |
| Shapiro-Wilk W | 0.2347 | 0.2759 | 0.9675 | 0.8178 | 0.3559 |
| p(normal) | 2.18E-21 | 7.02E-21 | 7.84E-03 | 2.14E-10 | 3.00E-20 |
| Anderson-Darling A | 35.36 | 34.7 | 1.451 | 8.525 | 35.33 |
| p(normal) | 3.24E-78 | 6.14E-77 | 9.06E-04 | 7.49E-21 | 3.93E-78 |
| p(Monte Carlo) | 0.0001 | 0.0001 | 0.0008 | 0.0001 | 0.0001 |
| Jarque-Bera JB | 3858 | 8964 | 4.291 | 11.7 | 715.4 |
| p(normal) | 0 | 0 | 0.117 | 0.002876 | 4.46E-156 |
| p(Monte Carlo) | 0.0001 | 0.0001 | 0.0821 | 0.0122 | 0.0001 |
