# Supplementary material for: Effect of isolation on coat colour polymorphism of Polynesian rats in Island Southeast Asia and the Pacific
Source: PeerJ. 2019 May 8;7:e6894. doi: 10.7717/peerj.6894 (PMC6511229; doi:10.7717/peerj.6894)
Supplement: Table S2 — The analyses were performed using Past 3.16 (Hammer, Harper & Ryan, 2001). [file peerj-07-6894-s004.docx]

| **Normal versus abnormal coat patterns in Polynesian rats, regional distribution** | | | |
| --- | --- | --- | --- |
| **Chi squared** |  |  |  |
| **Rows, columns** | 6,2 | **Degrees freedom** | 5 |
| **Chi^2^** | 9.8818 | ***p*** | 0.078 |
| **Monte Carlo *p*** | 0.070 |  |  |
|  |  |  |  |
| **Normal versus abnormal, melanisme and leucisme considered separately** | | | |
| **Chi squared** |  |  |  |
| **Rows, columns** | 6,3 | **Degrees freedom** | 10 |
| **Chi^2^** | 17.602 | ***p*** | 0.062 |
| **Monte Carlo *p*** | 0.0107 |  |  |
|  |  |  |  |
| **Normal versus abnormal coat patterns, mainland versus islands** | | | |
| **Chi squared** |  |  |  |
| **Rows, columns** | 2,2 | **Degrees freedom** | 1 |
| **Chi^2^** | 1.9535 | ***p*** | 0.162 |
| **Monte Carlo *p*** | 0.2405 |  |  |
|  |  |  |  |
| **Relation between number of native predators and aberrant coats** | | | |
| **Chi squared** |  |  |  |
| **Rows, columns** | 4,3 | **Degrees freedom** | 6 |
| **Chi^2^** | 15.422 | ***p*** | 0.017 |
| **Monte Carlo *p*** | 0.0953 |  |  |
|  |  |  |  |
| **Relation between number of native competitors and aberrant coats** | | | |
| **Chi squared** |  |  |  |
| **Rows, columns** | 4,3 | **Degrees freedom** | 6 |
| **Chi^2^** | 21.023 | ***p*** | 0.002 |
| **Monte Carlo *p*** | 0.0155 |  |  |
|  |  |  |  |
| **Relation between time in isolation and aberrant coats** | | | |
| **Chi^2^** |  |  |  |
| **Rows, columns** | 5,3 | **Degrees freedom** | 8 |
| **Chi^2^** | 5.7713 | ***p*** | 0.673 |
| **Monte Carlo *p*** | 0.7248 |  |  |
|  |  |  |  |
| **Relation with island area** | | | |
| **Kruskal Wallis Test, non-parametric** | | **Aberrant coats** | |
| **Chi^2^** | 6,070 | **Asymp. Sig.** | 0.048 |
| **df** | 2 |  |  |
| **Kruskal Wallis Test, non-parametric** | | **Competitors** |  |
| **Chi^2^** | 47,175 | **Asymp. Sig.** | 0,000 |
| **df** | 11 |  |  |
