# Supplementary figures and images for: Effect of isolation on coat colour polymorphism of Polynesian rats in Island Southeast Asia and the Pacific
Source: PeerJ. 2019 May 8;7:e6894. doi: 10.7717/peerj.6894 (PMC6511229; doi:10.7717/peerj.6894)

A

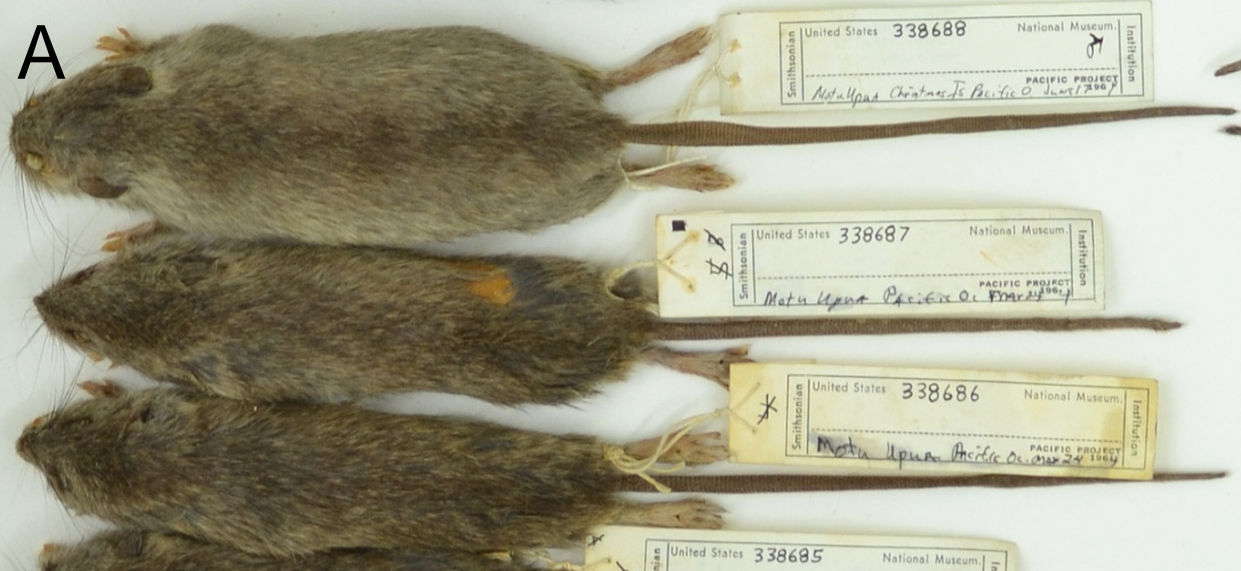

B

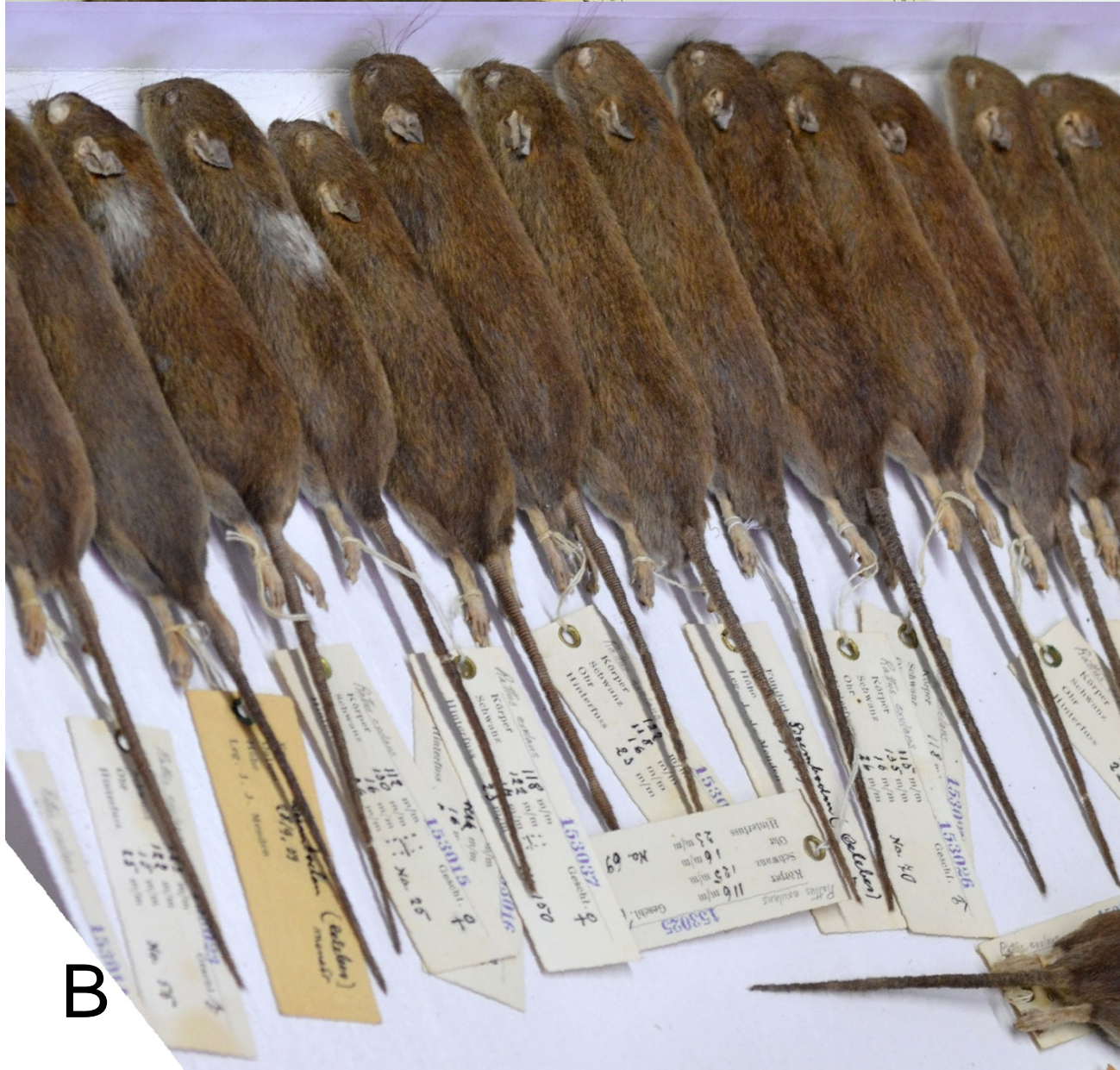

Supplement: Figure S1 — (A) Evenly coloured ash grey specimen from Kiritimati, Line Islands (USNM 338688), compared to two normal-coloured specimens from the same location. (B) Two specimens with white patches at the shoulder region from Sulawesi (USNM 153015, USNM 153037). Photo credits: Bartholomeus van der Geer. [file peerj-07-6894-s005.pdf]

A

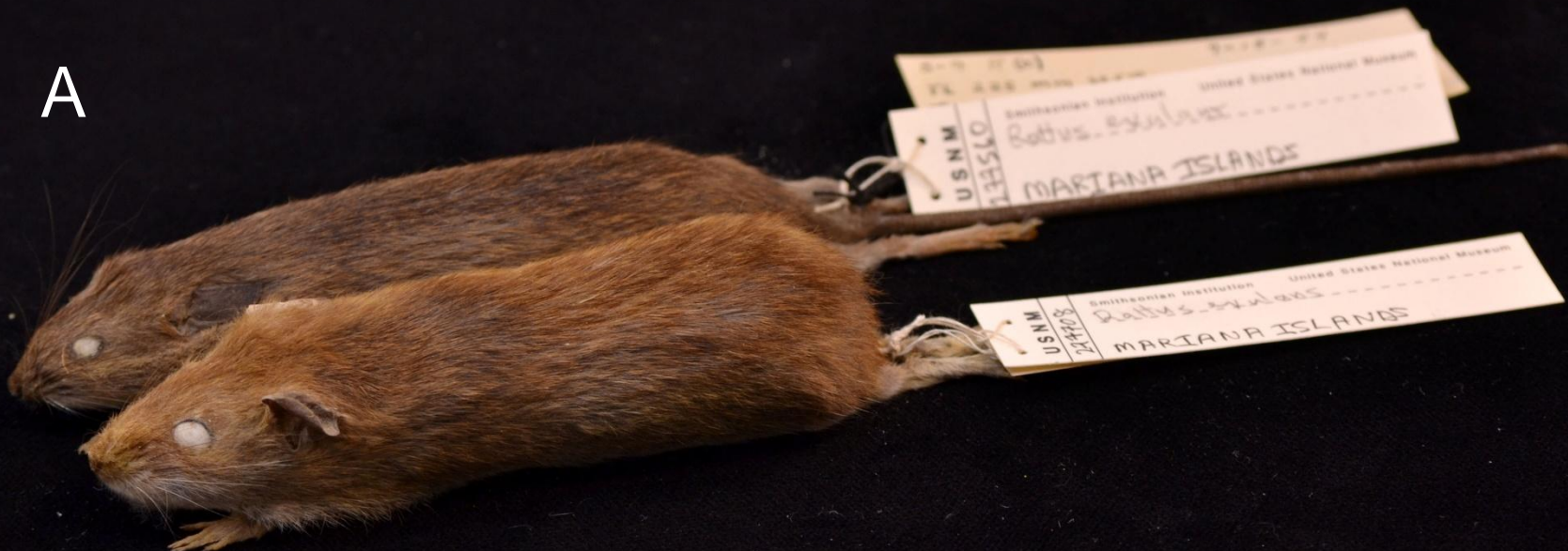

B

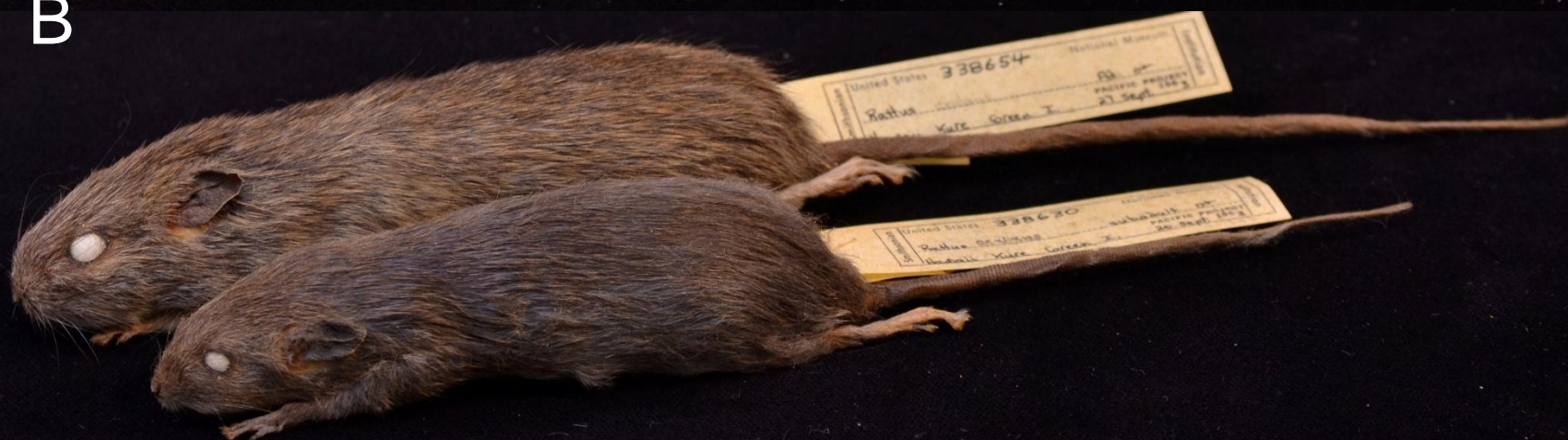

C

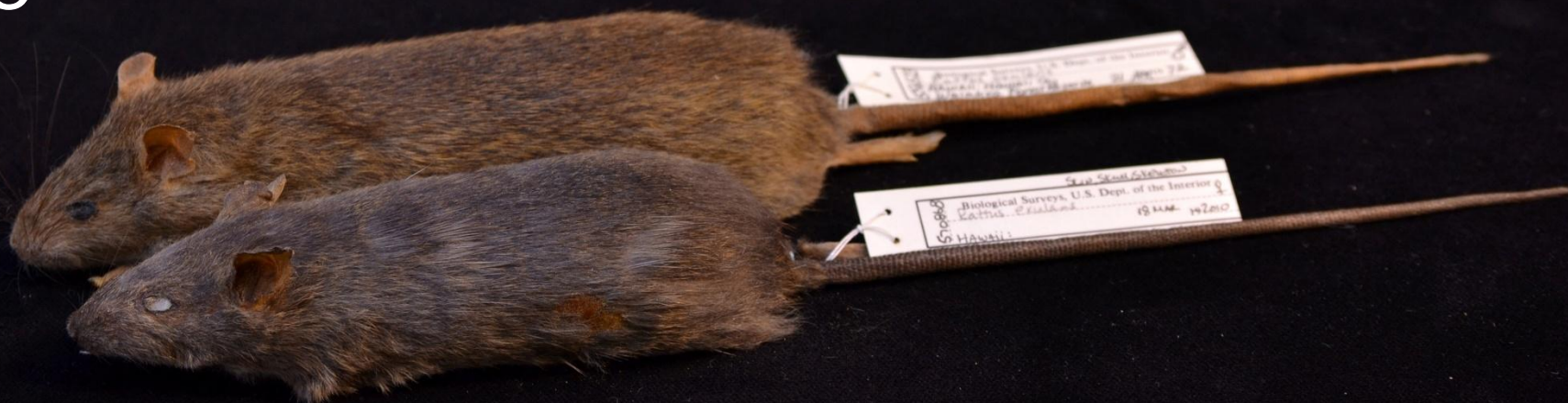

Supplement: Figure S2 — (A) Hazelnut (dark buff) coloured specimen from Saipan, Marianas (USNM 277708; female), here considered a phase of leucism (decreased melanin expression). (B) Melanistic specimen from Kure atoll, Hawaii (USNM 338630; female). (C) Melanistic specimen from Kauai, Hawaii (USNM 570868) with a few isolated strands of white hair. Undersides of (B) and (C) are normal. (A–C) All specimens shown against a normal-coloured specimen from the same population in the back. Photo credits: Bartholomeus van der Geer. [file peerj-07-6894-s006.pdf]
